# Supplementary material for: Machine learning and structural analysis of Mycobacterium tuberculosis pan-genome identifies genetic signatures of antibiotic resistance
Source: Nat Commun. 2018 Oct 17;9:4306. doi: 10.1038/s41467-018-06634-y (PMC6193043; doi:10.1038/s41467-018-06634-y)
Supplement: Supplementary file 1 — Supplementary Information [file 41467_2018_6634_MOESM1_ESM.pdf]

Machine learning and structural analysis of *Mycobacterium tuberculosis* pan-genome identifies genetic signatures of antibiotic resistance

Kavvas et al.

Supplementary Figures

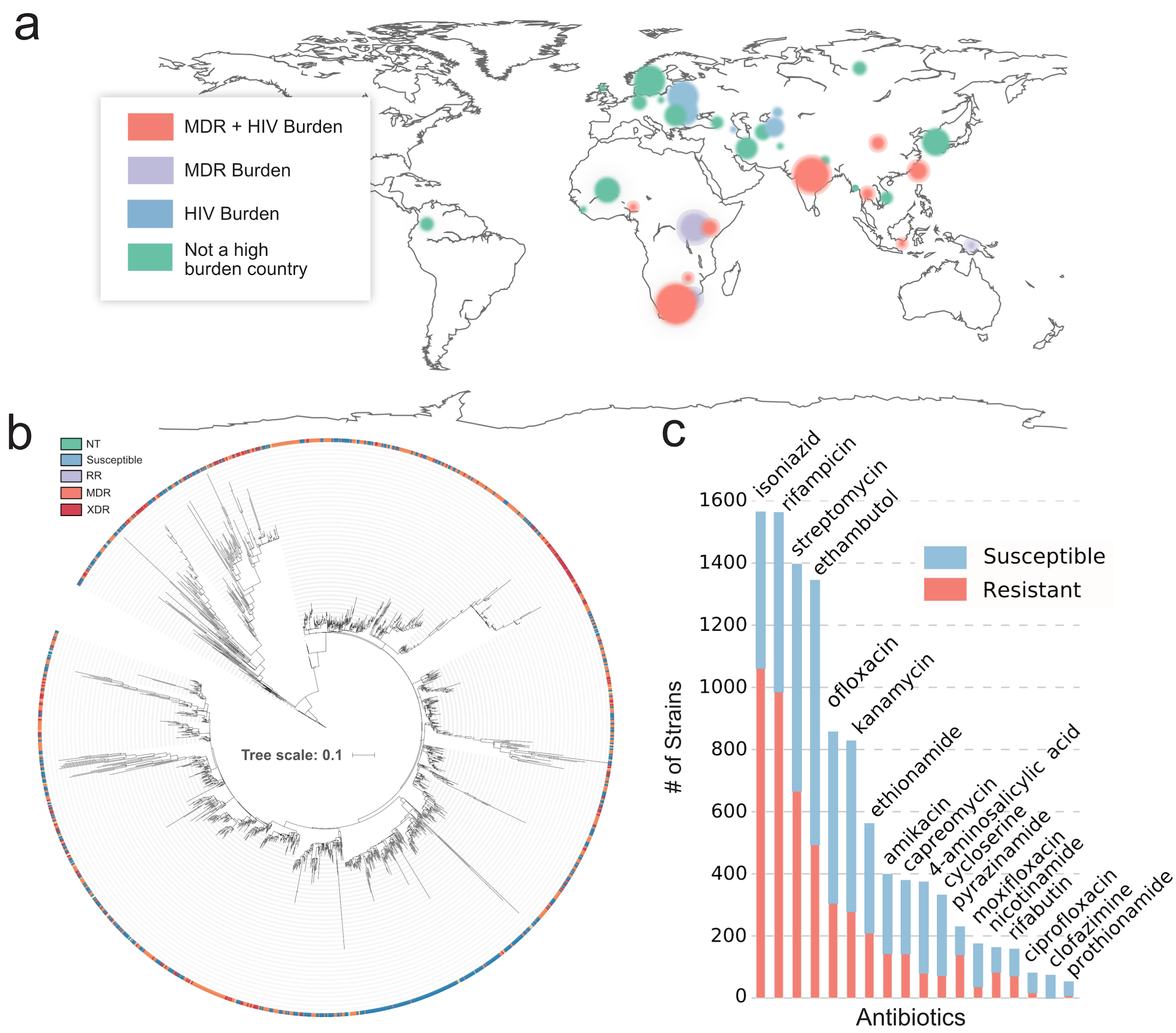

Supplementary Figure 1: Characteristics of 1595 strain dataset

M. *tuberculosis* strains were selected to span geography, resistances and phylogenetic space. **(a)** Geographic locations of strain isolation sites. The locations are colored according to the "high burden countries" 2016-2020 watchlist categories [1]. The size of the circles scale logarithmically with the number of strains found in that location. **(b)** Phylogenetic tree of the 1595 strains (**Methods**). **(c)** Specific drug characteristics tested across all 1595 strains. Abbreviations: RR, Rifampicin Resistant; MDR, Multidrug resistant; XDR, Extensively Drug Resistant; NT, Not Tested.

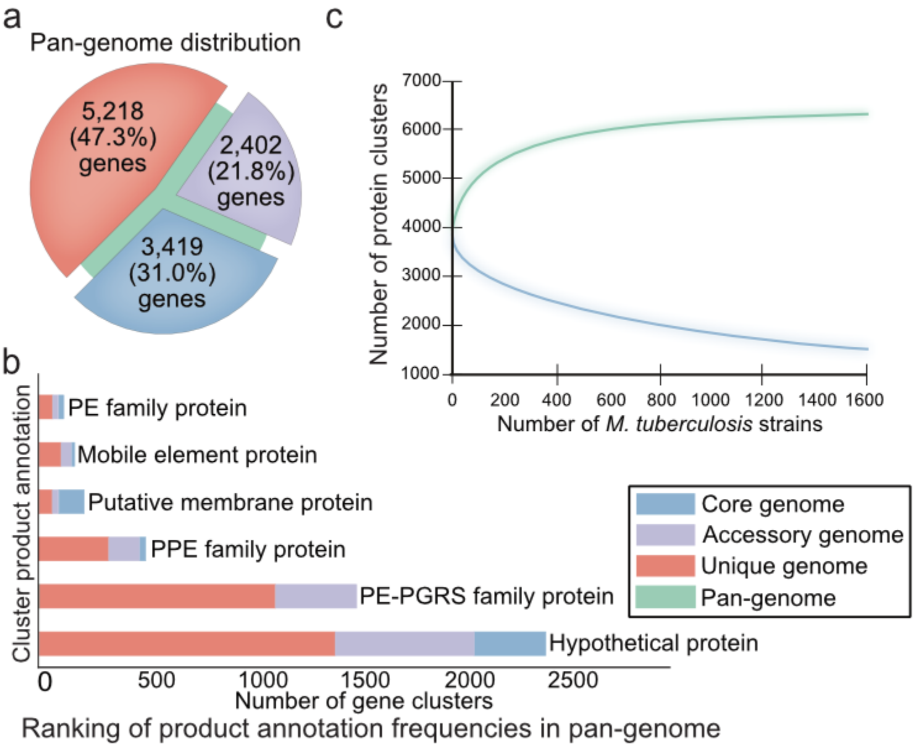

Supplementary Figure 2: M. *tuberculosis* pan-genome characteristics

(a) Distribution of the core, unique, and accessory genes across the pan-genome. **(b)** Products annotated across the pan-genome clusters in ranked order. **(c)** The number of protein clusters in the pan-genome against the number of M. *tuberculosis* strains. The green line indicates the size of the pan-genome as M. *tuberculosis* strains are added to the pan-genome. The blue line indicates the size of the core genome with addition of new strains.

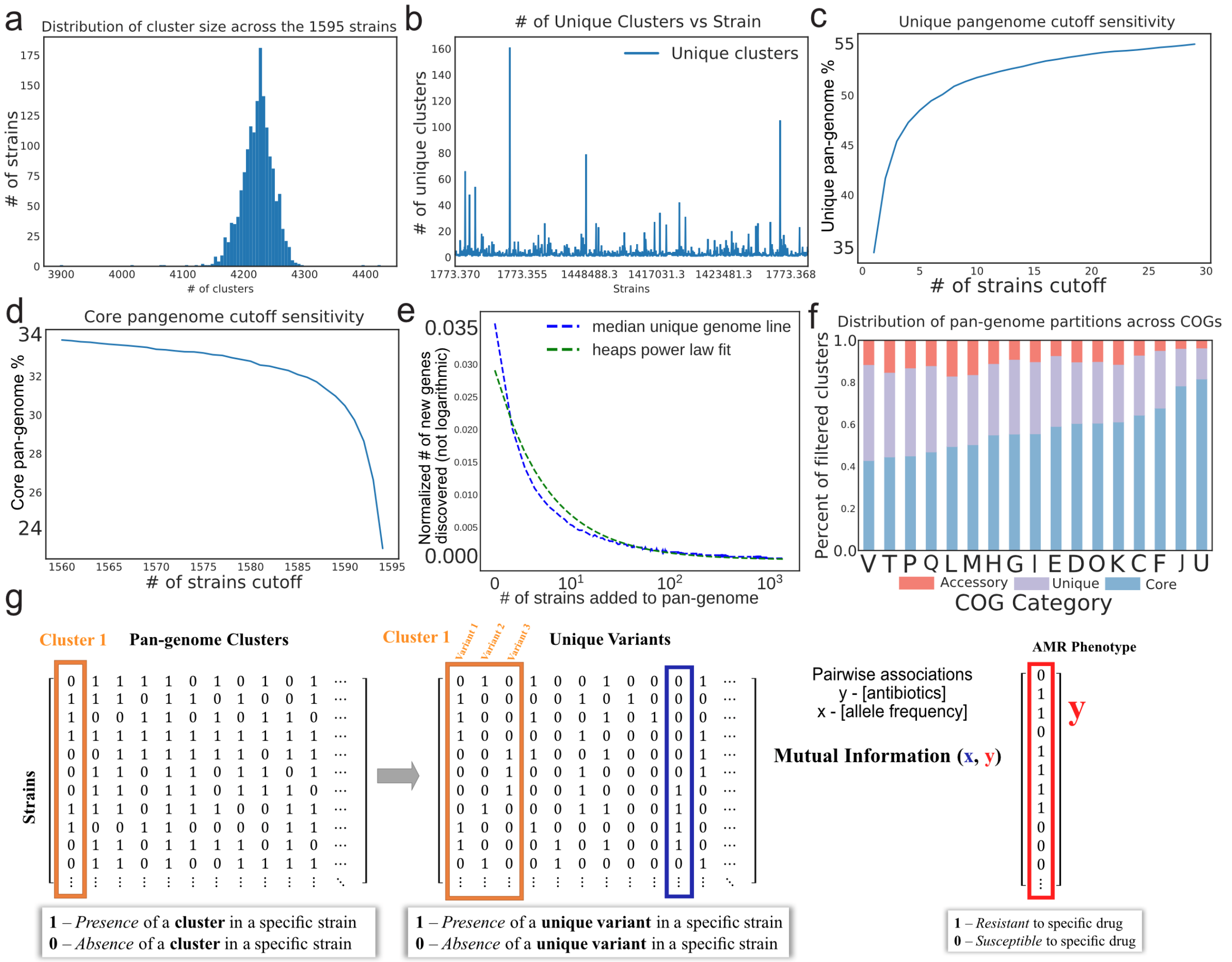

Supplementary Figure 3: Pan-genome quality check, characteristics, and allele-centric view.

(a) Distribution of *M. tuberculosis* luster size across the 1595 strains. **(b)** Number of unique clusters per strain in our dataset. **(c)** Change in unique pan-genome percentage according to change in strain cutoff values. **(d)** Change in core pan-genome percentage according to strain cutoff values. **(e)** Fit of median unique genome line on Heap's power law. The y-axis is the normalized # of new genes discovered (note that this axis is not logarithmic). The x-axis is a logarithmic number of strains added to pan-genome. **(f)** Distribution of the functional characterized pan-genome across COG categories. **(g)** Higher resolution view of genetic variation and subsequent calculation of pairwise associations. The allele pan-genome was constructed by separating out sequences of exact similarity (i.e. 100% amino acid conservation) into separate columns. Therefore, each column in the allele pan-genome matrix corresponds to the frequency of a unique allele across the 1595 strains. Alleles that were found in less than 5 strains were taken out of the analysis. The mutual information between each binary absence/presence allele vector (blue) and each AMR phenotype vector (red) was taken.

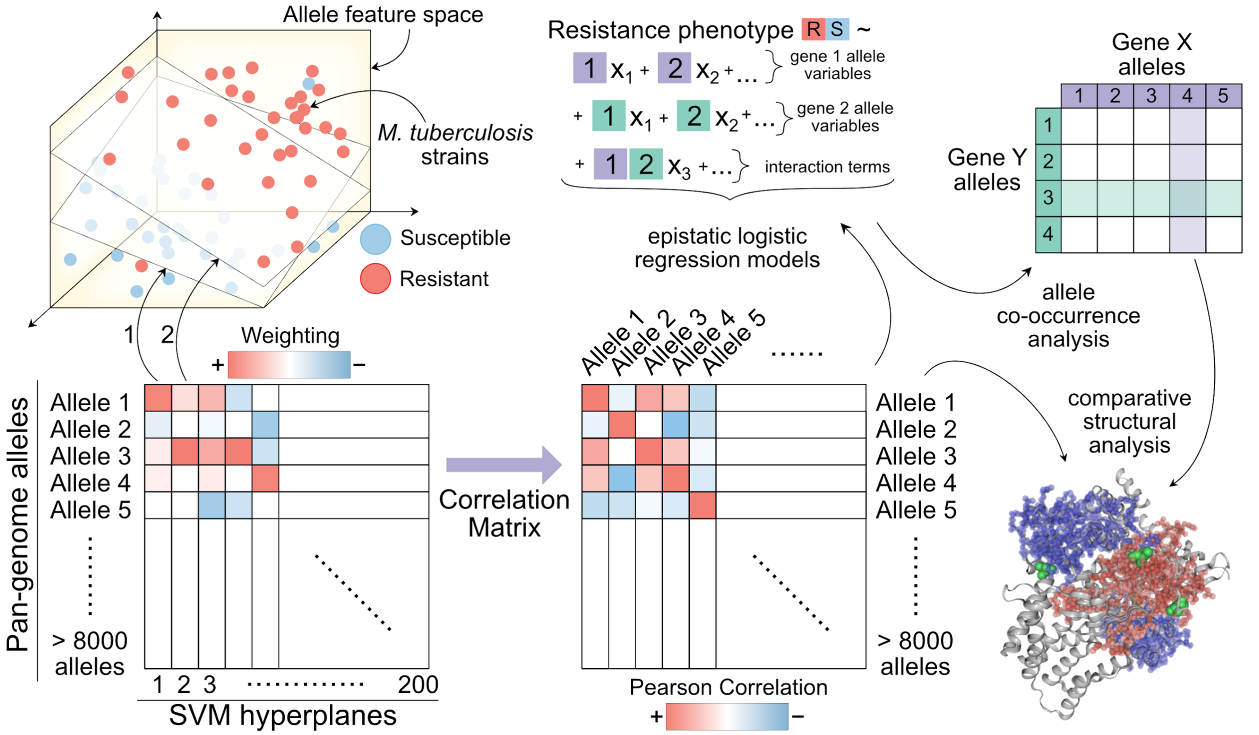

Supplementary Figure 4: Illustration of multi-layered analysis workflow.

A support vector machine (SVM) was trained on random subsets of the total population with equal size (i.e., bootstrapping). The SVM utilized an L1-norm and stochastic gradient descent (SGD). Due to the randomness and L1-norm, the SVM may choose different features with different weights for each subset. Correlation matrix between the alleles was determined from the ensemble of SVMs. Large positive correlations correspond to alleles whose weights often appear together and are of the same sign (i.e., positive and positive, or negative and negative). Large negative correlations correspond to alleles whose weights often appear together but are of different signs (i.e., positive and negative). Significant correlations were evaluated using logistic regression models and visualized using allele co-occurrence tables. Mapping alleles of both high ranked genes and correlated genes concluded the quantitative analysis.

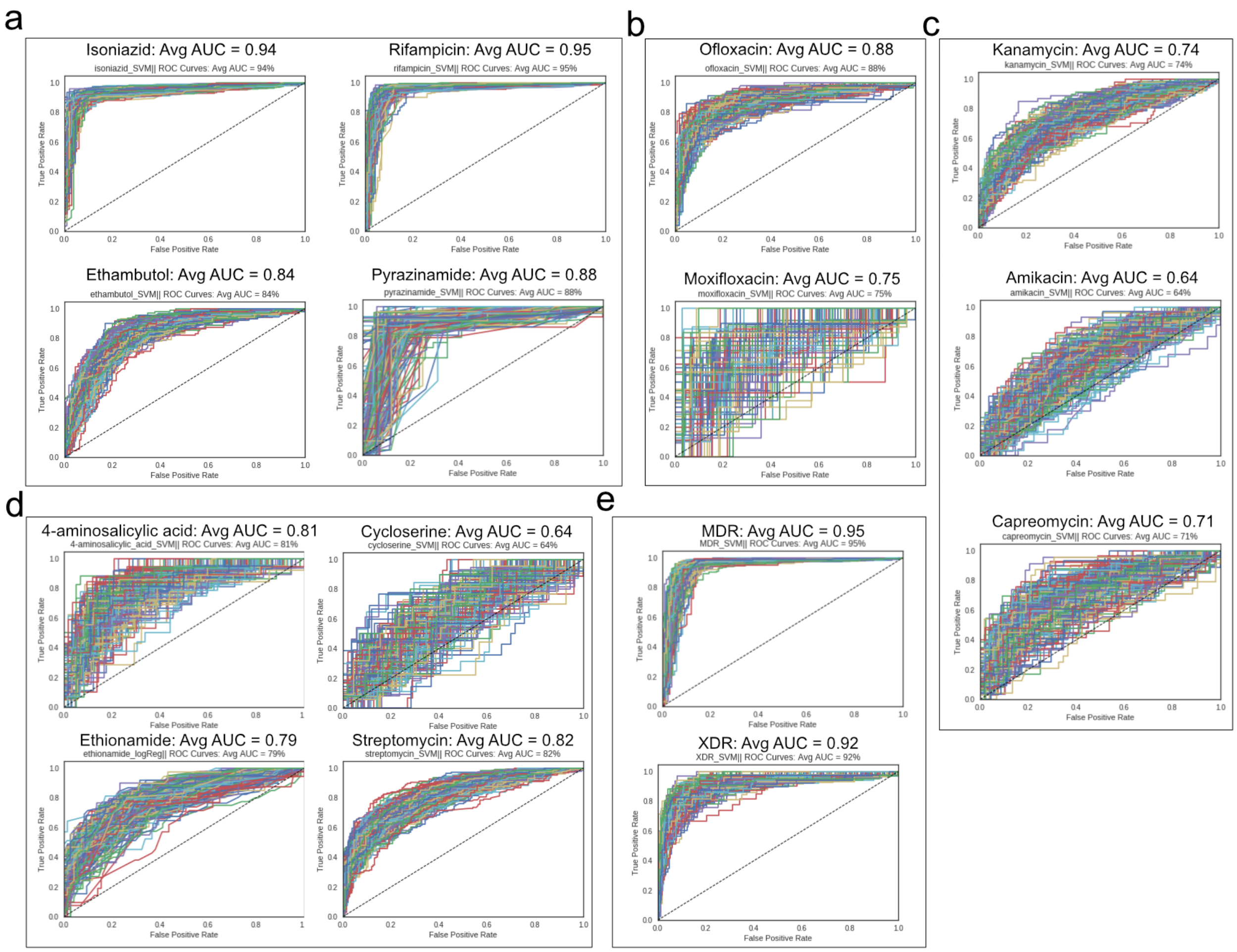

Supplementary Figure 5: Ensemble ROC curves for SGD-SVM predictions

Ensemble ROC curves for SGD-SVM (stochastic gradient descent support vector machine) predictions of different AMR classifications. **(a)** First-line drugs: isoniazid, rifampicin, ethambutol, and pyrazinamide. **(b)** Second-line drugs of fluoroquinolones: ofloxacin and moxifloxacin, and **(c)** aminoglycosides: kanamycin, amikacin, capreomycin. **(d)** Other antibiotics: 4-aminosalicylic acid, cycloserine, ethionamide, streptomycin. **(e)** MDR (multidrug resistant) and XDR (extensively drug resistant) classification. MDR is defined as M. *tuberculosis* strains that are resistant to at least Isoniazid and Rifampicin. XDR is defined as M. *tuberculosis* strains that are MDR and resistant to at least one second line aminoglycoside (i.e., amikacin, kanamycin, or capreomycin) and resistant to at least one second line fluoroquinolones (i.e. ciprofloxacin, ofloxacin, moxifloxacin). The average AUC was calculated by averaging over AUCs for the 200 independent SGD-SVM ROC curves. The y-axis is the true positive rate and the x-axis is the false positive rate. For ethionamide, a logistic regression estimator using both an L1-norm and SGD was used instead of the SVM due to have a significantly larger AUC (0.79) than the SVM (0.71)

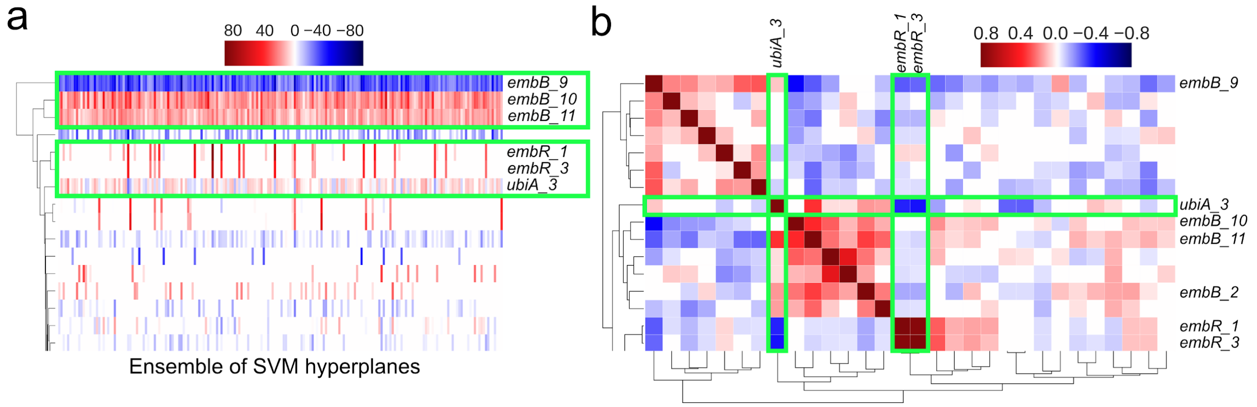

Supplementary Figure 6: Pairwise correlation of ethambutol genetic features across ensemble of SGD-SVM simulations.

(**a**) SVM weightings across the hyperplane ensemble. The x-axis represents the iterations for each unique SVM simulation. The y-axis represents the alleles selected by each SVM. Red corresponds to a positive weighting while blue corresponds to a strong negative weighting. The alleles of *embB*, *ubi*A, and *embR* are highlighted in green. (**b**) Clustering of ethambutol allele correlation matrix. The color blue corresponds to a negative correlation while a blue color corresponds to a positive correlation. The y-axis is shown since the figure since the x-axis is the mirror of the y-axis. The alleles of *embB*, *ubi*A, and *embR* are highlighted in green.

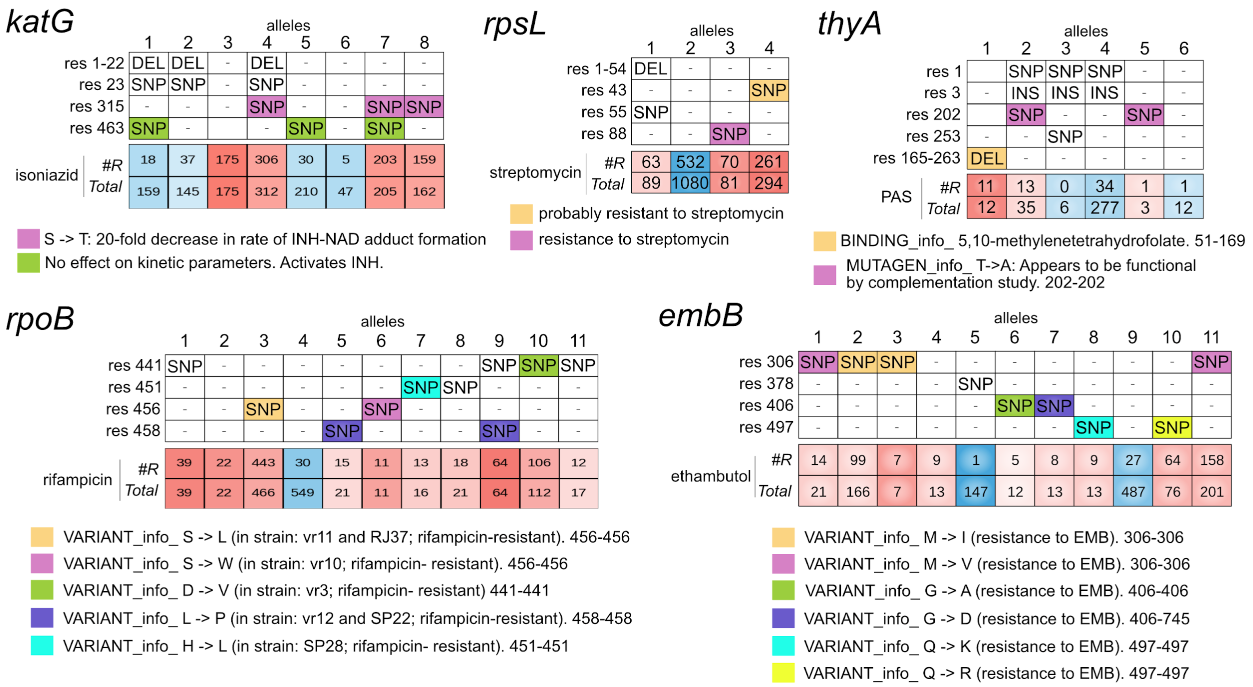

Supplementary Figure 7: Case-controls for relating MoA with uniprot annotated protein structural features. Mutation tables and uniprot color annotations are shown for *katG*, *rpsL*, *thyA*, *rpoB*, and *embB*.

Supplementary Tables

Supplementary Table 1

Virulence Factor Accessory Genes	Gene product annotation
**Rv3478**	PPE family protein
**Rv1818c**	PE-PGRS family protein
**Rv0355c**	PPE family protein
**Rv2123**	PPE family protein
**Rv0304c**	PPE family protein
**Rv1361c**	PPE family protein
**Rv1787**	PPE family protein
**Rv1196**	PPE family protein
**Rv1789**	PPE family protein
**Rv1790**	PPE family protein
**Rv1168c**	PPE family protein
**Rv3343c**	PPE family protein
**Rv1651c**	PE-PGRS family protein
**Rv2396**	PE-PGRS family protein

**Rv3022A**	PE family protein
**Rv1386**	PE family protein
**Rv1788**	PE family protein
**Rv2351C**	Phospholipase C 4 precursor (EC 3.1.4.3)
**Rv2349C**	Phospholipase C 4 precursor (EC 3.1.4.3)
**Rv2350C**	Phospholipase C 4 precursor (EC 3.1.4.3)
**Rv1755c**	Phospholipase C 4 precursor (EC 3.1.4.3)
**Rv3487c**	Esterase/lipase
**Rv3084**	Esterase/lipase
**Rv0982**	Osmosensitive K+ channel histidine kinase KdpD (EC 2.7.3.-)
**Rv0171**	MCE-family protein MceC
**Rv0867c**	Resuscitation-promoting factor RpfA
**Rv2192c**	Anthranilate phosphoribosyltransferase (EC 2.4.2.18)
**Rv1915**	Isocitrate lyase (EC 4.1.3.1)
**Rv1940**	3,4-dihydroxy-2-butanone 4-phosphate synthase (EC 4.1.99.12) / GTP cyclohydrolase II (EC 3.5.4.25)
**Rv3020c**	ESAT-6-like protein EsxG
**Rv3019c**	ESAT-6-like protein EsxH, 10 kDa antigen CFP7

Supplementary Table 1: Pan-genome partitioning of virulence factors.

Supplementary Table 2

**Counteractome categories**	**Counteractome genes**	**Pan-genome partition**
Adenylate Cyclases	Rv0891c	Core
	Rv1359	Accessory
	Rv2435c	Core
	Rv1358	Accessory
	Rv2488c	Core
	Rv0386	Core
	Rv1264	Core
	Rv1625c	Core
	Rv1900c	Core
	Rv2212	Core
	Rv1647	Core
	Rv1318c	Core
	Rv1319c	Accessory
	Rv1320c	Core
	Rv3645	Core
Tryptophan starvation	Rv2192c	Accessory
	Rv2246	Core
	Rv1612	Core
	Rv3160c	Core
	Rv1609	Core
	Rv1053c	Accessory
	Rv3374	Core
	Rv2661c	none
	Rv1559	Core
	Rv1013	Core
	Rv2283	none

	Rv0346c	Core
Nitrosative stress	Rv0757	Core
	Rv3283	Core
	Rv3270	Core
	Rv1620c	Core
	Rv1622c	Core
	Rv2563	Core
	Rv0467	Core
	Rv3855	Core
	Rv0561c	Core
	Rv3200c	Core
	Rv2476c	Core
	Rv2047c	Core
Acid stress	Rv1621c	Core
	Rv1623c	Core
	Rv1622c	Core
	Rv1620c	Core
Acid stress buffer	Rv0536	Core
	Rv2282c	Core
	Rv1339	Core
	Rv2665	Unique
	Rv2943A	Unique
	Rv1717	Accessory
	Rv1620c	Core
	Rv0326	Core
	Rv1621c	Core
	Rv1623c	Core
	Rv2630	Core
	Rv0612	Accessory
	Rv1287	Core
	Rv2758c	Core
	Rv3229c	Core
	Rv3203	Core
	Rv2089c	Core
	Rv3013	Core
	Rv1622c	Core
	Rv1284	Core
	Rv0324	Core

Supplementary Table 2: Pan-genome partitioning of counteractome genes.

Supplementary Table 3

Drug	Threshold	Key clusters filtered
Ethionamide	200	Cluster 2122 (*eth*A)
Pyrazinamide	175	Cluster 3930 (*pnc*A), Cluster 1613 (*pnc*A)
Ethambutol	190	Cluster 551 (*emb*B), Cluster 4244 (*ubi*A)
Isoniazid	200	Cluster 1116 (*kat*G)
Rifampicin	195	Cluster 491 (*rpo*B), Cluster 415 (*rpo*C)
Amikacin	195	-
Ciprofloxacin	195	-
Cycloserine	215	-
Kanamycin	200	-
Moxifloxacin	205	-
Nicotinamide	180	-
Ofloxacin	210	Cluster 893 (*gyr*A)
Capreomycin	190	-

4-aminosalicylic acid	220	Cluster 4486 (*thy*A)
Rifabutin	185	-
Streptomycin	175	Cluster 7435 (*rps*L), Cluster 5240 (*gid*B)

Supplementary Table 3: Scikit-learn SVM thresholds and pan-genome clusters filtered for each drug simulation. The alleles listed as key clusters filtered are those that were only allowed for simulations in the corresponding drug in the row. For example, all alleles within Cluster 2122 (*eth*A) were not accounted for as genetic features in the simulations for all drugs except for ethionamide.

Supplementary Discussion

Characteristics of 1,595 Strain M. *tuberculosis* dataset

The chosen strains come from a wide range of studies [2] [3] [4] [5] [6] [7] [8] [9] [10] [11] [12] [13] [14] [15] [16] [17] [18]. Because Africa exhibits the most diverse set of M. *tuberculosis* strains in the world [19], a third of our strains were isolated in South Africa (**Supplementary Fig. 1a**). Furthermore, the chosen dataset constitutes a wide spectrum of isolation hotspots, ranging from 144 strains in Sweden to 141 strains in Belarus. Notably, 78 strains were isolated from South Korea, a country that has endured a significant increase in M. *tuberculosis* incidence since 2005 [1]. In total, 70% of the selected strains were in "high burden countries" [1].

Characterizing the M. *tuberculosis* pan-genome

Following selection of the representative set of M. *tuberculosis* genome sequences, we determined the pan-genome (i.e., the union of all genes across all strains) represented by these data (**Methods**). We categorized the genome content across all 1,595 strains as "core" (the set of genes shared by at least 1590 strains), "accessory" (the set of genes present in some, but not all, strains), or "unique" (the set of genes found in at most 5 strains) [20] [21]; the cutoffs for each of these categories were evaluated using sensitivity analyses (**Methods**). The resulting pan-genome consisted of 11,039 clusters, where each cluster represents a grouping of protein variants determined to be sufficiently similar to each other (i.e., >80% sequence similarity). Using these partitioning criteria, the core, accessory, and unique genomes were composed of 3,419 genes (31%), 2,402 genes (21.8%), and 5,218 genes (47.3%), respectively (**Supplementary Fig. 2a**). The core genome made up 80% of the average genome in our dataset, a result in agreement with the hypothesis that M. *tuberculosis* is a clonal species [22]. This diversity is in stark contrast to that of *Escherichia coli*, which has a core genome percentage estimated to be between 20% and 50% of the average full E. *coli* genome [23], and *Staphylococcus aureus*, where we recently calculated the core genome to comprise 56% of the average genome [21]. Furthermore, we found that virulence factors were highly conserved in the M. *tuberculosis* core genome (93%, 414/445 genes) (**Supplementary Table 1** and **Supplementary Discussion**).

The remaining 7,620 genes that comprise the accessory and unique genomes represent the genetic variability across M. *tuberculosis* strains. A significant portion of the unique and accessory genome was attributed to Pro-Glu (PE)-related proteins and hypothetical proteins (**Supplementary Fig. 2b**).

Specifically, PE-related proteins represent products that contain the characteristic motifs Pro-Glu (PE), Pro-Pro-Glu (PPE), or polymorphic GC-rich sequence motifs (PE-PGRS) [24] and make up approximately 10% of the average *M. tuberculosis* coding capacity [25]. Because of significant variation in both PE-related proteins and hypothetical proteins, we computed the shape of the pan-genome by filtering out PE/PPE genes and genes with lengths that were significantly longer (>1 standard deviation) than the mean gene length of 1000 bp, which are likely result of sequencing or annotation errors. In total, this led to the removal of 1,335 genes clusters from the pan-genome. The majority of these genes (826) were PE/PPE genes. Following the removal of these genes we find that the pan-genome is closed for our 1595 strains of *M. tuberculosis* (**Supplementary Fig. 2c**).

Pan-genome COG Categories

We used eggNog with the eggNog-mapper tool [26] to functionally categorize the pan-genome into Clusters of Orthologous Groups (COGs) [27] (**Supplementary Fig. 3f**). We filtered out clusters annotated as PE genes or those marked as hypothetical proteins in order to focus on the functionally characterized pan-genome. The core genome made up less than 50% of the clusters annotated with defense mechanisms (V), signal transduction mechanisms (T), inorganic ion transport and metabolism (P), and secondary metabolism (Q) COGs. In contrast, the core made up more than 70% of clusters annotated with intracellular trafficking, secretion, and vesicular transport (U), and translation, ribosomal structure and biogenesis (J).

Virulence factors are highly conserved in the core genome

The pathogenicity of *M. tuberculosis* can be partly attributed to its unique set of virulence factors, whose variable distribution may provide further insight into pathogenic requirements. Thus, we determined the distribution of 445 virulence factors, curated by the PATRIC database [28], across the constructed pan-genome. Of the 445 virulence factors, 7.0% (31 genes) were in the accessory genome and 93.0% (414 genes) were in the core genome **(Supplementary Table 1)**. Of the 31 accessory virulence genes, 17 were PPE/PE/PGRS genes (**Supplementary Table 1**). Also partitioned in the accessory genome was a set of six virulence factors composed of genes encoding the phospholipases C (*plcC*, *plcD*, *plcA*, and *plcB*) [29], and *lipR* (a lipolytic esterase). The remaining eight virulence factors found in the accessory genome were *kdpD*, *mceC*, *rpfA*, *trpD*, *aceAa*, *ribA1*, Rv0969, and *ctpV*, and two ESAT-6 like proteins, *esxG* and *esxH*. *esxG* and *esxH* comprise part of the ESX-3 secretion system involved in mycobactin-mediated iron acquisition but may play an additional role in virulence [30]. The isocitrate lyase subunit (*aceAa*) is a nonessential gene within the glyoxylate shunt and is downregulated in antibiotic conditions [31].

In addition to virulence factors, we investigated the "CD4 counteractome"—defined as the specific set of genes necessary for coping with the immune environment generated by CD4 T cells [32]. We found that all of the genes were partitioned in the core genome with the exception of a *trpD*, *Rv1053*, and three adenylate cyclases (*Rv1358*, *Rv1359*, and *Rv1319c*) (**Supplementary Table 2**). Interestingly, the existence of an alternative tryptophan biosynthesis pathway suggested by [3334] is supported by the partitioning of *trpD* in the accessory genome.

Among the accessory genes found in the virulome and counteractome, *trpD* (anthranilate phosphoribosyltransferase) stood out as it is an essential tryptophan biosynthesis gene. Interestingly, in a study comparing *trpE* and *trpD* deleted strains, it was found that the *trpE* deleted strains had a 100,000 fold loss of viability after 2 weeks in contrast to the *trpD* deleted ones which could not achieve such a level after 13 weeks[33,34]. *Zang et al.* hypothesized that such a difference could either be due to either "an accumulation of intermediary metabolites or an as of yet undescribed alternative tryptophan biosynthesis pathway"[34]. In our case, the partitioning of *trpD* to the accessory genome could either be due to the absence of *trpD* in 1000+ strains or due to *trpD* having significant sequence variability. A quick check on the PATRIC database corroborates our findings in that many strains lack an annotated *trpD*. Given the drastic experimental differences between *trpD* and *trpE* deletions and the rare occurrence of accessory virulence factors, we believe that the significant absence of *trpD* in the constructed pan-genome supports the claim that there is an alternative Tryptophan biosynthesis pathway in M. *tuberculosis*.

Motivation for using mutual information and observation of shared AMR signals across multiple antibiotics

In our study, mutual information (MI) was used to quantify the dependence between the labeled phenotype distribution of a specific drug (resistant or susceptible) and the distribution of a specific variant (presence or absence), across all tested strains (**Supplementary Figure 3g**). MI was chosen due to having many statistical benefits, which include being a nonparametric method that can quantify nonlinear relationships unlike Pearson's correlation which measures a linear relationship. MI has proven to be a natural and powerful means to equitably quantify statistical associations in large datasets [35]. In addition to key AMR genes (**Fig. 1**), mutual information picks up a other known resistance-conferring genes including *eth*A (Rv3854) [36], *pap*A2 (Rv1182) [37], *drr*A (Rv2936) [38], *drrB* (Rv2937) [38], *gidB* (Rv3919c) [39], *moe*W (Rv2338c) [40] and *ubi*A (Rv3806c) [41,42] **(Supplementary Data 1)**.

MI showed that the variants associated with the highest signals are often those representative of susceptible rather than resistant phenotypes, thus indicating that knowledge of the presence of a susceptible variants in M. *tuberculosis* holds more informational value in determining the AMR phenotype.

It is important to note that M. *tuberculosis* treatment consists of the combined use of multiple drugs, which in turn make many M. *tuberculosis* strains (reflected in their genomes) resistant to multiple antibiotics. Therefore, it comes as no surprise that key resistance-determining genes showed up as tall peaks with other drugs (**Fig. 1**). These multi-antibiotic resistant M. *tuberculosis* strains make relating a specific variant to a AMR challenging [43,44].

Motivation of ensemble support vector machine and limitations

Although simple and effective, mutual information does not account for the relationship between interacting alleles since the pairwise calculations consider variants independently of one another. In order to uncover possible structures in our dataset related to AMR, we used a Support Vector

Machine (SVM) to select AMR-associated alleles. We introduced both unstable and randomized behavior in the SVM by using an L1-norm penalty and stochastic gradient descent. A “noisier” SVM was used in order to address the following two inherent biases in the AMR data: (1) that the binary AMR phenotype (resistant or susceptible) is biased towards *in vitro* drug testing conditions, and (2) that the binary AMR phenotype does not account for varying levels of drug efficacy which may determine high level resistance. We looked at an ensemble of noisy SVM simulations for each drug in order to get a notion of significance (genes that pop out in many simulations are more likely to be significant) (**Methods**).

The unstable and randomized SVM method may slightly relieve the bias introduced by the AMR phenotypes (resistant or susceptible) experimentally determined from *in vitro* testing conditions. As noted earlier, the host environment of M. *tuberculosis* is drastically different from the one encountered in the petri dish, and such differences influence the efficacy of drugs [45]. Moreover, the AMR phenotype is binary and does not consider variation in the drug concentration profiles. Therefore, “explaining resistance” by finding a minimal set of mutations that best explains the *in vitro* AMR phenotypes may not capture subtle genetic adaptations. Other possible influential adaptations, however, such as those under the complex resistance category that have been shown to result in varying levels of resistance [41], may be hidden within the genomic data. Thus, this “loose” machine learning method extracts features from suboptimal peaks as well as from areas surrounding the global optima. Furthermore, it is important to note that current treatments of M. *tuberculosis* infection consists of the combined use of multiple drugs, which in turn make many M. *tuberculosis* strains resistant to multiple antibiotics.

A key biomarker that was not uncovered was the streptomycin AMR-determinant, *rrs*, because only protein coding genes were taken into account in our analysis. We find many cell wall genes implicated in the analysis as well including *pks12* [46], *pks9*, *pks2*, *dprE1*, *pks7*, *pks1* , *pks6*, *ltp1*, and *ddpX*. Furthermore, many implicated alleles occur in sulfur metabolism including *cysK2*, *serA1*, *moaE2*, *mec*, and *metZ*. The presence of *cydC* as an implicated gene was interesting because studies have shown that it is important for host immune response and that disruptions in *cydC* affect antibiotic efficacy [47,48].

Defining SNPs is not required for identification of AMR genes

Defining SNPs relative to the M. *tuberculosis* H37Rv reference strain has provided the foundation both for diagnostics and for identifying novel resistance-conferring mutations but has limited a comprehensive and unbiased analysis of the M. *tuberculosis* AMR mutational landscape [44,49,50] [51]. Our representation of genetic variation and subsequent identification of key AMR genes demonstrates that reference-based genetic variation is not required for comprehensively identifying AMR genes. Rather, by representing genetic features as exact allele sequences, each strain in our dataset contains a single genetic feature for each of its genes, which removes potential confounding effects that may arise when multiple genetic features appear in a single gene.

Limitations of our view of genetic variation

The primary limitation in our view of genetic variation is that we do not account for non-protein coding genes. Therefore, our analysis is unable to identify known non-protein coding genes that

confer resistance such as *eis* and *rrs*. Furthermore, by only looking at protein sequences, we do not account for synonymous SNPs, which have been shown to confer resistance [41]. While we focused our view on protein-coding genes and their protein sequences, there is no limitation in the ability of our computational platform to account for non-protein coding genes and synonymous SNPs.

Machine learning enables increased identification of known AMR genes over GWAS

Our results suggest that a machine learning approach that accounts for multi-dimensional correlations is more powerful than a typical GWAS-based approach that tests positions on the genome individually for association with a phenotype [52]. Implementing an ensemble SVM identified 33 known AMR genes, including an additional 7 gene-to-antibiotic relations absent from our lists derived from pairwise statistical associations. Our observation of significant correlations between *embB*, *ubiA*, and *embR* implied that machine learning may provide a base for the quantitative analysis of epistatic interactions. In particular, our pipeline identified an optimal mapping between multiple genetic features and AMR phenotypes. This mapping elucidates complex relations underlying AMR evolution that are hidden from simple GWAS analysis. While we utilized an SVM for its clarity, future efforts may implement machine learning methods capable of capturing more complexity, or integrate phylogenetic constraints in the optimization problem.

Adaptations in toxins are associated with XDR in *M. tuberculosis*

In addition to analyzing the resistance to individual antibiotics, we looked at AMR genes predicted to contribute to MDR (multidrug-resistant, AUC: 0.96) and XDR (extensively drug-resistant, AUC: 0.92) strains of *M. tuberculosis*. In XDR cases, *mazF3* (Rv1102c) appeared as the top 5th allele and *vapC21* (Rv2757c) appeared as the 10th ranked allele, both of which ranked higher than alleles of known AMR determinants such as *gyrA*, *embB*, *ethA*, *katG*, *thyA*, *ppsA*, and *pncA* (**Supplementary Data 2**). Notably, mRNA levels of *mazF3* have been shown to be induced 6.0-, 8.9-, and 8-fold by isoniazid, gentamycin, and rifampicin, respectively, when grown in a non-replicating, starved state [53]. The hyperplane weights for *mazF3* and *vapC21* showed that *mazF3* allele 6 and *vapC21* allele 7 were selected as determinants for resistance and susceptibility, respectively. In addition to the mentioned XDR-associated toxins, other implicated AMR toxins that appeared across the antibiotics include *mazF5* (8th rank, PAS), *higA* (30th rank, PAS), *vapC2* (21th rank, ETH), *higB* (49th rank, EMB). In particular, *mazF5* is part of a toxin-antitoxin module (*mazF5*-*mazE5*) that has been shown to be in the top five most differentially expressed genes in a XDR M. *tuberculosis* strain [54]. The uncovering of toxins by our machine learning approach complements and extends recent experimental studies by relating toxin variation to host-relevant AMR evolution.

Epistatic and protein-structure-guided generation of experimental hypothesis

Extending our sequence-based view of these implicated AMR genes by mapping alleles to protein structures provides a basis for inferring the causal driver of adaptation. We found that the two resistant-dominant alleles of *oxcA* uniquely share a SNP A253S located within the thiamin diphosphate-dependent enzyme M-terminal domain, which led us to hypothesize that the SNP A253S promotes acid stress resistance through increased enzyme efficiency. Observation that *oxcA* SNP A253S occurs in the background of *katG* S315T suggests the use of acidic stress and M. *tuberculosis* strains carrying the S315T harbinger mutation [14] in experimental interrogation of *oxcA* in high-level isoniazid resistance.

Given the difficulty of experimenting with M. *tuberculosis*—where slow growth rate, host-irrelevant media conditions, and biosafety level 3 requirements burden experimentalists—our results demonstrate that an additional interpretation of computationally-derived mutations by analyzing protein structures may accelerate experimental investigation of this deadly pathogen. Beyond mutation proximality and feature incidence, future efforts may better utilize protein structures by estimating changes in biochemical properties due to mutations, such as changes in metabolite or cofactor binding affinities [55].

Geographic contextualization suggests modulation of antibiotic treatment

Our geographic contextualization of the implicated AMR genes identifies novel genetic adaptations specific to Belarus—a country that had the highest rate of MDR M. *tuberculosis* strains in the world between 2015-2016 [1]. While studies have described the genomic composition of Belarus strains in terms of the commonly used AMR genes [56], our identification of resistant-dominant alleles within *Rv*3848, *oxc*A, *kdp*C, *dna*A, and *vap*C21 demonstrates that the focused view of genetic variation is limiting. Modulation of treatment regimens may reflect these genetic adaptations by removing isoniazid, streptomycin, and ethambutol. Furthermore, observation that susceptible dominant alleles of *thy*A, *mmpL11*, and *ald* are localized in Belarus suggests that a combinatorial antibiotic regimen based on PAS and d-cycloserine may increase the likelihood of effective MDR M. *tuberculosis* treatment. We believe that additional epidemiological perspectives should enable actionable insight to the problem of poor M. *tuberculosis* management.

References

1. Organization, W. H. & Others. Global tuberculosis report 2016. (2016).
2. Miyoshi-Akiyama, T., Matsumura, K., Iwai, H., Funatogawa, K. & Kirikae, T. Complete annotated genome sequence of Mycobacterium tuberculosis Erdman. *J. Bacteriol.* **194,** 2770 (2012).
3. Roetzer, A. *et al.* Whole genome sequencing versus traditional genotyping for investigation of a Mycobacterium tuberculosis outbreak: a longitudinal molecular epidemiological study. *PLoS Med.* **10,** e1001387 (2013).
4. Wu, W. *et al.* A genome-wide analysis of multidrug-resistant and extensively drug-resistant strains of Mycobacterium tuberculosis Beijing genotype. *Mol. Genet.*

Genomics **288,** 425–436 (2013).

5. Majid, M. *et al.* Genomes of Two Clinical Isolates of Mycobacterium tuberculosis from Odisha, India. *Genome Announc.* **2,** (2014).
6. Ng, K. P. *et al.* Draft Genome Sequence of the First Isolate of Extensively Drug-Resistant (XDR) Mycobacterium tuberculosis in Malaysia. *Genome Announc.* **1,** (2013).
7. Lin, N., Liu, Z., Zhou, J., Wang, S. & Fleming, J. Draft genome sequences of two super-extensively drug-resistant isolates of Mycobacterium tuberculosis from China. *FEMS Microbiol. Lett.* **347,** 93–96 (2013).
8. Lanzas, F., Karakousis, P. C., Sacchettini, J. C. & Ioerger, T. R. Multidrug-resistant tuberculosis in panama is driven by clonal expansion of a multidrug-resistant Mycobacterium tuberculosis strain related to the KZN extensively drug-resistant M. tuberculosis strain from South Africa. *J. Clin. Microbiol.* **51,** 3277–3285 (2013).
9. Cohen, K. A. *et al.* Evolution of Extensively Drug-Resistant Tuberculosis over Four Decades: Whole Genome Sequencing and Dating Analysis of Mycobacterium tuberculosis Isolates from KwaZulu-Natal. *PLoS Med.* **12,** e1001880 (2015).
10. Ismail, A. *et al.* Draft Genome Sequence of a Clinical Isolate of Mycobacterium tuberculosis Strain PR05. *Genome Announc.* **1,** (2013).
11. Karuthedath Vellarikkal, S. *et al.* Draft Genome Sequence of a Clinical Isolate of Multidrug-Resistant Mycobacterium tuberculosis East African Indian Strain OSDD271. *Genome Announc.* **1,** (2013).
12. Al Rashdi, A. S. A., Jadhav, B. L., Deshpande, T. & Deshpande, U. Whole-Genome Sequencing and Annotation of a Clinical Isolate of Mycobacterium tuberculosis from Mumbai, India. *Genome Announc.* **2,** (2014).
13. Winglee, K. *et al.* Whole Genome Sequencing of Mycobacterium africanum Strains

from Mali Provides Insights into the Mechanisms of Geographic Restriction. *PLoS Negl. Trop. Dis.* **10,** e0004332 (2016).

14. Manson, A. L. *et al.* Genomic analysis of globally diverse Mycobacterium tuberculosis strains provides insights into the emergence and spread of multidrug resistance. *Nat. Genet.* **49,** 395–402 (2017).
15. Merker, M. *et al.* Evolutionary history and global spread of the Mycobacterium tuberculosis Beijing lineage. *Nat. Genet.* **47,** 242–249 (2015).
16. Isaza, J. P. *et al.* Whole genome shotgun sequencing of one Colombian clinical isolate of Mycobacterium tuberculosis reveals DosR regulon gene deletions. *FEMS Microbiol. Lett.* **330,** 113–120 (2012).
17. Cole, S. T. *et al.* Deciphering the biology of Mycobacterium tuberculosis from the complete genome sequence. *Nature* **393,** 537–544 (1998).
18. Camus, J.-C., Pryor, M. J., Médigue, C. & Cole, S. T. Re-annotation of the genome sequence of Mycobacterium tuberculosis H37Rv. *Microbiology* **148,** 2967–2973 (2002).
19. Gagneux, S. & Small, P. M. Global phylogeography of Mycobacterium tuberculosis and implications for tuberculosis product development. *Lancet Infect. Dis.* **7,** 328–337 (2007).
20. Medini, D., Donati, C., Tettelin, H., Masignani, V. & Rappuoli, R. The microbial pan-genome. *Curr. Opin. Genet. Dev.* **15,** 589–594 (2005).
21. Bosi, E. *et al.* Comparative genome-scale modelling of Staphylococcus aureus strains identifies strain-specific metabolic capabilities linked to pathogenicity. *Proc. Natl. Acad. Sci. U. S. A.* **113,** E3801–9 (2016).
22. Supply, P. *et al.* Linkage disequilibrium between minisatellite loci supports clonal evolution of Mycobacterium tuberculosis in a high tuberculosis incidence area. *Mol.*

Microbiol. **47,** 529–538 (2003).

23. Lukjancenko, O., Wassenaar, T. M. & Ussery, D. W. Comparison of 61 sequenced Escherichia coli genomes. *Microb. Ecol.* **60,** 708–720 (2010).
24. Bottai, D. & Brosch, R. Mycobacterial PE, PPE and ESX clusters: novel insights into the secretion of these most unusual protein families. *Mol. Microbiol.* **73,** 325–328 (2009).
25. Glickman, M. S. & Jacobs, W. R., Jr. Microbial pathogenesis of Mycobacterium tuberculosis: dawn of a discipline. *Cell* **104,** 477–485 (2001).
26. Huerta-Cepas, J. *et al.* Fast genome-wide functional annotation through orthology assignment by eggNOG-mapper. *bioRxiv* 076331 (2016). doi:10.1101/076331
27. Tatusov, R. L., Galperin, M. Y., Natale, D. A. & Koonin, E. V. The COG database: a tool for genome-scale analysis of protein functions and evolution. *Nucleic Acids Res.* **28,** 33–36 (2000).
28. Mao, C. *et al.* Curation, integration and visualization of bacterial virulence factors in PATRIC. *Bioinformatics* **31,** 252–258 (2015).
29. Raynaud, C. *et al.* Phospholipases C are involved in the virulence of Mycobacterium tuberculosis. *Mol. Microbiol.* **45,** 203–217 (2002).
30. Tufariello, J. M. *et al.* Separable roles for Mycobacterium tuberculosis ESX-3 effectors in iron acquisition and virulence. *Proc. Natl. Acad. Sci. U. S. A.* **113,** E348–57 (2016).
31. Nandakumar, M., Nathan, C. & Rhee, K. Y. Isocitrate lyase mediates broad antibiotic tolerance in Mycobacterium tuberculosis. *Nat. Commun.* **5,** 4306 (2014).
32. Russell, D. G. Trp'ing tuberculosis. *Cell* **155,** 1209–1210 (2013).
33. Parish, T. Starvation survival response of Mycobacterium tuberculosis. *J. Bacteriol.* **185,** 6702–6706 (2003).
34. Zhang, Y. J. *et al.* Tryptophan biosynthesis protects mycobacteria from CD4 T-cell-

mediated killing. *Cell* **155,** 1296–1308 (2013).

35. Kinney, J. B. & Atwal, G. S. Equitability, mutual information, and the maximal information coefficient. *Proc. Natl. Acad. Sci. U. S. A.* **111,** 3354–3359 (2014).

36. Morlock, G. P., Metchock, B., Sikes, D., Crawford, J. T. & Cooksey, R. C. ethA, inhA, and katG loci of ethionamide-resistant clinical Mycobacterium tuberculosis isolates. *Antimicrob. Agents Chemother.* **47,** 3799–3805 (2003).

37. Danilchanka, O., Mailaender, C. & Niederweis, M. Identification of a novel multidrug efflux pump of Mycobacterium tuberculosis. *Antimicrob. Agents Chemother.* **52,** 2503–2511 (2008).

38. Li, G. *et al.* Study of efflux pump gene expression in rifampicin-monoresistant Mycobacterium tuberculosis clinical isolates. *J. Antibiot.* **68,** 431–435 (2015).

39. Wong, S. Y. *et al.* Mutations in gidB confer low-level streptomycin resistance in Mycobacterium tuberculosis. *Antimicrob. Agents Chemother.* **55,** 2515–2522 (2011).

40. Wang, F. *et al.* Identification of a small molecule with activity against drug-resistant and persistent tuberculosis. *Proc. Natl. Acad. Sci. U. S. A.* **110,** E2510–7 (2013).

41. Safi, H. *et al.* Evolution of high-level ethambutol-resistant tuberculosis through interacting mutations in decaprenylphosphoryl-[beta]-D-arabinose biosynthetic and utilization pathway genes. *Nat. Genet.* **45,** 1190–1197 (2013).

42. Lingaraju, S. *et al.* Geographic Differences in the Contribution of ubiA Mutations to High-Level Ethambutol Resistance in Mycobacterium tuberculosis. *Antimicrob. Agents Chemother.* **60,** 4101–4105 (2016).

43. Davis, J. J. *et al.* Antimicrobial Resistance Prediction in PATRIC and RAST. *Sci. Rep.* **6,** 27930 (2016).

44. Desjardins, C. A. *et al.* Genomic and functional analyses of Mycobacterium tuberculosis

strains implicate ald in D-cycloserine resistance. *Nat. Genet.* **48,** 544–551 (2016).

45. Sakoulas, G. *et al.* Nafcillin enhances innate immune-mediated killing of methicillin-resistant Staphylococcus aureus. *J. Mol. Med.* **92,** 139–149 (2014).
46. Philalay, J. S., Palermo, C. O., Hauge, K. A., Rustad, T. R. & Cangelosi, G. A. Genes required for intrinsic multidrug resistance in Mycobacterium avium. *Antimicrob. Agents Chemother.* **48,** 3412–3418 (2004).
47. Shi, L. *et al.* Changes in energy metabolism of Mycobacterium tuberculosis in mouse lung and under in vitro conditions affecting aerobic respiration. *Proc. Natl. Acad. Sci. U. S. A.* **102,** 15629–15634 (2005).
48. Dhar, N. & McKinney, J. D. Mycobacterium tuberculosis persistence mutants identified by screening in isoniazid-treated mice. *Proc. Natl. Acad. Sci. U. S. A.* **107,** 12275–12280 (2010).
49. Moradigaravand, D. *et al.* dfrA thyA Double Deletion in para-Aminosalicylic Acid-Resistant Mycobacterium tuberculosis Beijing Strains. *Antimicrob. Agents Chemother.* **60,** 3864–3867 (2016).
50. Martinez, E., Holmes, N., Jelfs, P. & Sintchenko, V. Genome sequencing reveals novel deletions associated with secondary resistance to pyrazinamide in MDR Mycobacterium tuberculosis. *J. Antimicrob. Chemother.* **70,** 2511–2514 (2015).
51. Pearson, T. *et al.* Phylogenetic discovery bias in Bacillus anthracis using single-nucleotide polymorphisms from whole-genome sequencing. *Proc. Natl. Acad. Sci. U. S. A.* **101,** 13536–13541 (2004).
52. Mieth, B. *et al.* Combining Multiple Hypothesis Testing with Machine Learning Increases the Statistical Power of Genome-wide Association Studies. *Sci. Rep.* **6,** 36671 (2016).

53. Tiwari, P. *et al.* MazF ribonucleases promote *Mycobacterium tuberculosis* drug tolerance and virulence in guinea pigs. *Nat. Commun.* **6,** 6059 (2015).

54. de Welzen, L. *et al.* Whole transcriptome and genomic analysis of extensively drug-resistant Mycobacterium tuberculosis clinical isolates identifies downregulation of ethA as a mechanism of ethionamide resistance. *Antimicrob. Agents Chemother.* (2017). doi:10.1128/AAC.01461-17

55. Mih, N., Brunk, E., Bordbar, A. & Palsson, B. O. A Multi-scale Computational Platform to Mechanistically Assess the Effect of Genetic Variation on Drug Responses in Human Erythrocyte Metabolism. *PLoS Comput. Biol.* **12,** e1005039 (2016).

56. Wollenberg, K. R. *et al.* Whole-Genome Sequencing of Mycobacterium tuberculosis Provides Insight into the Evolution and Genetic Composition of Drug-Resistant Tuberculosis in Belarus. *J. Clin. Microbiol.* **55,** 457–469 (2017).
