# Supplementary material for: Machine learning and structural analysis of Mycobacterium tuberculosis pan-genome identifies genetic signatures of antibiotic resistance
Source: Nat Commun. 2018 Oct 17;9:4306. doi: 10.1038/s41467-018-06634-y (PMC6193043; doi:10.1038/s41467-018-06634-y)
Supplement: Supplementary file 3 — Description of Additional Supplementary files [file 41467_2018_6634_MOESM3_ESM.pdf]

- Legends for the Supplementary Data Files:

Supplementary Data 1
Microsoft excel file containing top 40 genetic features associated with each antibiotic determined by mutual information, chi-squared, and ANOVA F-test. ***Sheets 2-14*** corresponds to the following 12 antibiotics: isoniazid, ethambutol, rifampicin, pyrazinamide, streptomycin, ethionamide, ofloxacin, 4-aminosalicylic acid, amikacin, cycloserine, kanamycin, capreomycin.

Supplementary Data 2
Microsoft excel file containing identified genetic features derived by the ensemble support vector machine learning approach. ***Sheets 2-11***: Rank ordering of top SVM feature selected alleles per AMR class based on the absolute sum of the allele weighting across the ensemble of SVM hyperplanes. The 10 AMR classes were chosen due to having an ensemble AUC average greater than 0.80. ***Sheet 12***: Ranking of top 273 SVM genes with the most hits (not weights) across 13 antibiotics. Drugs were weighted according to the number of tested strains (i.e., isoniazid rank 1 hits had more significance than moxifloxacin rank 1).

Supplementary Data 3
Zipped file of 20 png images describing SVM hyperplane ensembles for the 10 AMR classes. 10 of the png images correspond to the hyperplane ensemble for each antibiotic. The other 10 correspond to a correlation matrix of the hyperplane ensemble for each antibiotic.

Supplementary Data 4
Excel spreadsheet describing significant genetic interactions identified by logistic regression models for 10 antibiotic classifications.

Supplementary Data 5
Zipped file of 10 pdf documents portraying allele co-occurrence tables of all gene-gene interactions, categorized by AMR classification. Description of table details are provided in the Figure 2.

Supplementary Data 6
Excel table containing allele sequences, alignment, and protein structure information for the identified AMR genes.

Supplementary Data 7
Excel table containing publication references, sequencing details, accession numbers, and other metadata for the 1,595 *M. tuberculosis* genomes used in this study.

Supplementary Data 8
Excel table containing the lineage distribution of each allele-allele pair contained in the identified gene-gene epistatic interactions.
