# Supplementary figures and images for: Machine learning and structural analysis of Mycobacterium tuberculosis pan-genome identifies genetic signatures of antibiotic resistance
Source: Nat Commun. 2018 Oct 17;9:4306. doi: 10.1038/s41467-018-06634-y (PMC6193043; doi:10.1038/s41467-018-06634-y)

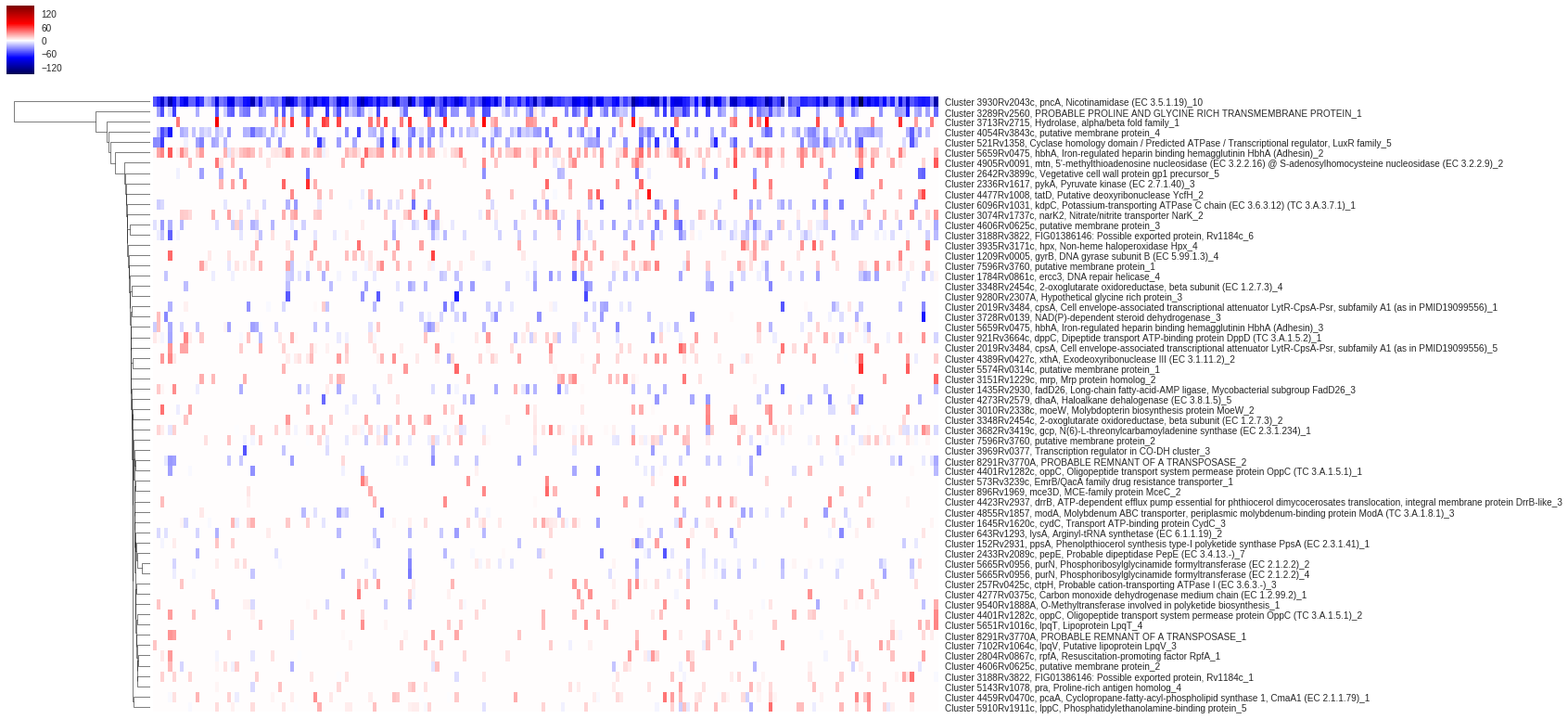

Supplement: Supplementary file 6 — Supplementary Data 3 [file 41467_2018_6634_MOESM6_ESM.zip › Supplementary Data 3/pyrazinamide_SVM_SGD_iterations.png]

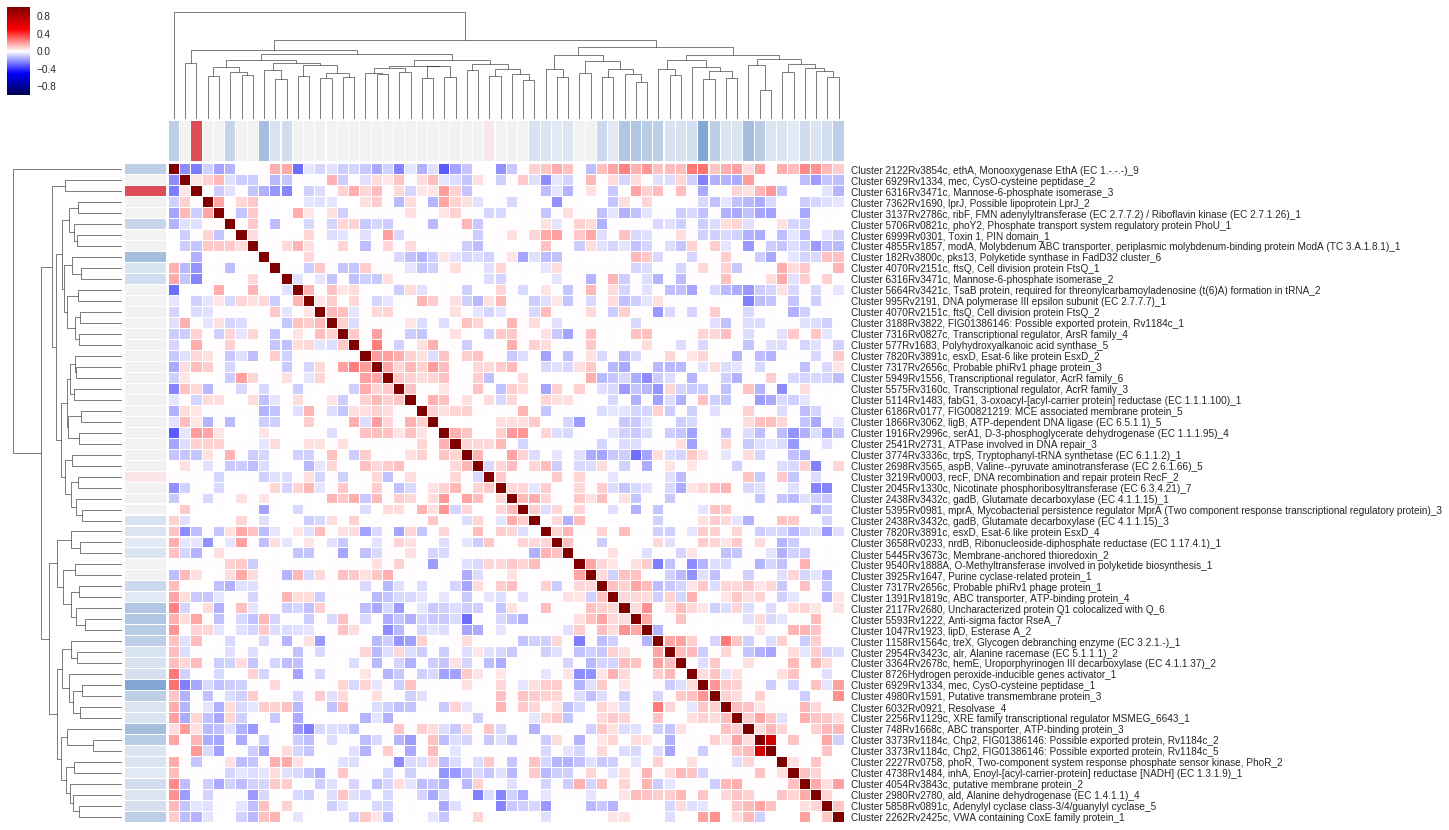

Supplement: Supplementary file 6 — Supplementary Data 3 [file 41467_2018_6634_MOESM6_ESM.zip › Supplementary Data 3/ethionamide_SVM_SGD_correlation.png]

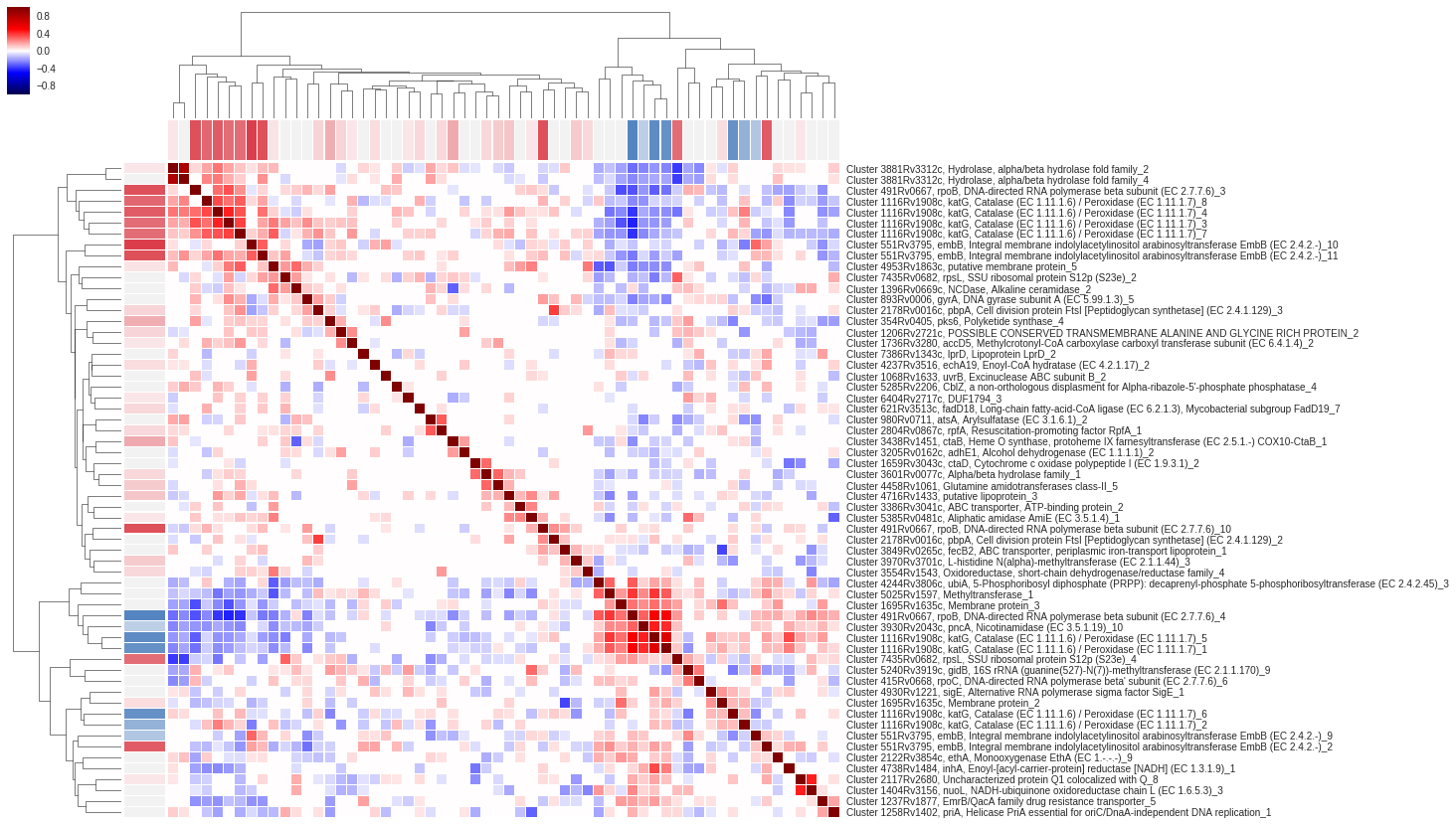

Supplement: Supplementary file 6 — Supplementary Data 3 [file 41467_2018_6634_MOESM6_ESM.zip › Supplementary Data 3/MDR_SVM_SGD_correlation.png]

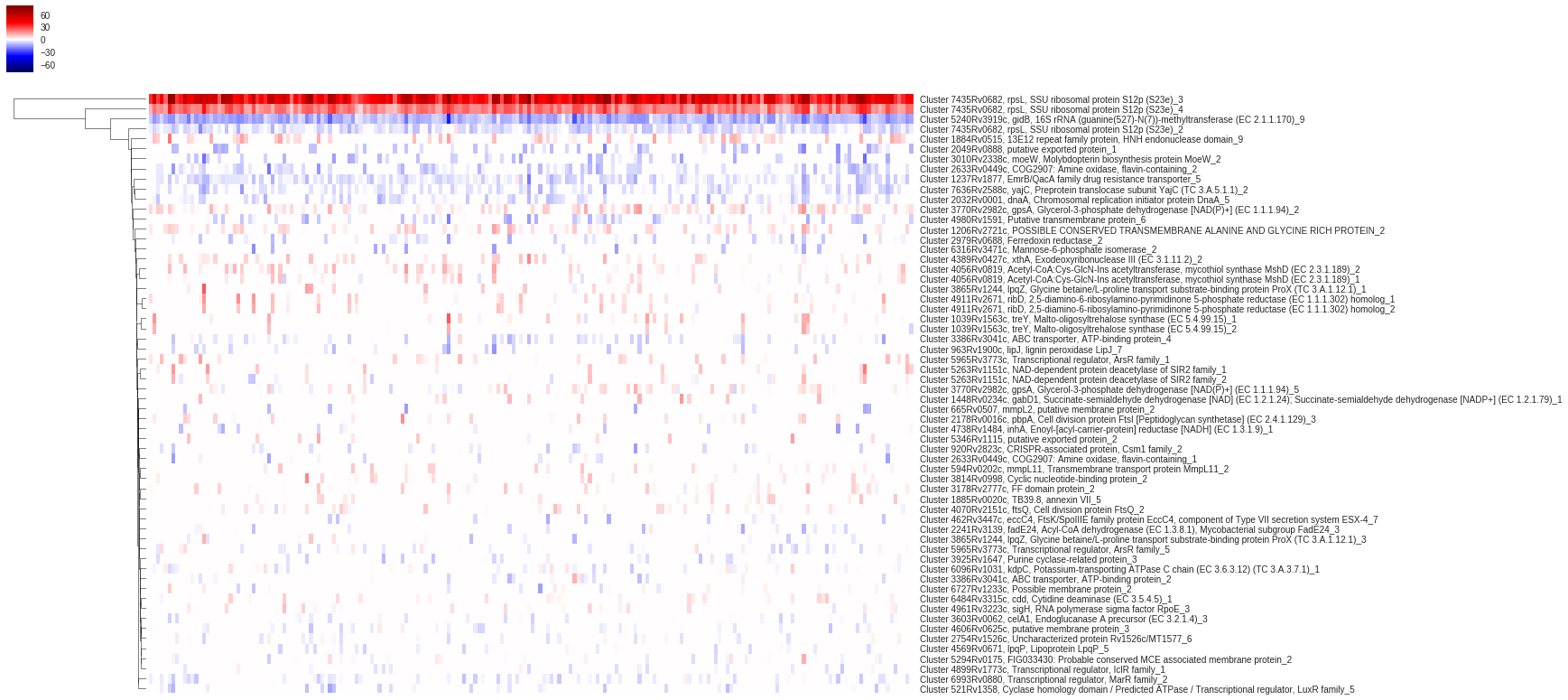

Supplement: Supplementary file 6 — Supplementary Data 3 [file 41467_2018_6634_MOESM6_ESM.zip › Supplementary Data 3/streptomycin_SVM_SGD_iterations.png]

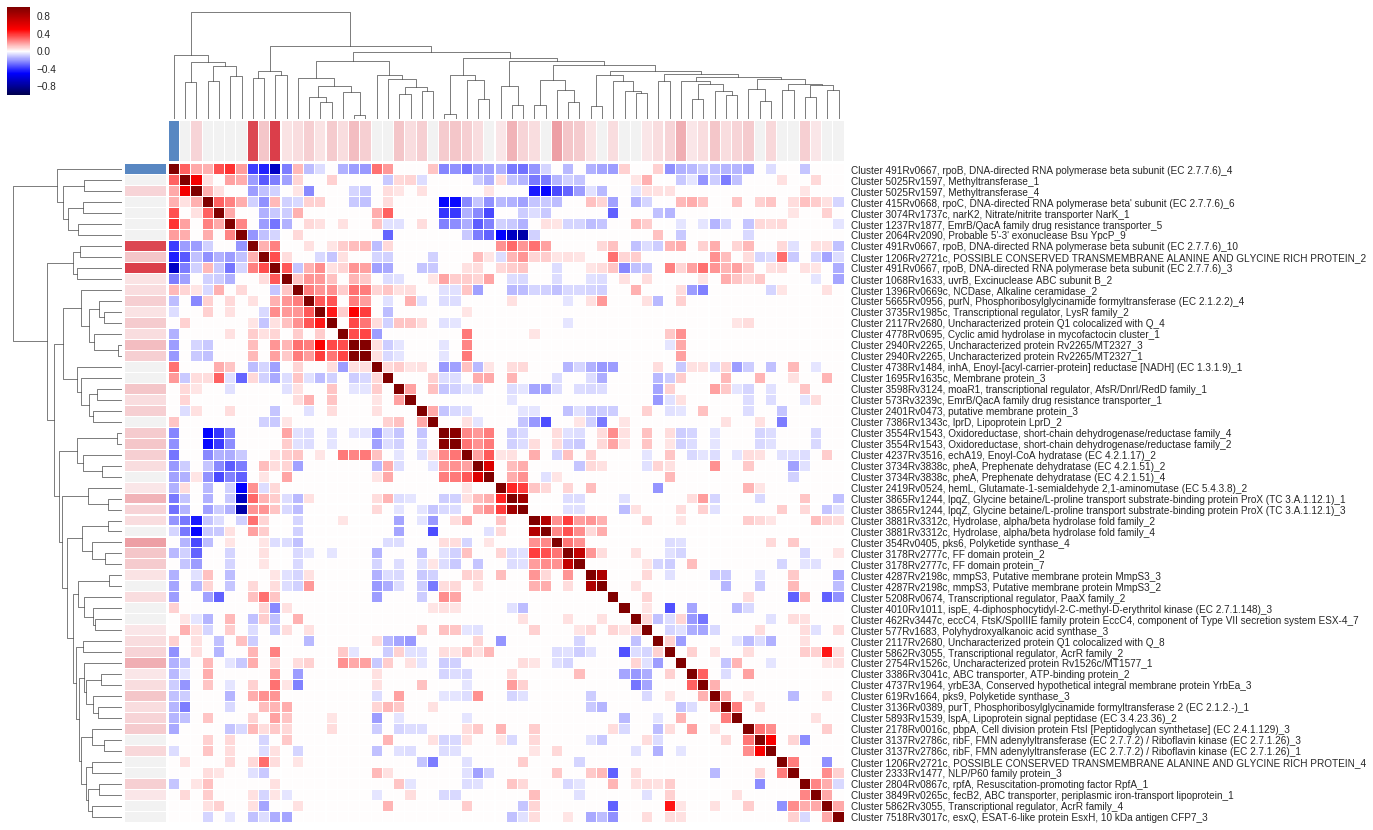

Supplement: Supplementary file 6 — Supplementary Data 3 [file 41467_2018_6634_MOESM6_ESM.zip › Supplementary Data 3/rifampicin_SVM_SGD_correlation.png]

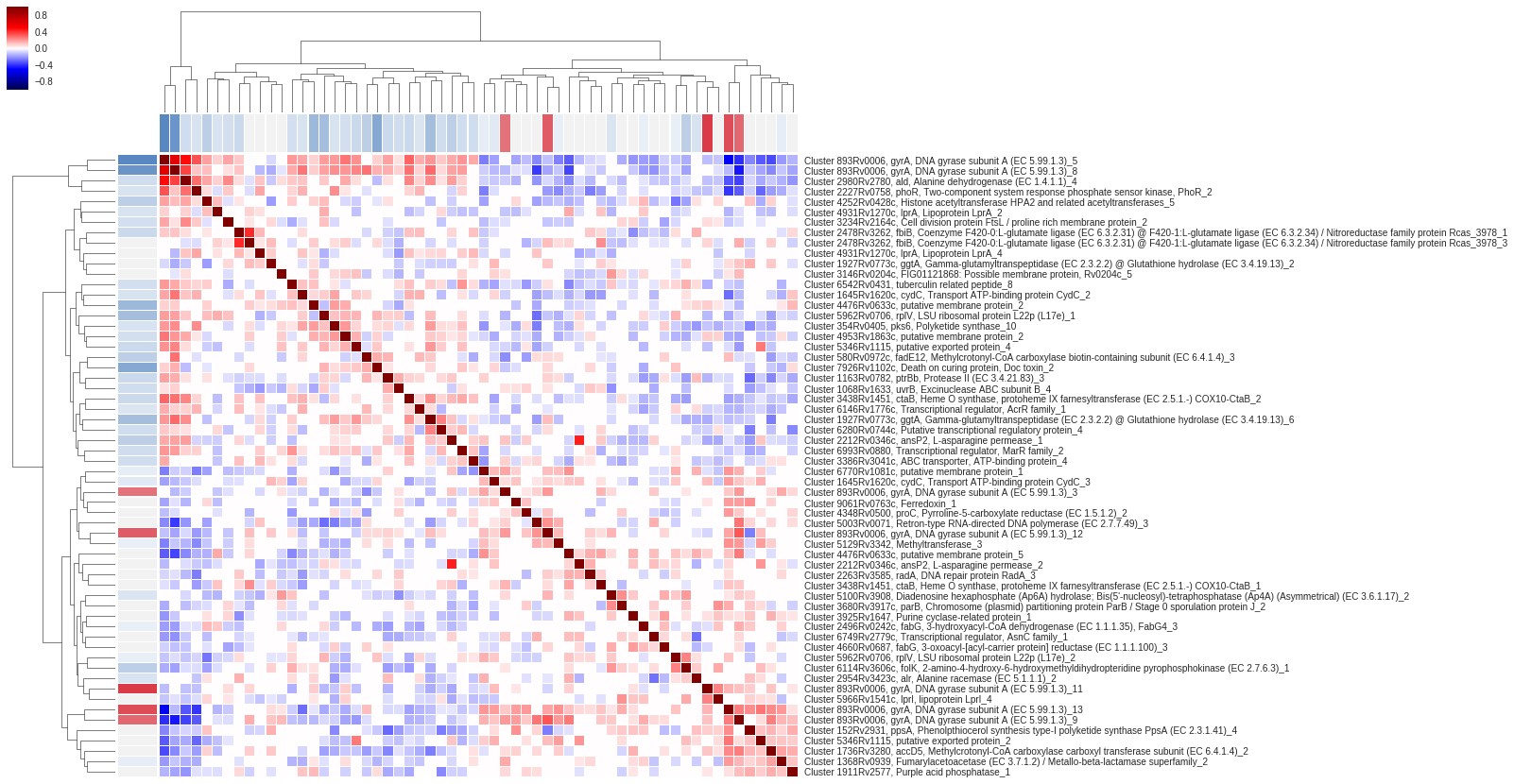

Supplement: Supplementary file 6 — Supplementary Data 3 [file 41467_2018_6634_MOESM6_ESM.zip › Supplementary Data 3/ofloxacin_SVM_SGD_correlation.png]

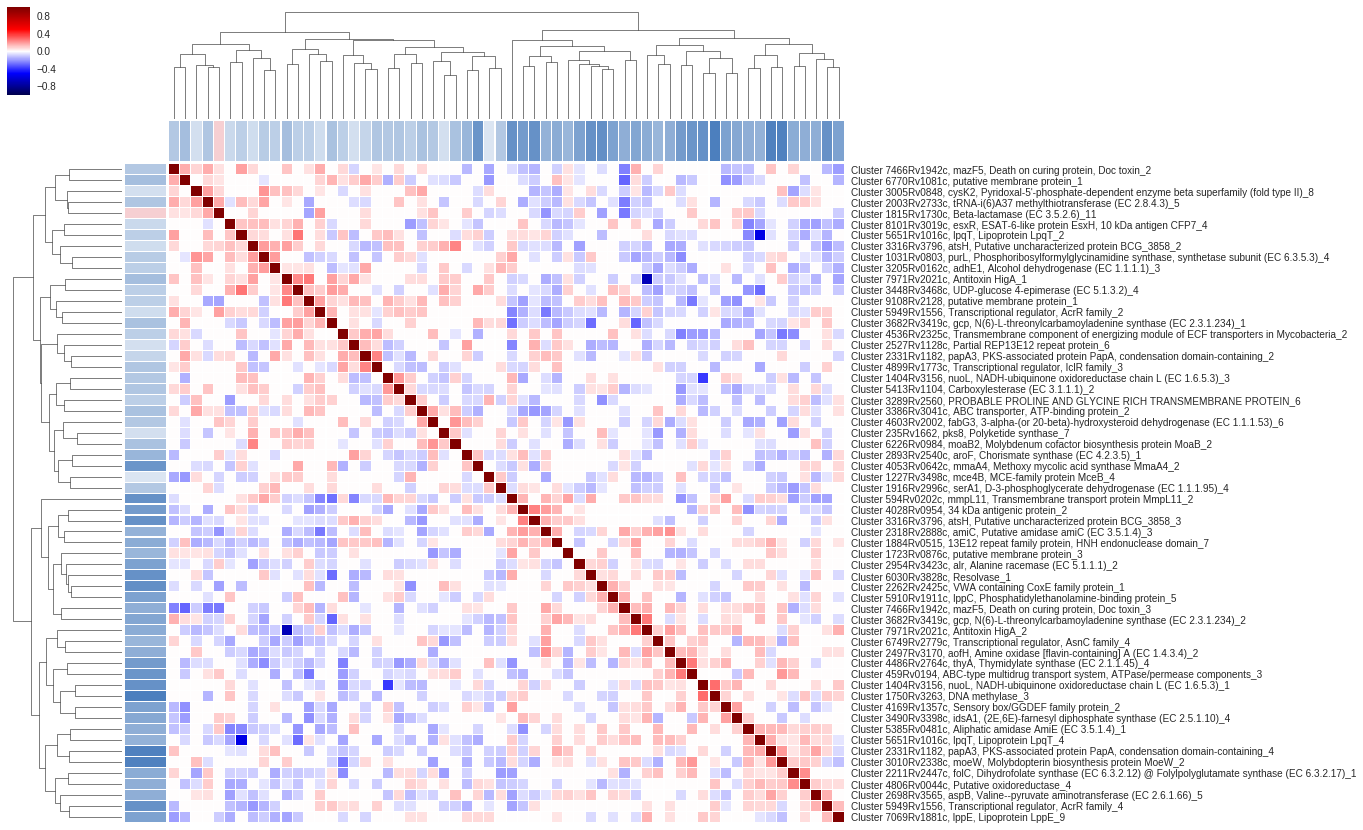

Supplement: Supplementary file 6 — Supplementary Data 3 [file 41467_2018_6634_MOESM6_ESM.zip › Supplementary Data 3/4-aminosalicylic_acid_SVM_SGD_correlation.png]

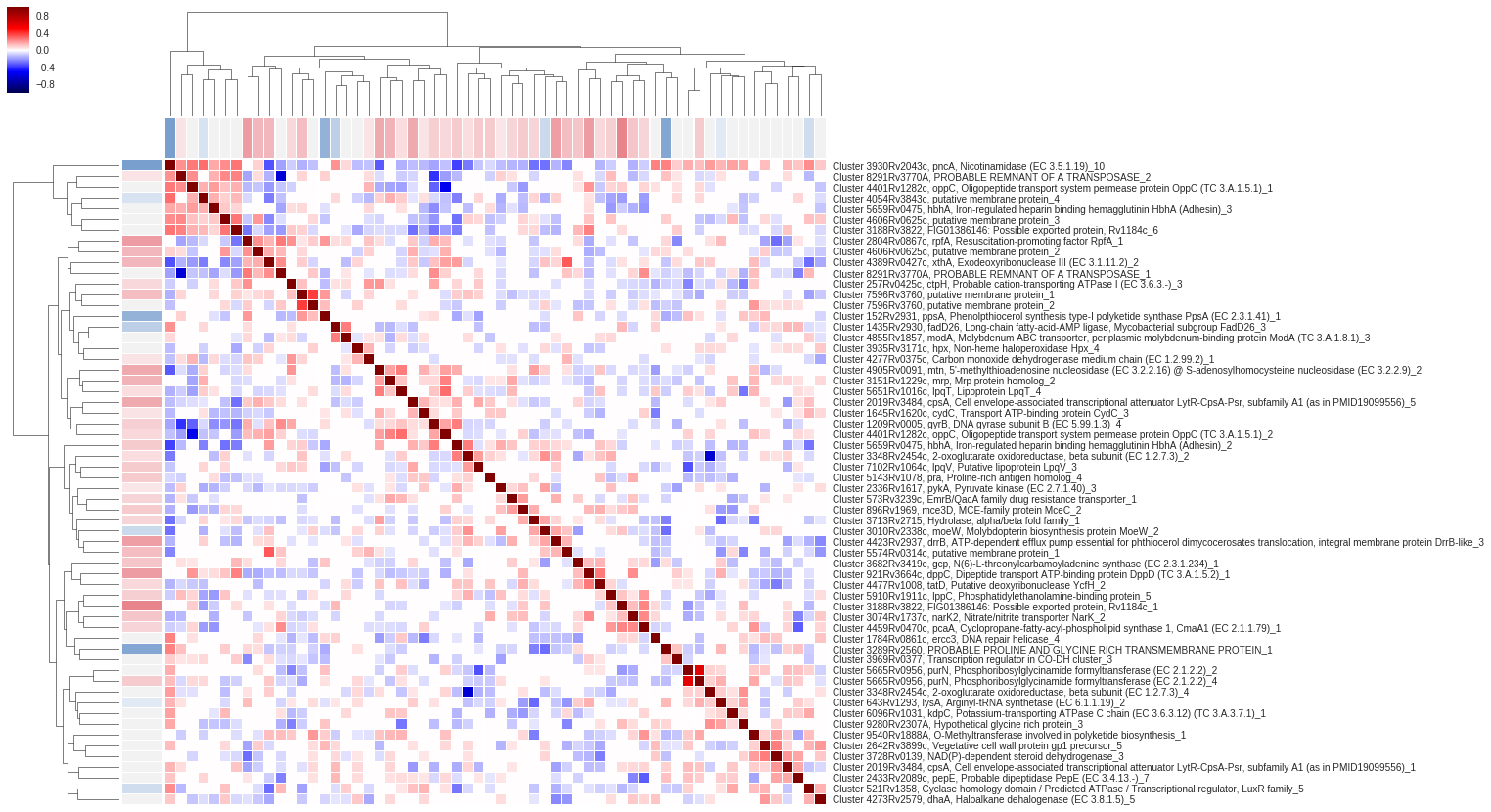

Supplement: Supplementary file 6 — Supplementary Data 3 [file 41467_2018_6634_MOESM6_ESM.zip › Supplementary Data 3/pyrazinamide_SVM_SGD_correlation.png]

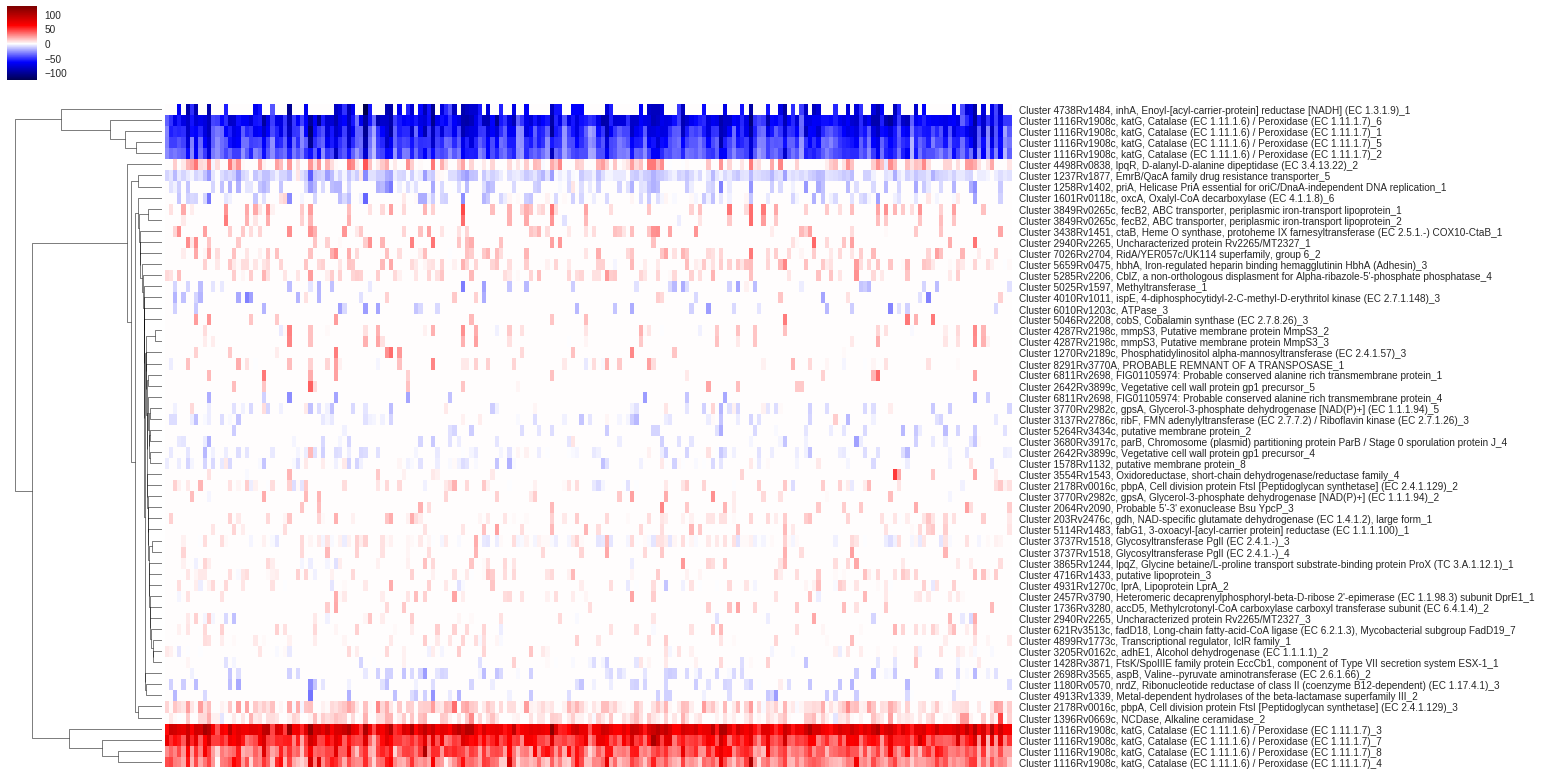

Supplement: Supplementary file 6 — Supplementary Data 3 [file 41467_2018_6634_MOESM6_ESM.zip › Supplementary Data 3/isoniazid_SVM_SGD_iterations.png]

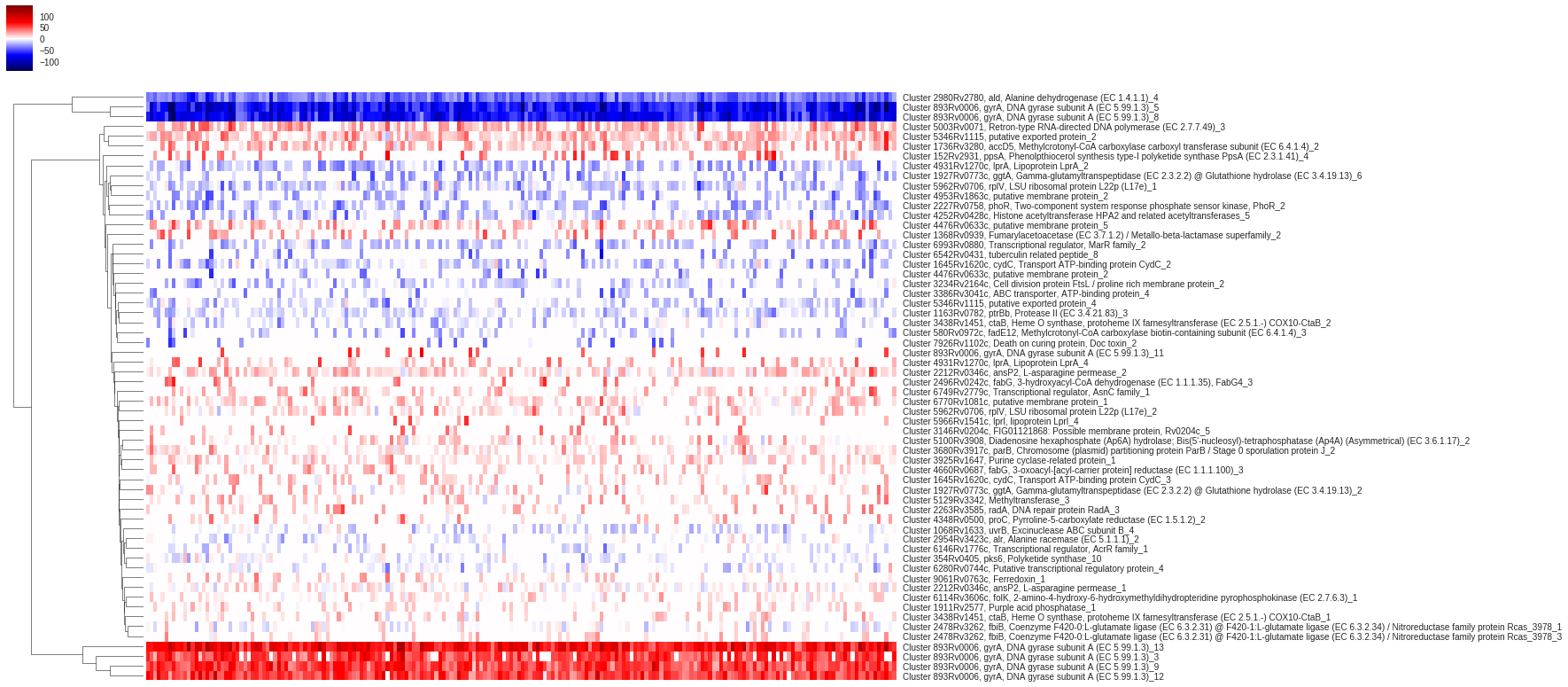

Supplement: Supplementary file 6 — Supplementary Data 3 [file 41467_2018_6634_MOESM6_ESM.zip › Supplementary Data 3/ofloxacin_SVM_SGD_iterations.png]

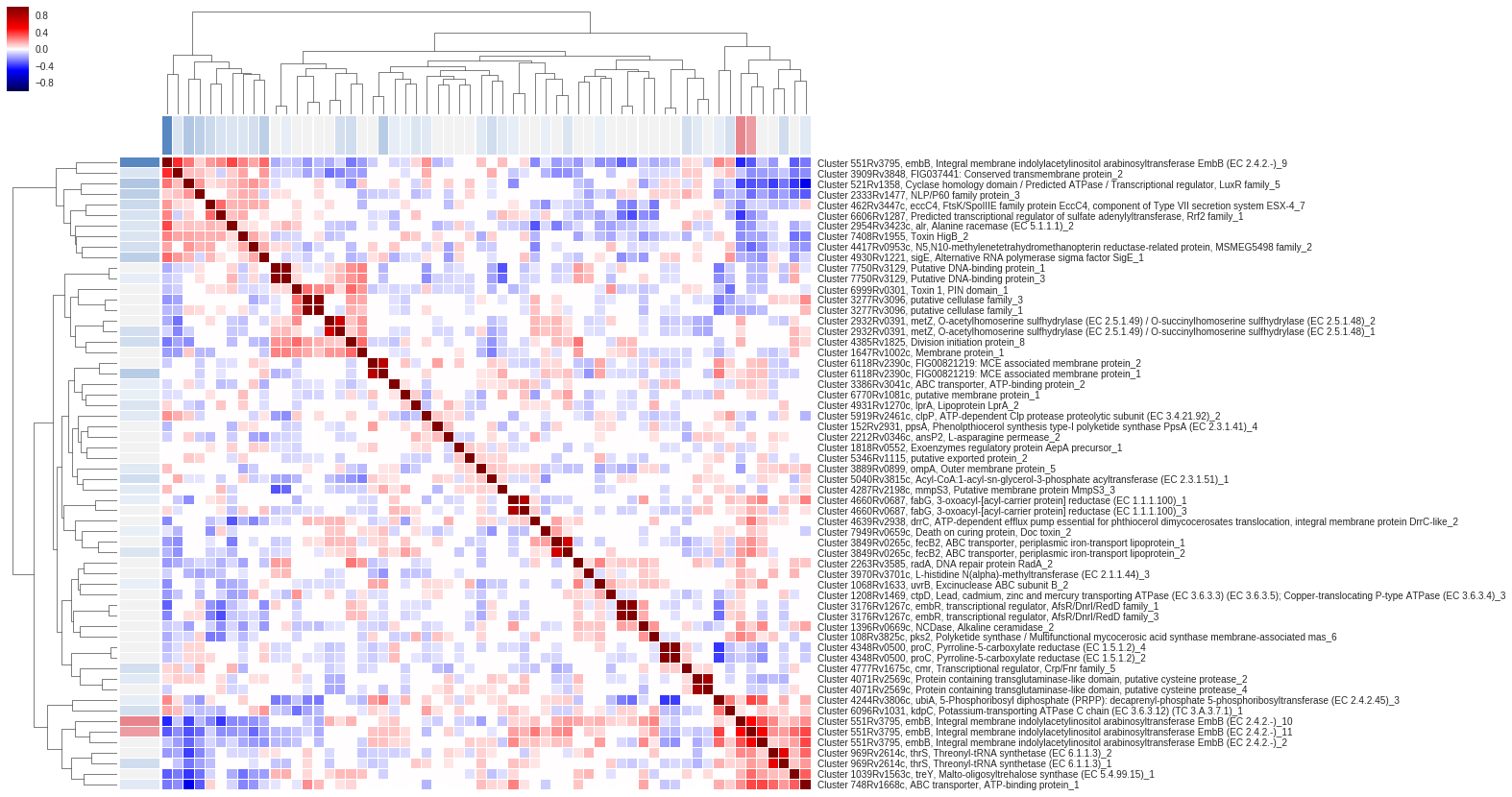

Supplement: Supplementary file 6 — Supplementary Data 3 [file 41467_2018_6634_MOESM6_ESM.zip › Supplementary Data 3/ethambutol_SVM_SGD_correlation.png]

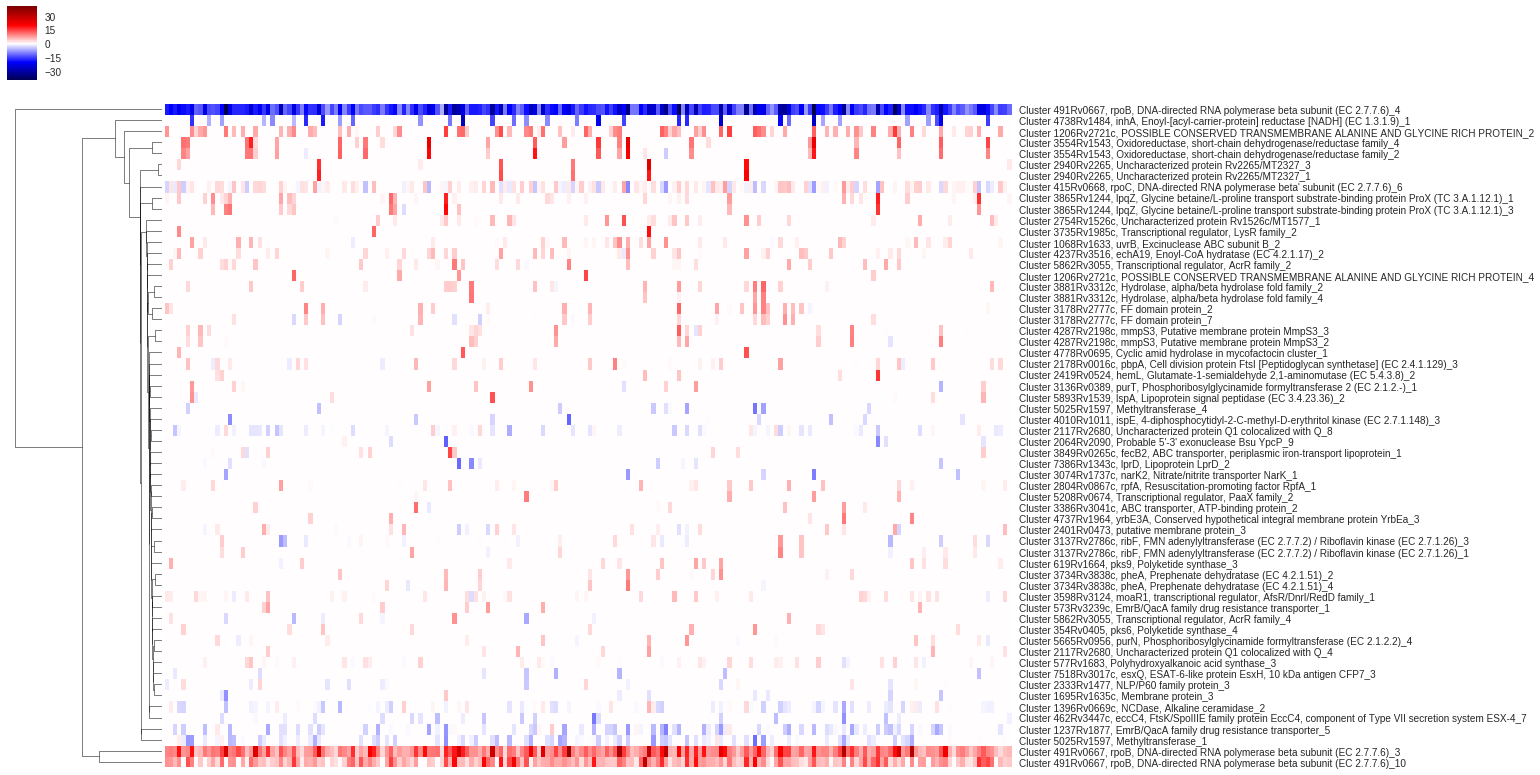

Supplement: Supplementary file 6 — Supplementary Data 3 [file 41467_2018_6634_MOESM6_ESM.zip › Supplementary Data 3/rifampicin_SVM_SGD_iterations.png]

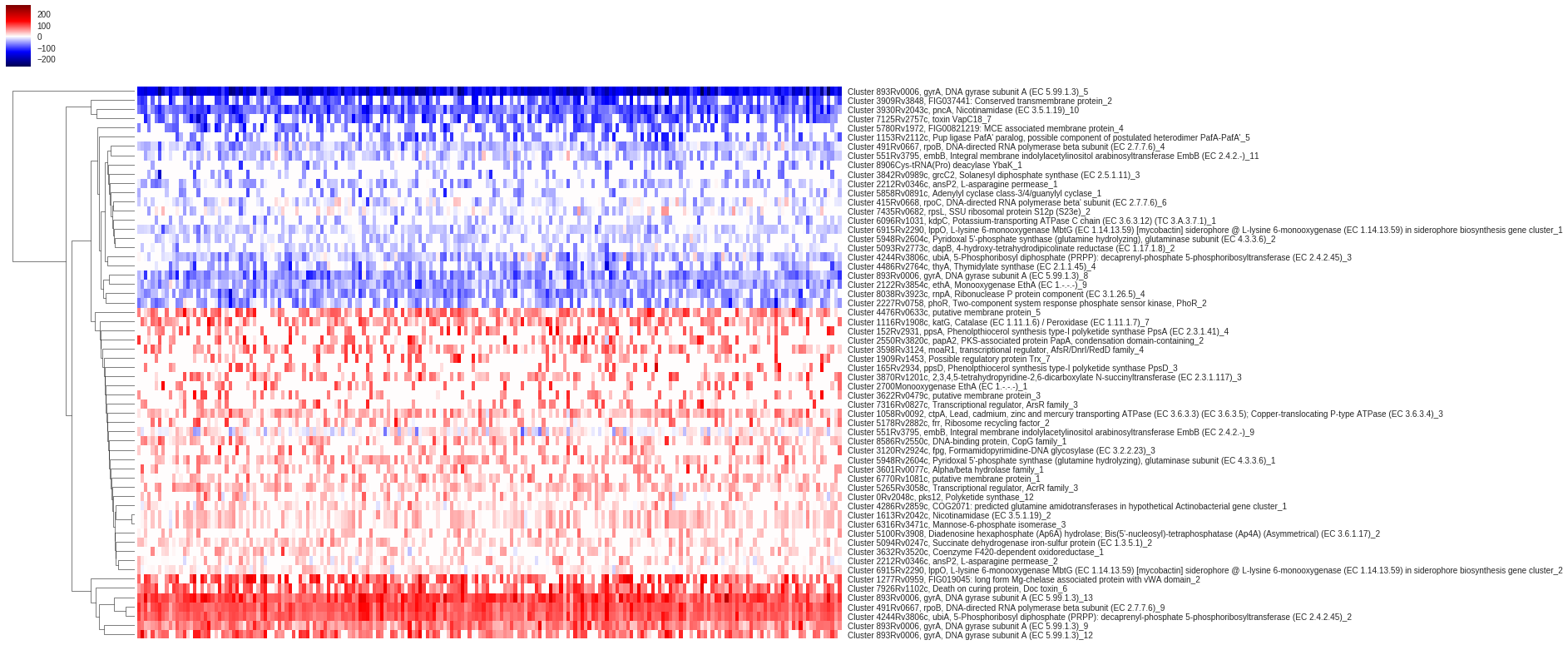

Supplement: Supplementary file 6 — Supplementary Data 3 [file 41467_2018_6634_MOESM6_ESM.zip › Supplementary Data 3/XDR_SVM_SGD_iterations.png]

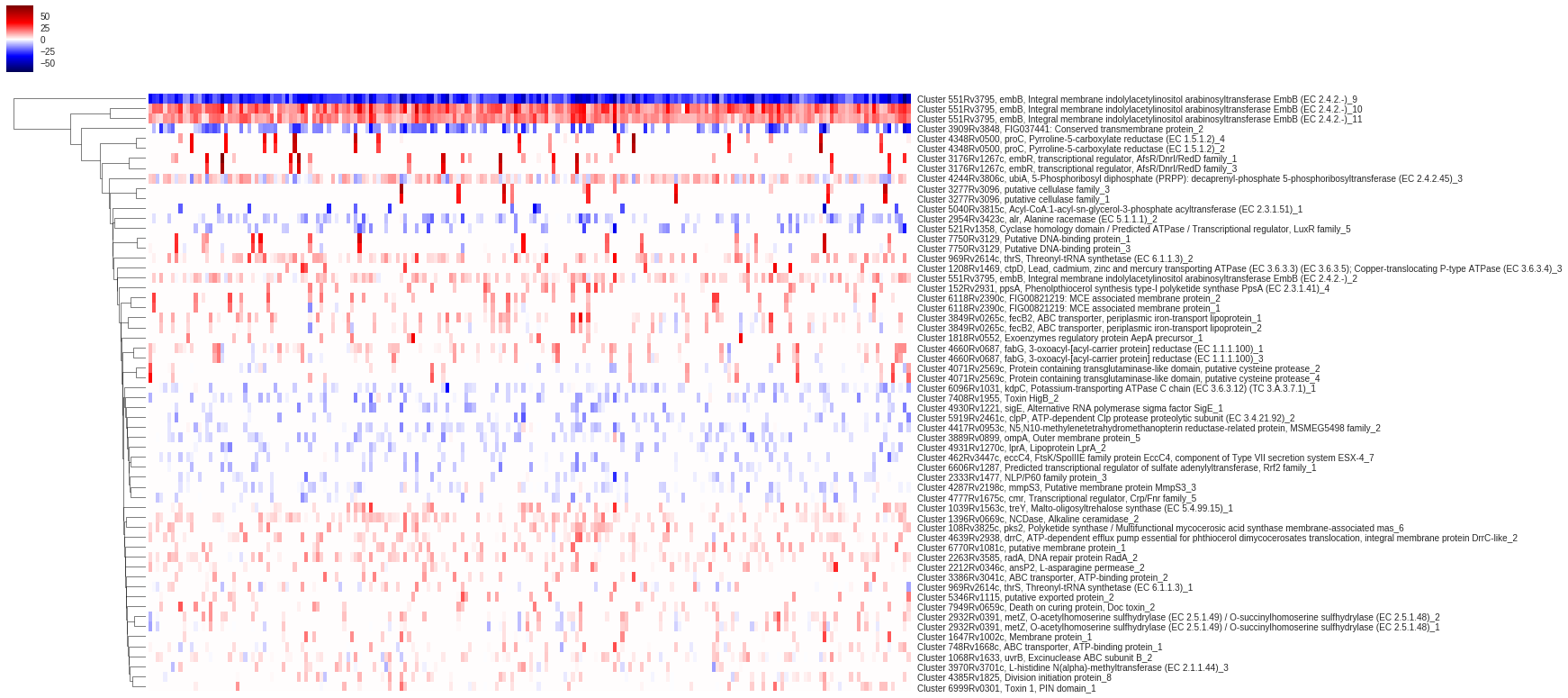

Supplement: Supplementary file 6 — Supplementary Data 3 [file 41467_2018_6634_MOESM6_ESM.zip › Supplementary Data 3/ethambutol_SVM_SGD_iterations.png]

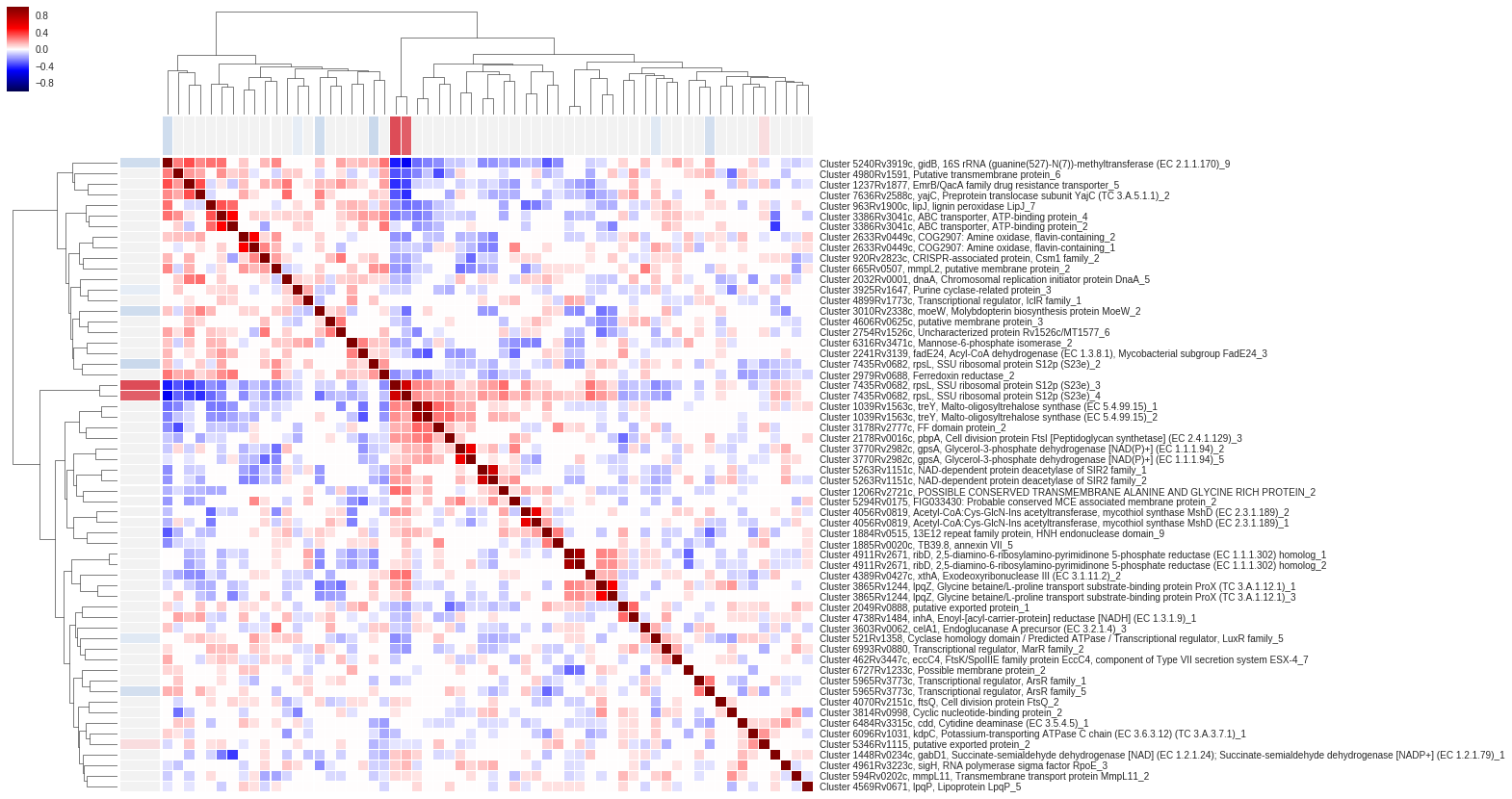

Supplement: Supplementary file 6 — Supplementary Data 3 [file 41467_2018_6634_MOESM6_ESM.zip › Supplementary Data 3/streptomycin_SVM_SGD_correlation.png]

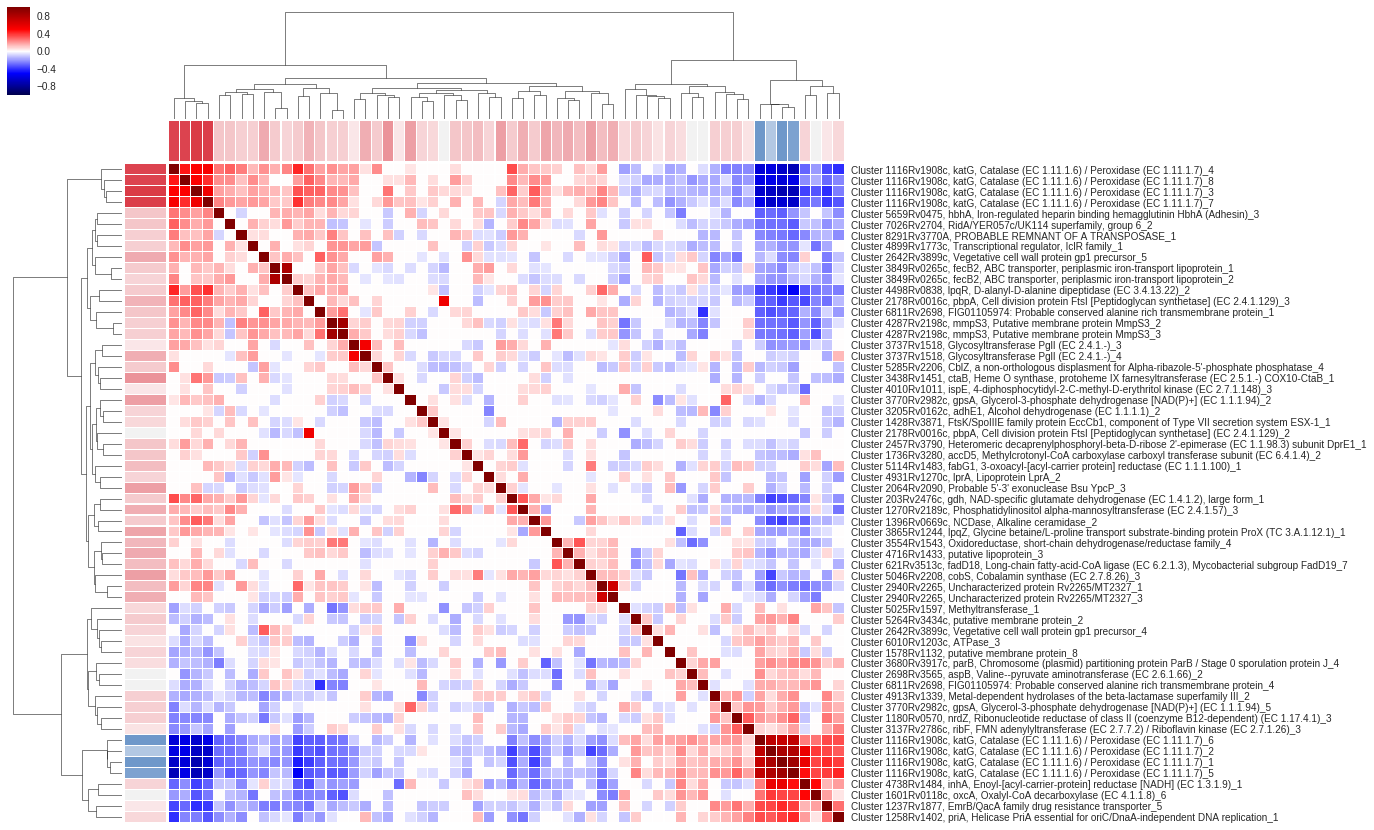

Supplement: Supplementary file 6 — Supplementary Data 3 [file 41467_2018_6634_MOESM6_ESM.zip › Supplementary Data 3/isoniazid_SVM_SGD_correlation.png]

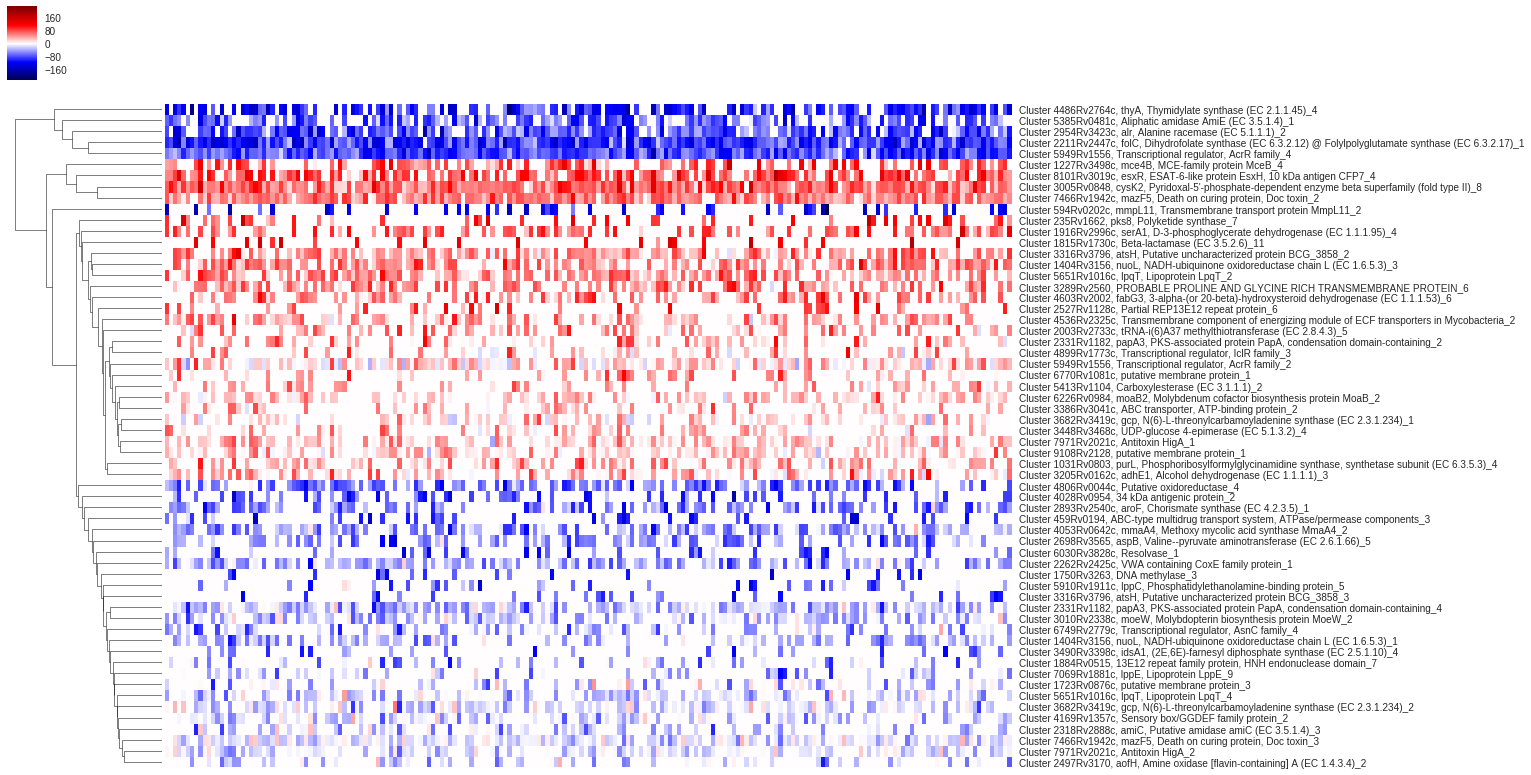

Supplement: Supplementary file 6 — Supplementary Data 3 [file 41467_2018_6634_MOESM6_ESM.zip › Supplementary Data 3/4-aminosalicylic_acid_SVM_SGD_iterations.png]

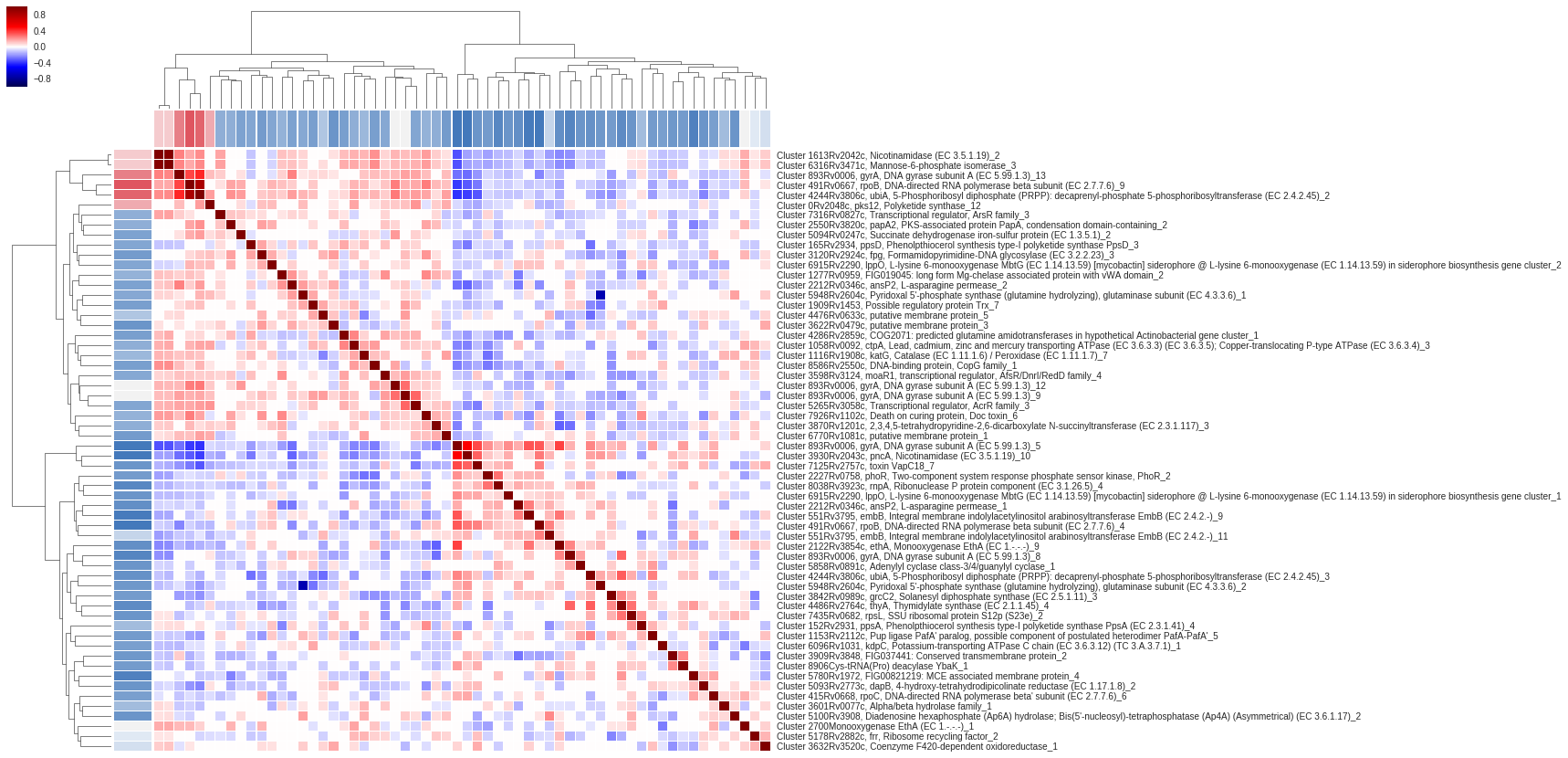

Supplement: Supplementary file 6 — Supplementary Data 3 [file 41467_2018_6634_MOESM6_ESM.zip › Supplementary Data 3/XDR_SVM_SGD_correlation.png]

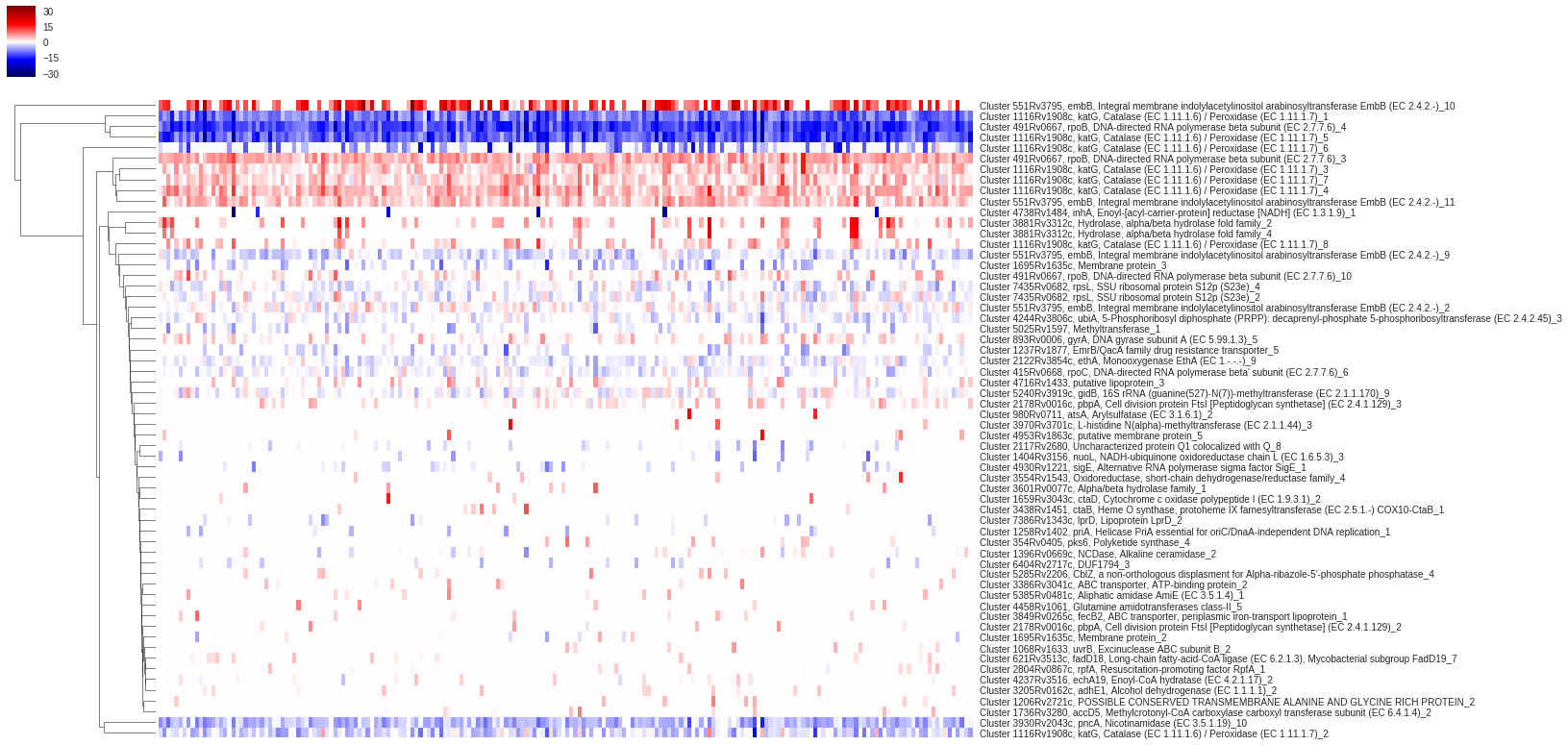

Supplement: Supplementary file 6 — Supplementary Data 3 [file 41467_2018_6634_MOESM6_ESM.zip › Supplementary Data 3/MDR_SVM_SGD_iterations.png]

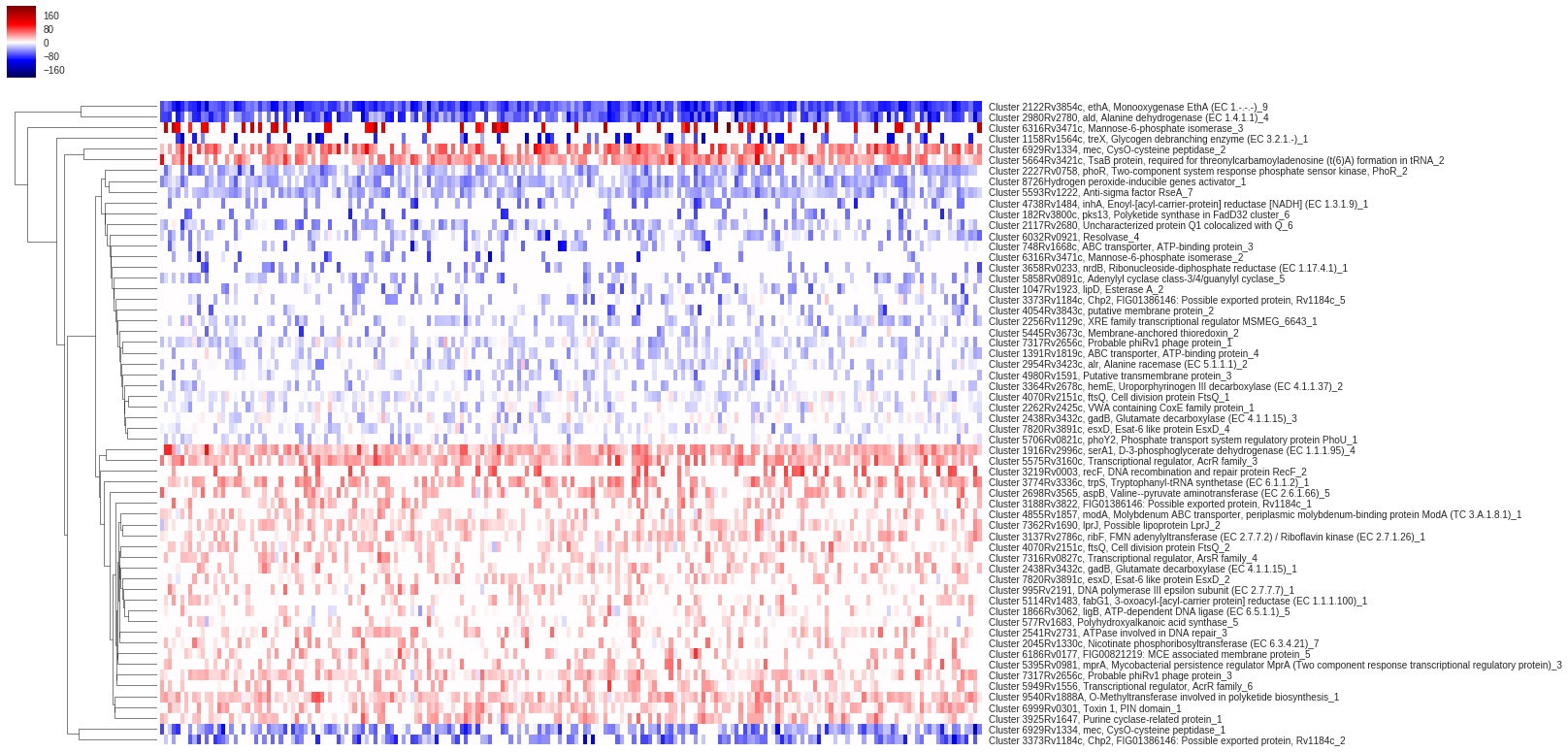

Supplement: Supplementary file 6 — Supplementary Data 3 [file 41467_2018_6634_MOESM6_ESM.zip › Supplementary Data 3/ethionamide_SVM_SGD_iterations.png]

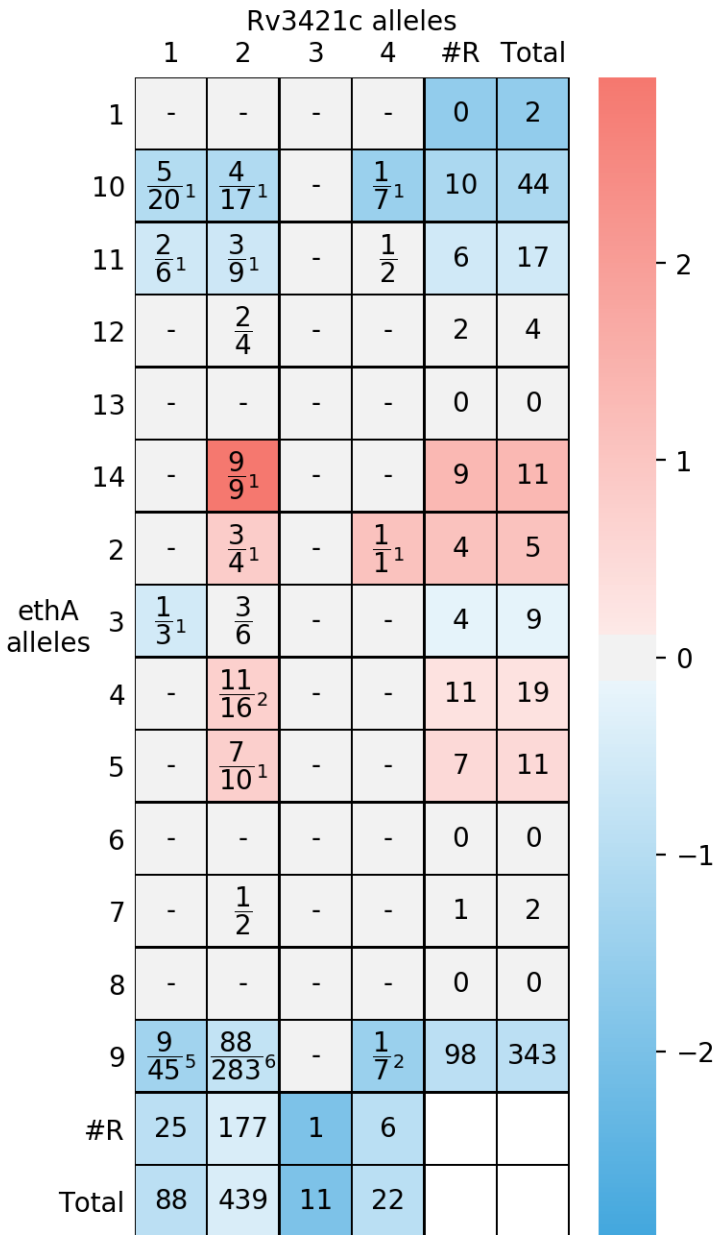

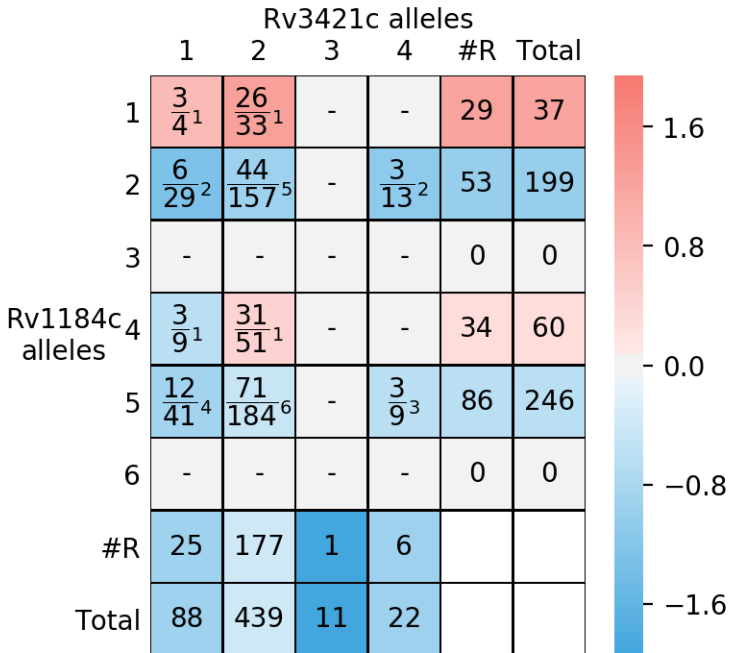

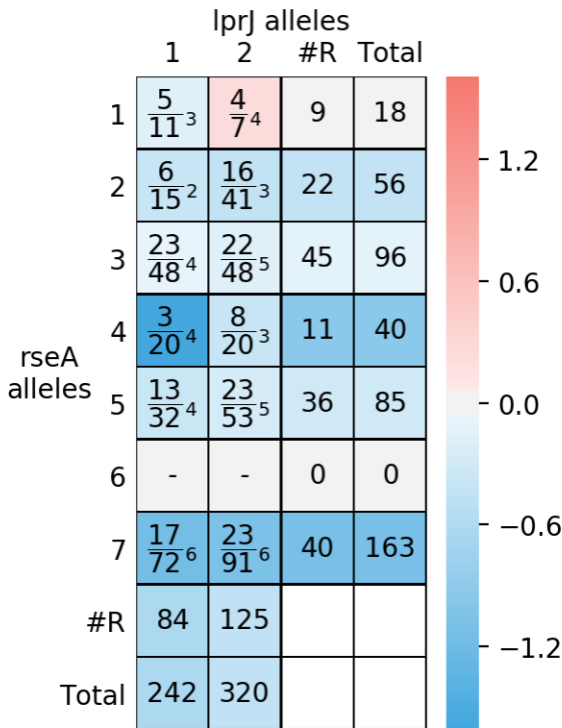

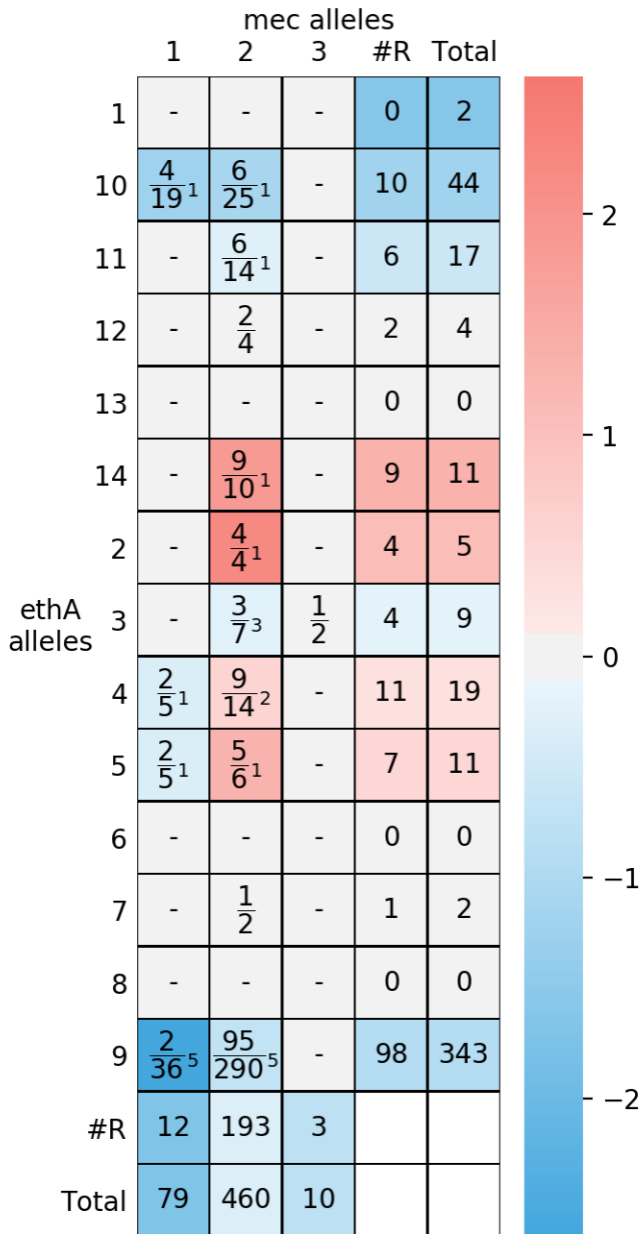

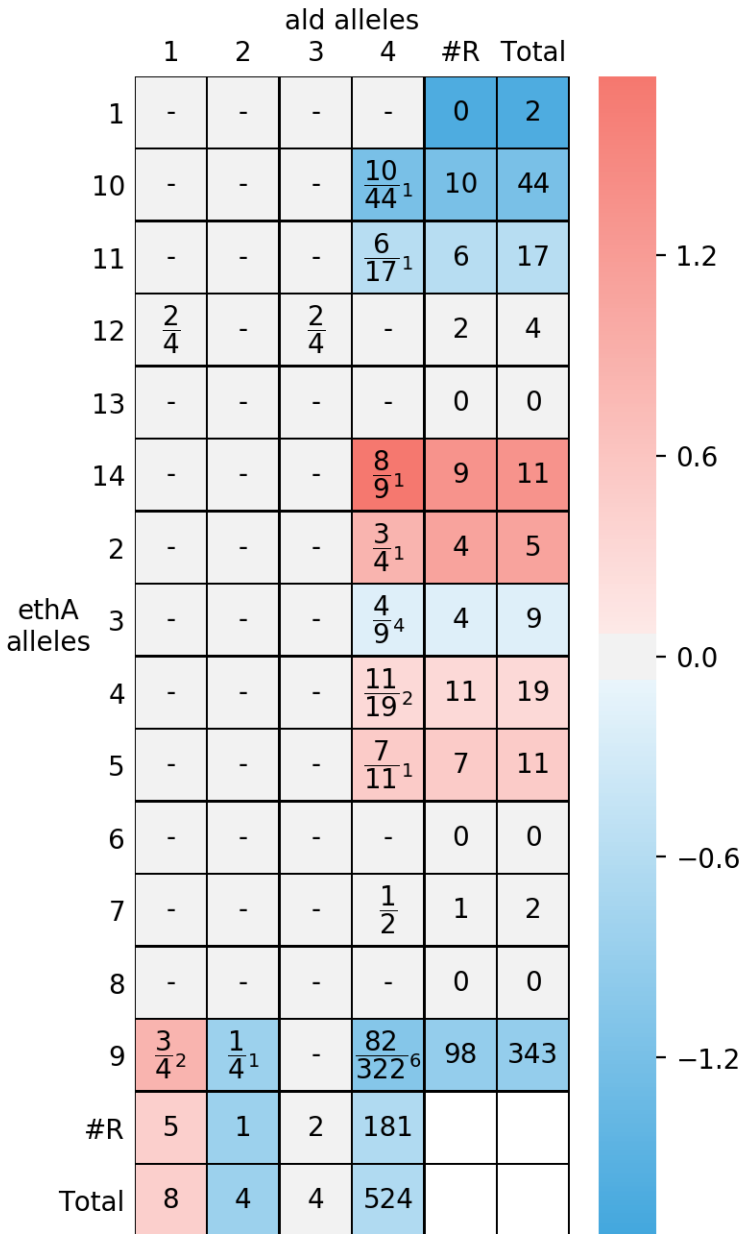

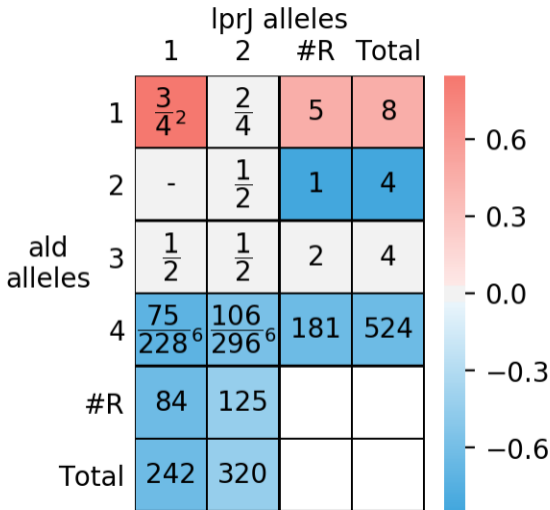

Supplement: Supplementary file 8 — Supplementary Data 5 [file 41467_2018_6634_MOESM8_ESM.zip › Supplementary Data 5/ethionamide_epistasis.pdf]

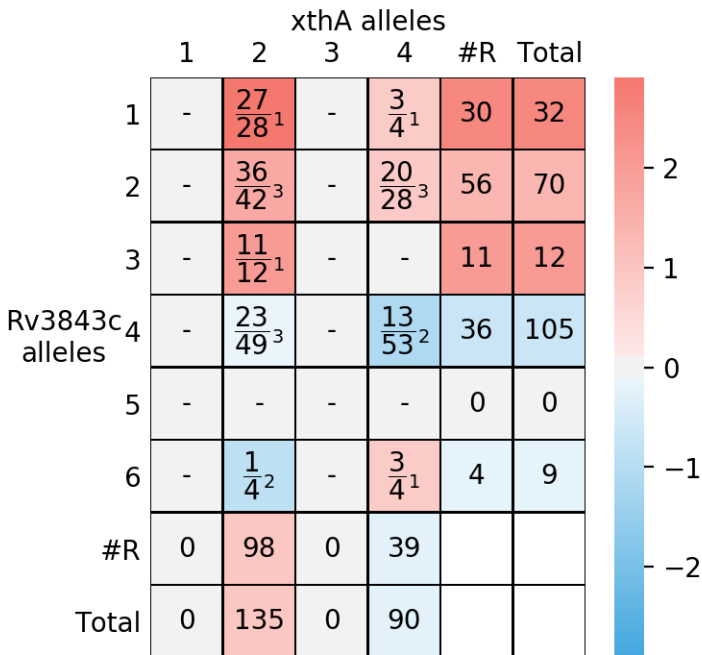

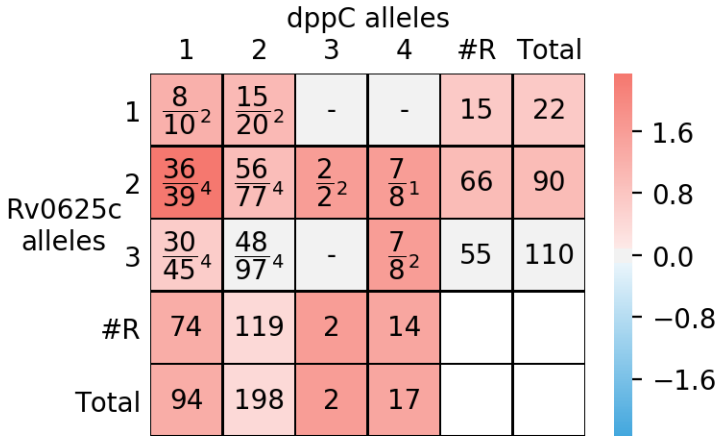

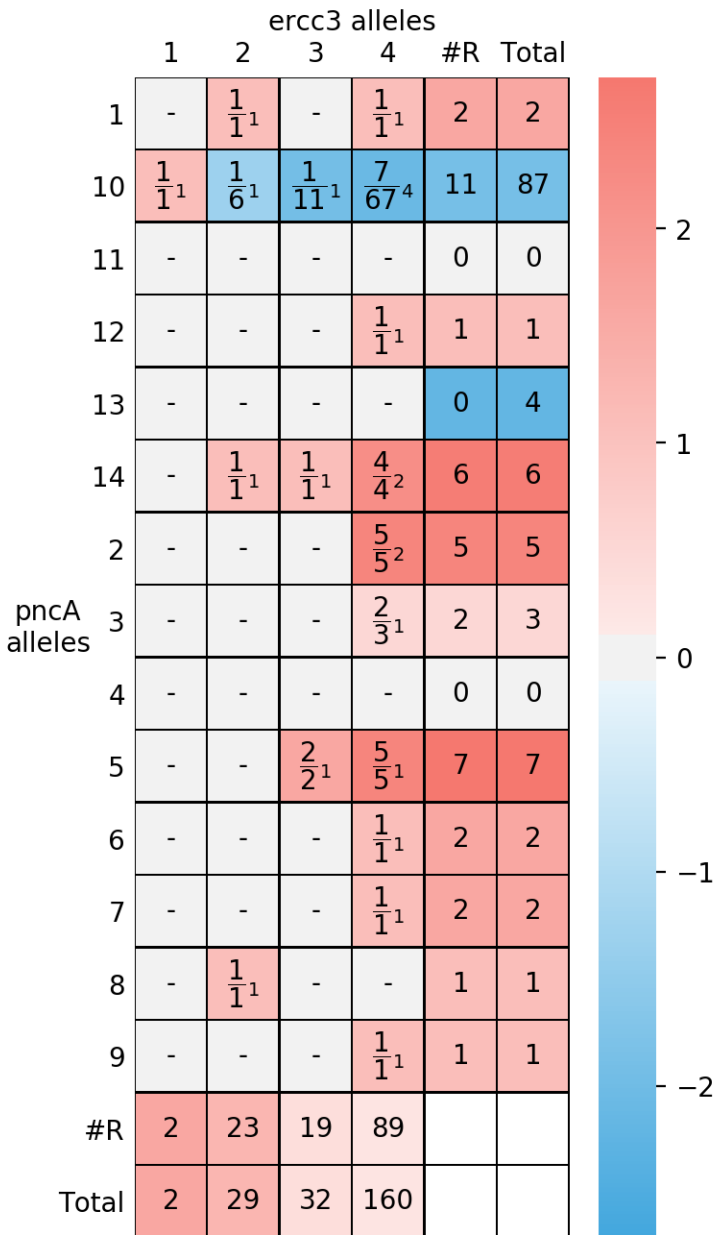

Supplement: Supplementary file 8 — Supplementary Data 5 [file 41467_2018_6634_MOESM8_ESM.zip › Supplementary Data 5/pyrazinamide_epistasis.pdf]
