# Supplementary material for: Machine learning and structural analysis of Mycobacterium tuberculosis pan-genome identifies genetic signatures of antibiotic resistance
Source: Nat Commun. 2018 Oct 17;9:4306. doi: 10.1038/s41467-018-06634-y (PMC6193043; doi:10.1038/s41467-018-06634-y)
Supplement: Supplementary file 8 — Supplementary Data 5 [file 41467_2018_6634_MOESM8_ESM.zip › Supplementary Data 5/ethambutol_epistasis.pdf]

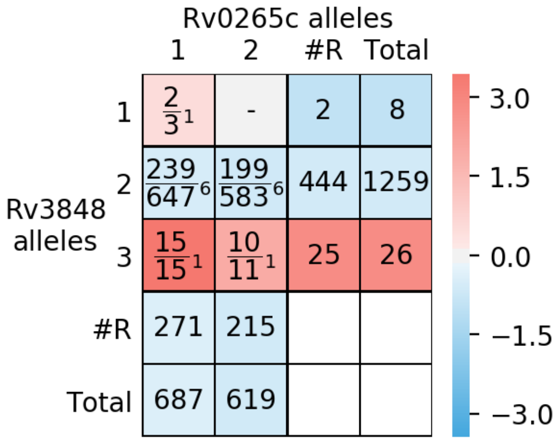
Rv0265c alleles
1
2
#R
Total
Rv3848 alleles
1
2
3
#R
Total
2/3 1
-
2
8
239/647 6
199/583 6
444
1259
15/15 1
10/11 1
25
26
271
215
687
619
3.0
1.5
0.0
−1.5
−3.0

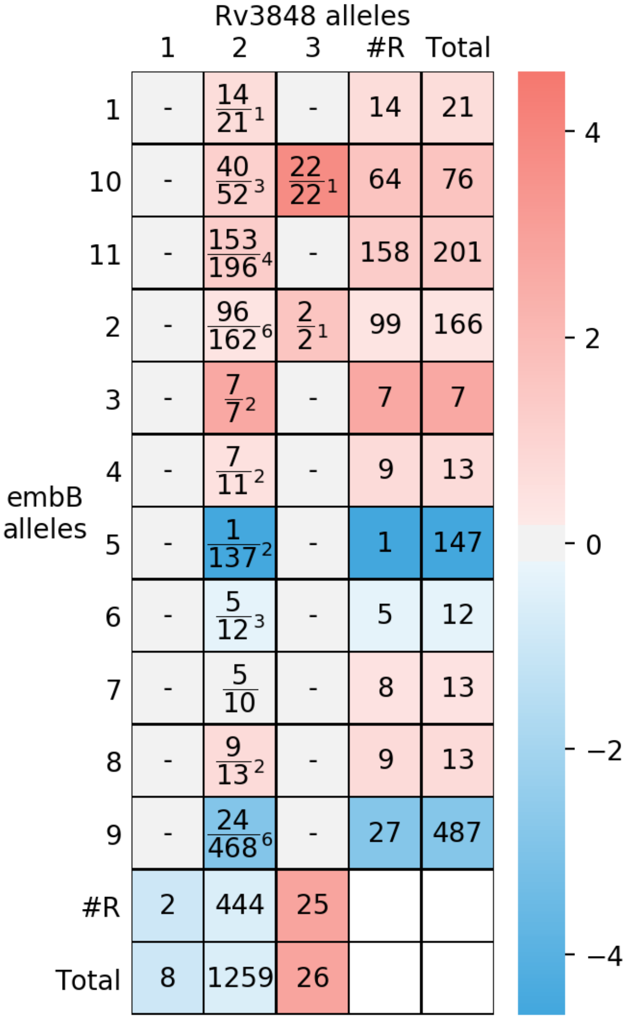
Rv3848 alleles
1
2
3
#R
Total
embB alleles
1
10
11
2
3
4
5
6
7
8
9
#R
Total
-
14/21 1
-
14
21
-
40/52 3
22/22 1
64
76
-
153/196 4
-
158
201
-
96/162 6
2/2 1
99
166
-
7/7 2
-
7
7
-
7/11 2
-
9
13
-
1/137 2
-
1
147
-
5/12 3
-
5
12
-
5/10
-
8
13
-
9/13 2
-
9
13
-
24/468 6
-
27
487
2
444
25
8
1259
26
4
2
0
−2
−4

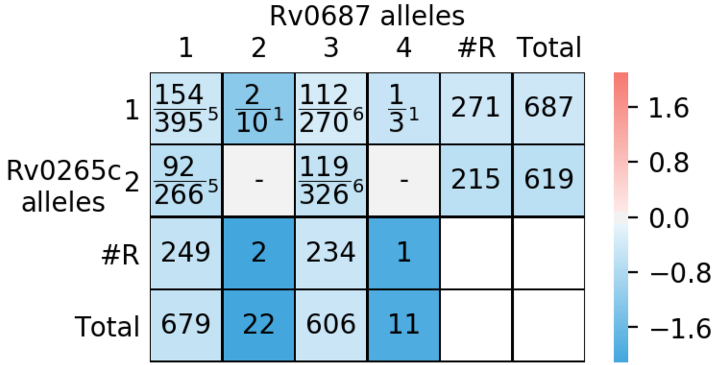
Rv0687 alleles
1
2
3
4
#R
Total
Rv0265c alleles
1
154/395 5
2/10 1
112/270 6
1/3 1
271
687
2
92/266 5
-
119/326 6
-
215
619
#R
249
2
234
1
Total
679
22
606
11
1.6
0.8
0.0
−0.8
−1.6

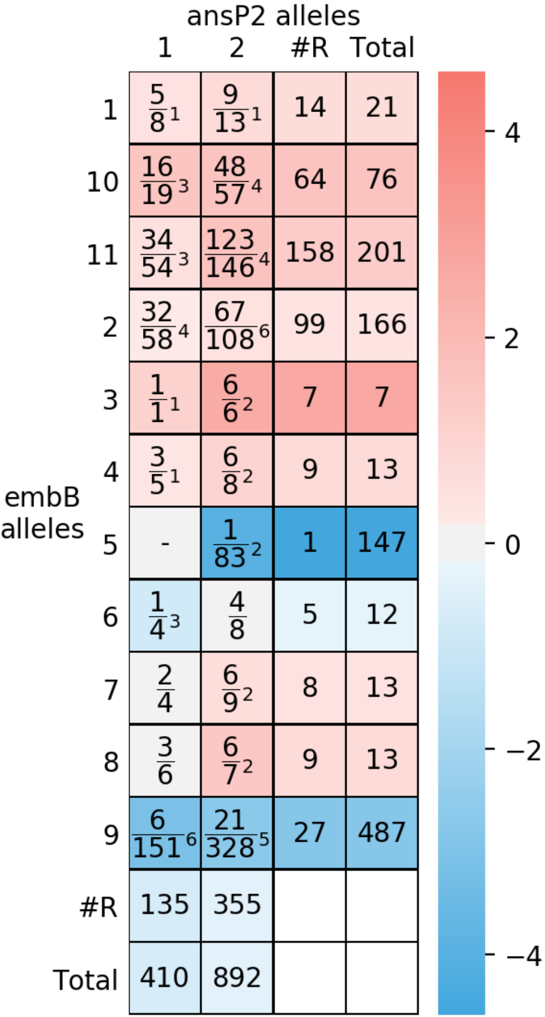
ansP2 alleles
1
2
#R
Total
embB alleles
1: 5/8 1, 9/13 1, 14, 21
10: 16/19 3, 48/57 4, 64, 76
11: 34/54 3, 123/146 4, 158, 201
2: 32/58 4, 67/108 6, 99, 166
3: 1/1 1, 6/6 2, 7, 7
4: 3/5 1, 6/8 2, 9, 13
5: -, 1/83 2, 1, 147
6: 1/4 3, 4/8, 5, 12
7: 2/4, 6/9 2, 8, 13
8: 3/6, 6/7 2, 9, 13
9: 6/151 6, 21/328 5, 27, 487
#R: 135, 355
Total: 410, 892
4
2
0
−2
−4

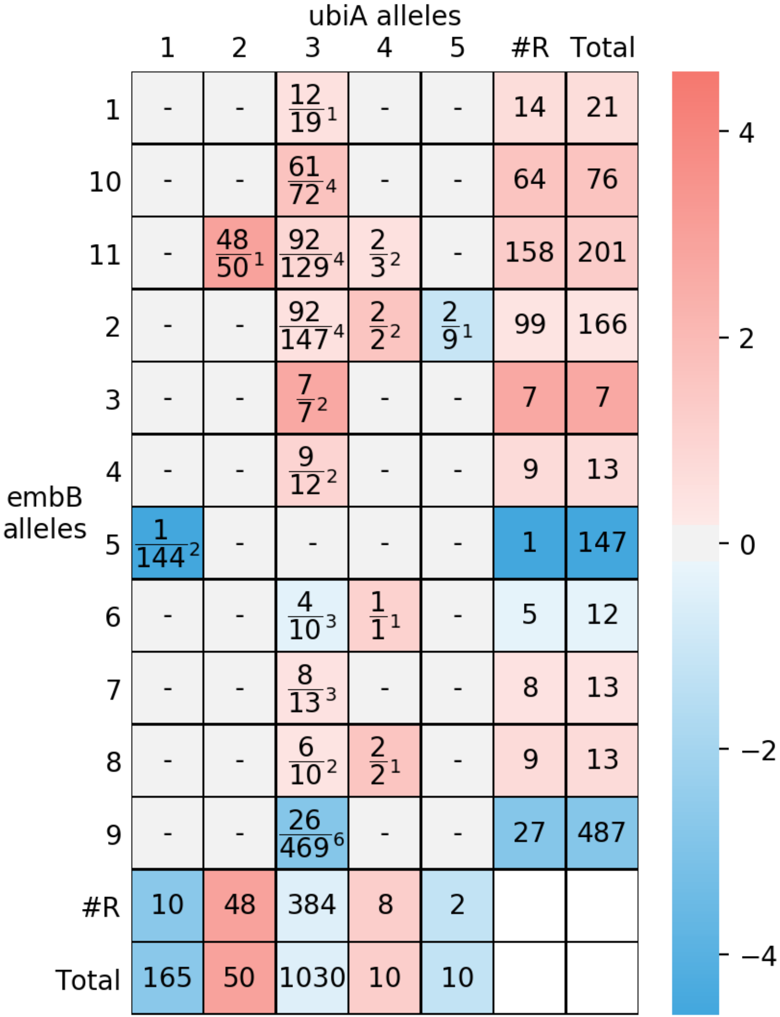

ubiA alleles
1 2 3 4 5 #R Total
embB alleles
1 - - 12/19 1 - - 14 21
10 - - 61/72 4 - - 64 76
11 - 48/50 1 92/129 4 2/3 2 - 158 201
2 - - 92/147 4 2/2 2 2/9 1 99 166
3 - - 7/7 2 - - 7 7
4 - - 9/12 2 - - 9 13
5 1/144 2 - - - - 1 147
6 - - 4/10 3 1/1 1 - 5 12
7 - - 8/13 3 - - 8 13
8 - - 6/10 2 2/2 1 - 9 13
9 - - 26/469 6 - - 27 487
#R 10 48 384 8 2
Total 165 50 1030 10 10
4
2
0
−2
−4

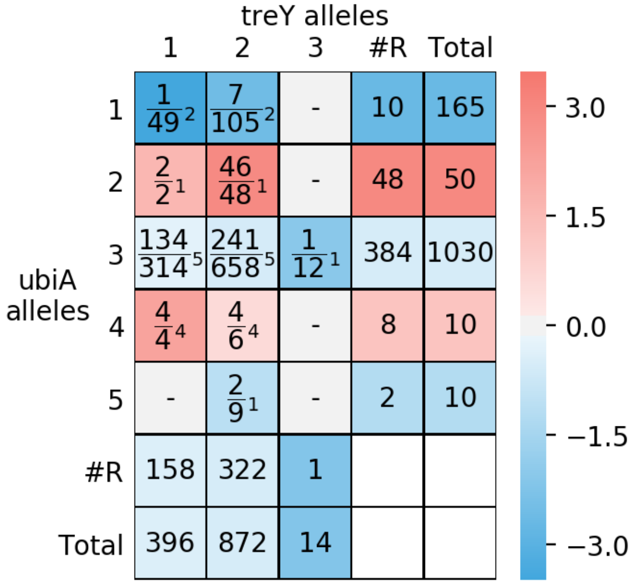
treY alleles
1
2
3
#R
Total
ubiA alleles
1
2
3
4
5
#R
Total
1/49 2
7/105 2
-
10
165
2/2 1
46/48 1
-
48
50
134/314 5
241/658 5
1/12 1
384
1030
4/4 4
4/6 4
-
8
10
-
2/9 1
-
2
10
158
322
1
396
872
14
3.0
1.5
0.0
−1.5
−3.0

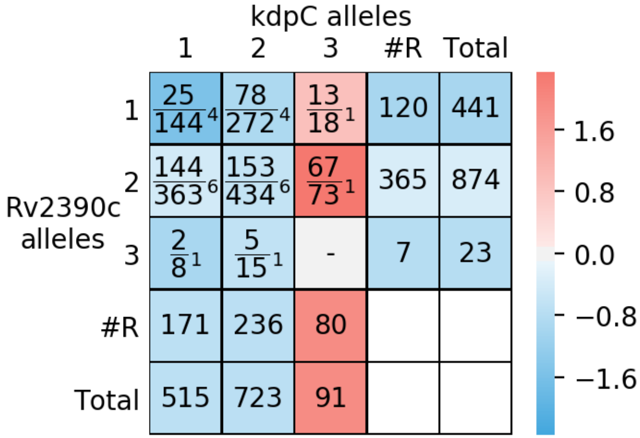
kdpC alleles
1
2
3
#R
Total
Rv2390c alleles
1
2
3
#R
Total
25/144 4
78/272 4
13/18 1
120
441
144/363 6
153/434 6
67/73 1
365
874
2/8 1
5/15 1
-
7
23
171
236
80
515
723
91
1.6
0.8
0.0
−0.8
−1.6

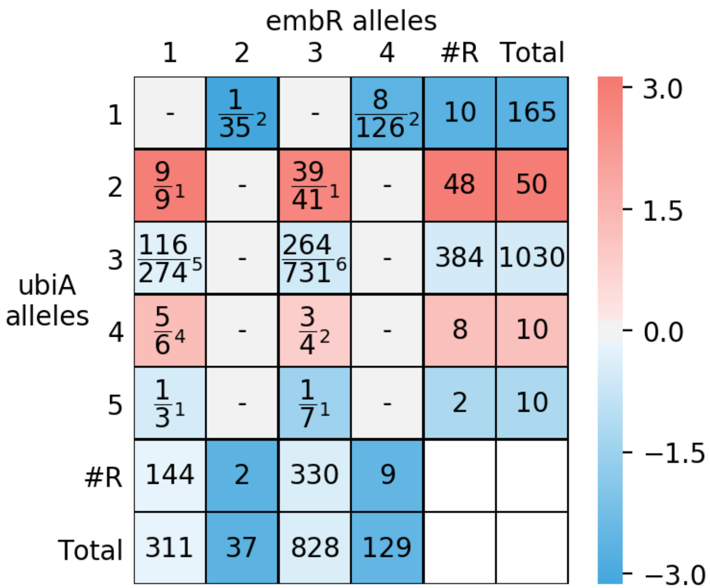

embR alleles
1
2
3
4
#R
Total
ubiA alleles
1
-
1/35 2
-
8/126 2
10
165
2
9/9 1
-
39/41 1
-
48
50
3
116/274 5
-
264/731 6
-
384
1030
4
5/6 4
-
3/4 2
-
8
10
5
1/3 1
-
1/7 1
-
2
10
#R
144
2
330
9
Total
311
37
828
129
3.0
1.5
0.0
−1.5
−3.0
