# Supplementary material for: Machine learning and structural analysis of Mycobacterium tuberculosis pan-genome identifies genetic signatures of antibiotic resistance
Source: Nat Commun. 2018 Oct 17;9:4306. doi: 10.1038/s41467-018-06634-y (PMC6193043; doi:10.1038/s41467-018-06634-y)
Supplement: Supplementary file 8 — Supplementary Data 5 [file 41467_2018_6634_MOESM8_ESM.zip › Supplementary Data 5/ofloxacin_epistasis.pdf]

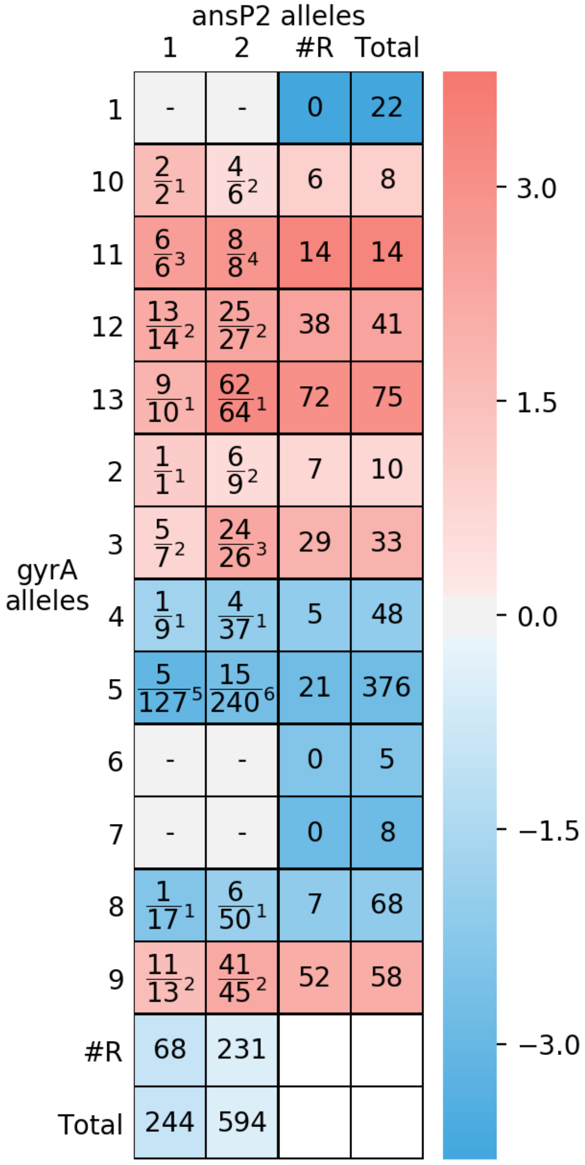
ansP2 alleles
1
2
#R
Total
gyrA alleles
1
-
-
0
22
10
2/2 1
4/6 2
6
8
11
6/6 3
8/8 4
14
14
12
13/14 2
25/27 2
38
41
13
9/10 1
62/64 1
72
75
2
1/1 1
6/9 2
7
10
3
5/7 2
24/26 3
29
33
4
1/9 1
4/37 1
5
48
5
5/127 5
15/240 6
21
376
6
-
-
0
5
7
-
-
0
8
8
1/17 1
6/50 1
7
68
9
11/13 2
41/45 2
52
58
#R
68
231
Total
244
594
3.0
1.5
0.0
−1.5
−3.0

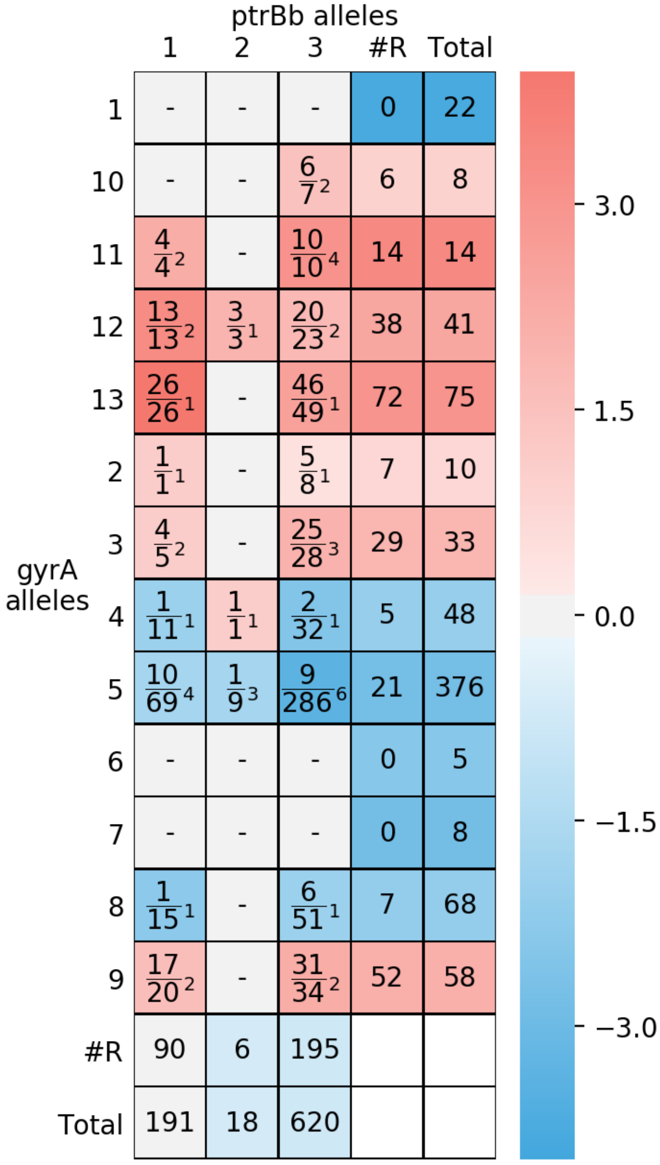
ptrBb alleles
1
2
3
#R
Total
gyrA alleles
1
10
11
12
13
2
3
4
5
6
7
8
9
#R
Total
- - - 0 22
- - 6/7 2 6 8
4/4 2 - 10/10 4 14 14
13/13 2 3/3 1 20/23 2 38 41
26/26 1 - 46/49 1 72 75
1/1 1 - 5/8 1 7 10
4/5 2 - 25/28 3 29 33
1/11 1 1/1 1 2/32 1 5 48
10/69 4 1/9 3 9/286 6 21 376
- - - 0 5
- - - 0 8
1/15 1 - 6/51 1 7 68
17/20 2 - 31/34 2 52 58
90 6 195
191 18 620
3.0
1.5
0.0
−1.5
−3.0

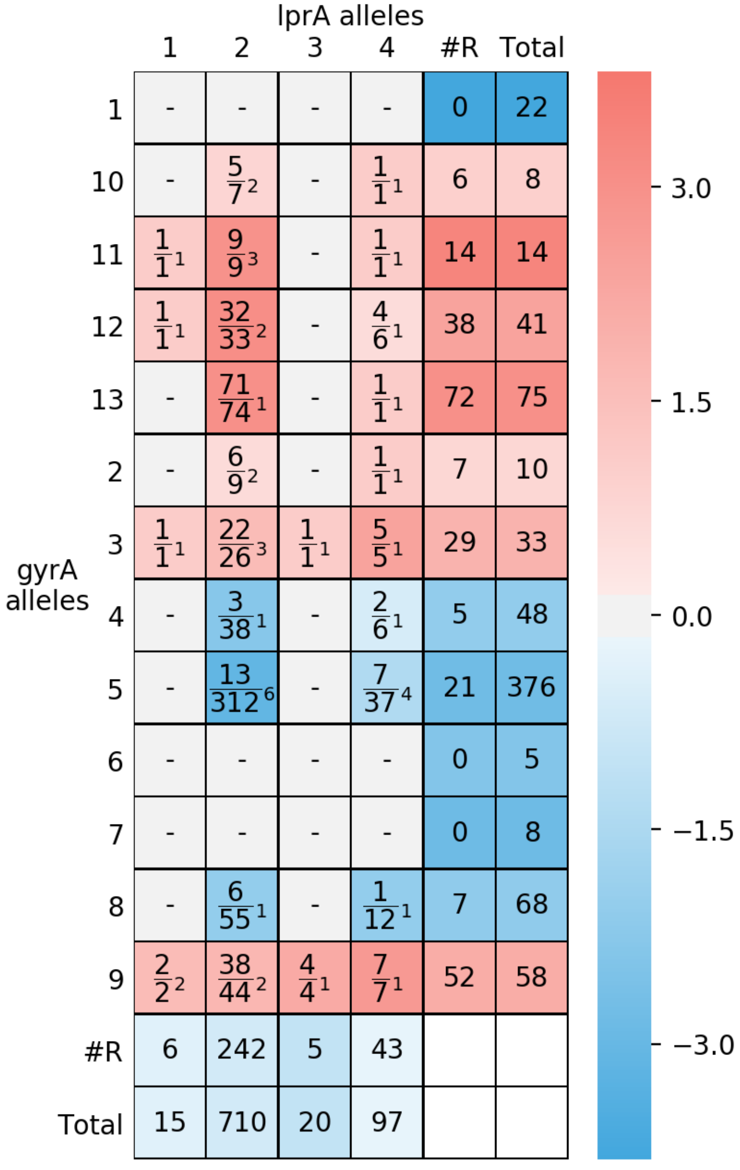

lprA alleles
1 2 3 4 #R Total
gyrA alleles
1: - - - - 0 22
10: - 5/7 2 - 1/1 1 6 8
11: 1/1 1 9/9 3 - 1/1 1 14 14
12: 1/1 1 32/33 2 - 4/6 1 38 41
13: - 71/74 1 - 1/1 1 72 75
2: - 6/9 2 - 1/1 1 7 10
3: 1/1 1 22/26 3 1/1 1 5/5 1 29 33
4: - 3/38 1 - 2/6 1 5 48
5: - 13/312 6 - 7/37 4 21 376
6: - - - - 0 5
7: - - - - 0 8
8: - 6/55 1 - 1/12 1 7 68
9: 2/2 2 38/44 2 4/4 1 7/7 1 52 58
#R: 6 242 5 43
Total: 15 710 20 97
3.0
1.5
0.0
−1.5
−3.0

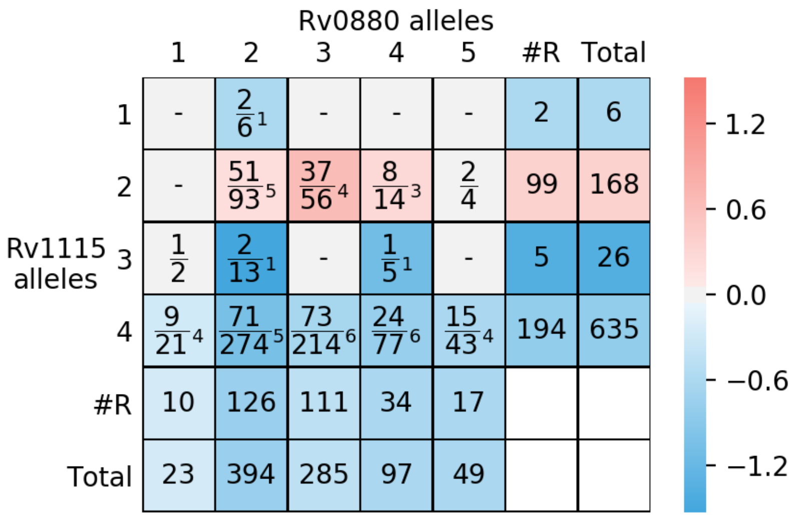

Rv0880 alleles
1 2 3 4 5 #R Total
Rv1115 alleles
1: - | 2/6 1 | - | - | - | 2 | 6
2: - | 51/93 5 | 37/56 4 | 8/14 3 | 2/4 | 99 | 168
3: 1/2 | 2/13 1 | - | 1/5 1 | - | 5 | 26
4: 9/21 4 | 71/274 5 | 73/214 6 | 24/77 6 | 15/43 4 | 194 | 635
#R: 10 | 126 | 111 | 34 | 17
Total: 23 | 394 | 285 | 97 | 49
1.2
0.6
0.0
−0.6
−1.2

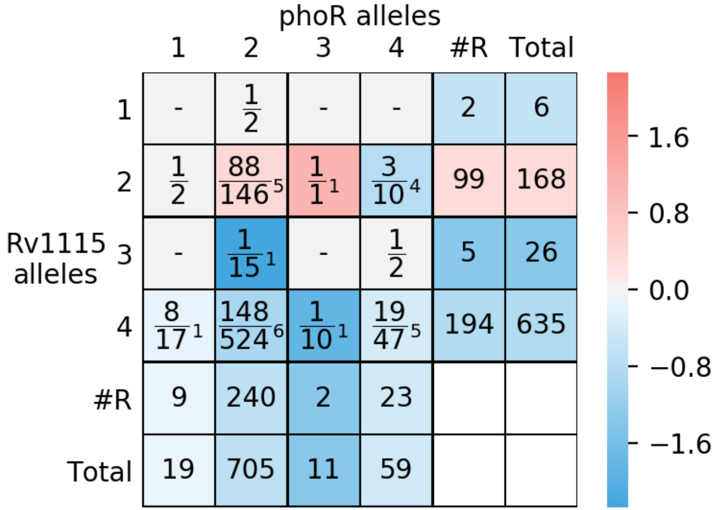

phoR alleles
1 2 3 4 #R Total
Rv1115 alleles
1: - 1/2 - - 2 6
2: 1/2 88/146 5 1/1 1 3/10 4 99 168
3: - 1/15 1 - 1/2 5 26
4: 8/17 1 148/524 6 1/10 1 19/47 5 194 635
#R: 9 240 2 23
Total: 19 705 11 59
1.6
0.8
0.0
−0.8
−1.6

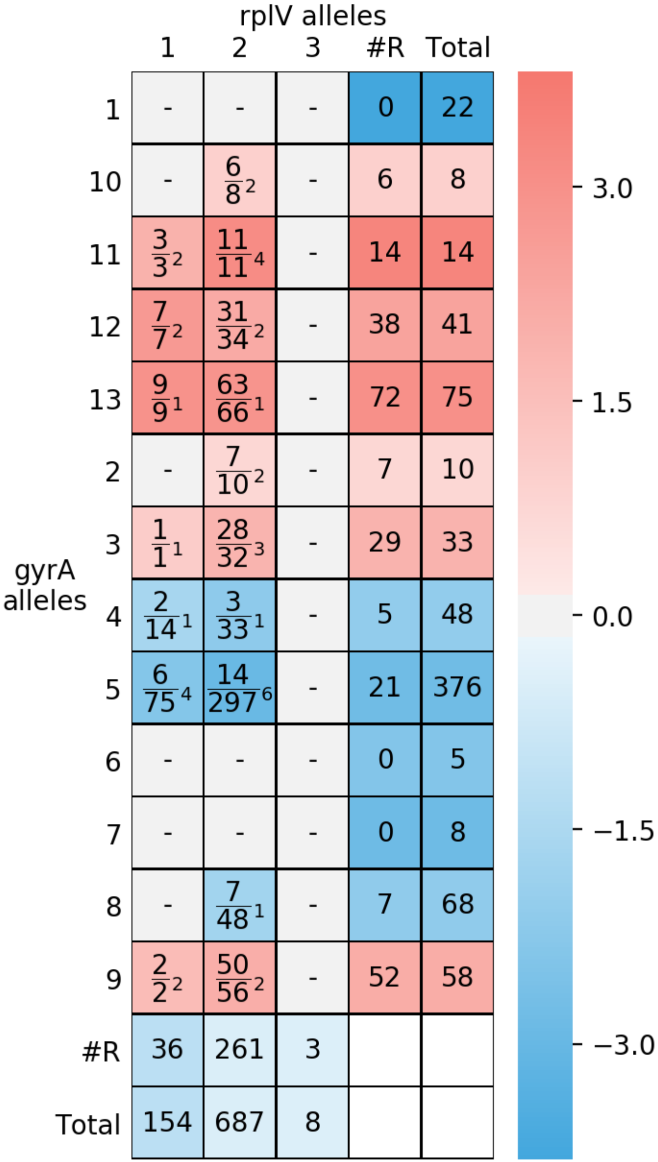
rplV alleles
1 2 3 #R Total
gyrA alleles
1 - - - 0 22
10 - 6/8 2 - 6 8
11 3/3 2 11/11 4 - 14 14
12 7/7 2 31/34 2 - 38 41
13 9/9 1 63/66 1 - 72 75
2 - 7/10 2 - 7 10
3 1/1 1 28/32 3 - 29 33
4 2/14 1 3/33 1 - 5 48
5 6/75 4 14/297 6 - 21 376
6 - - - 0 5
7 - - - 0 8
8 - 7/48 1 - 7 68
9 2/2 2 50/56 2 - 52 58
#R 36 261 3
Total 154 687 8
3.0
1.5
0.0
−1.5
−3.0

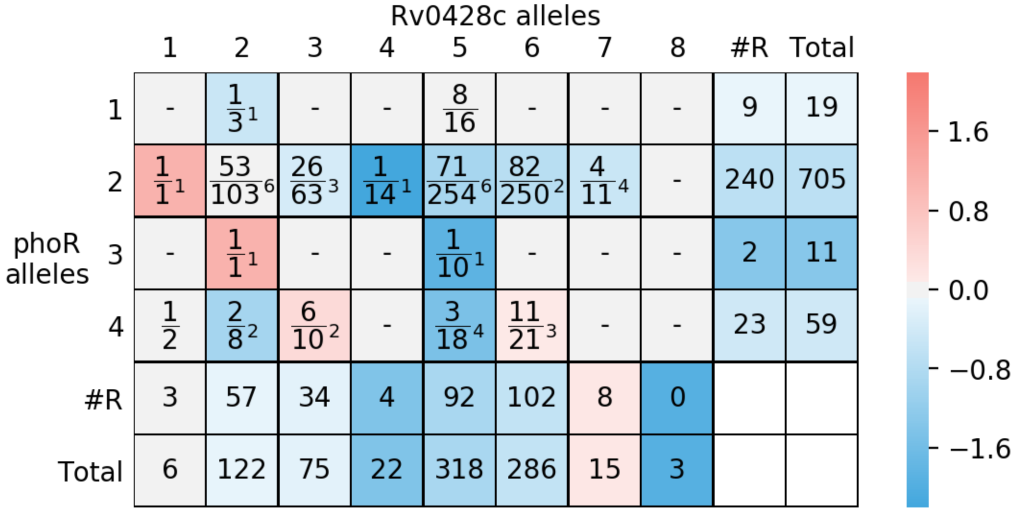

Rv0428c alleles
1 2 3 4 5 6 7 8 #R Total
phoR alleles
1: - | 1/3 1 | - | - | 8/16 | - | - | - | 9 | 19
2: 1/1 1 | 53/103 6 | 26/63 3 | 1/14 1 | 71/254 6 | 82/250 2 | 4/11 4 | - | 240 | 705
3: - | 1/1 1 | - | - | 1/10 1 | - | - | - | 2 | 11
4: 1/2 | 2/8 2 | 6/10 2 | - | 3/18 4 | 11/21 3 | - | - | 23 | 59
#R: 3 | 57 | 34 | 4 | 92 | 102 | 8 | 0
Total: 6 | 122 | 75 | 22 | 318 | 286 | 15 | 3
1.6
0.8
0.0
−0.8
−1.6

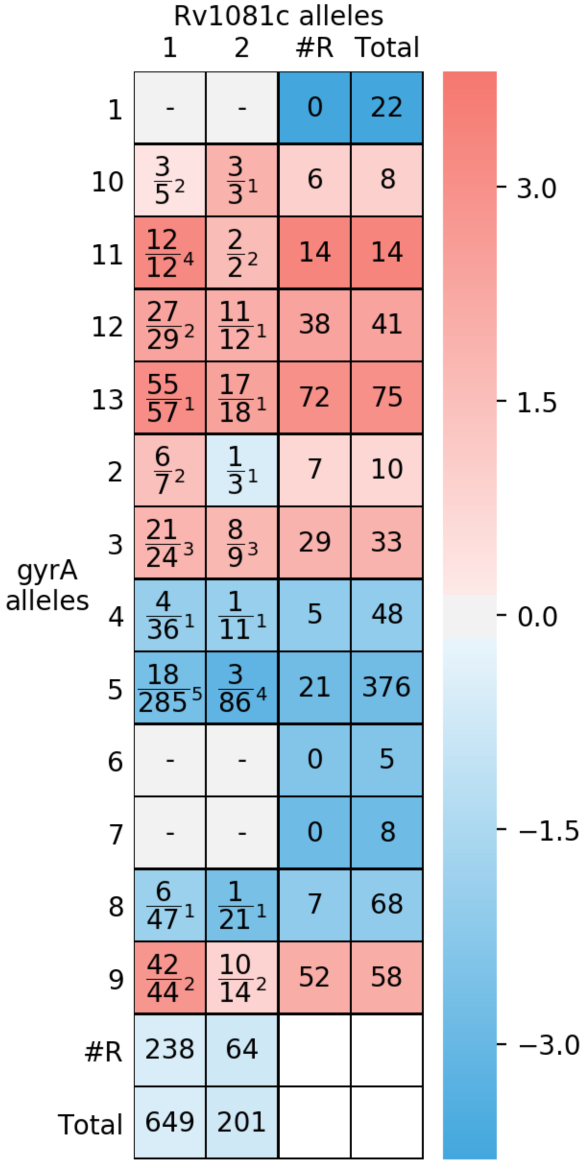
Rv1081c alleles
1
2
#R
Total
gyrA alleles
1
-
-
0
22
10
3/5 2
3/3 1
6
8
11
12/12 4
2/2 2
14
14
12
27/29 2
11/12 1
38
41
13
55/57 1
17/18 1
72
75
2
6/7 2
1/3 1
7
10
3
21/24 3
8/9 3
29
33
4
4/36 1
1/11 1
5
48
5
18/285 5
3/86 4
21
376
6
-
-
0
5
7
-
-
0
8
8
6/47 1
1/21 1
7
68
9
42/44 2
10/14 2
52
58
#R
238
64
Total
649
201
3.0
1.5
0.0
−1.5
−3.0

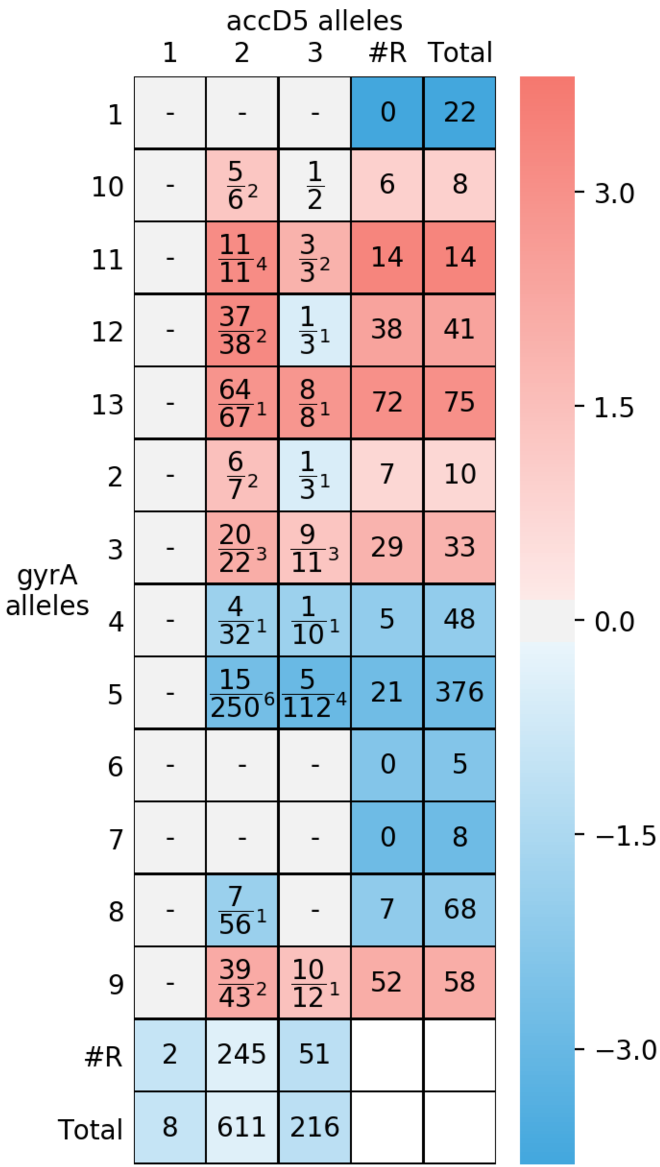
accD5 alleles
1
2
3
#R
Total
gyrA alleles
1 - - - 0 22
10 - 5/6 2 1/2 6 8
11 - 11/11 4 3/3 2 14 14
12 - 37/38 2 1/3 1 38 41
13 - 64/67 1 8/8 1 72 75
2 - 6/7 2 1/3 1 7 10
3 - 20/22 3 9/11 3 29 33
4 - 4/32 1 1/10 1 5 48
5 - 15/250 6 5/112 4 21 376
6 - - - 0 5
7 - - - 0 8
8 - 7/56 1 - 7 68
9 - 39/43 2 10/12 1 52 58
#R 2 245 51
Total 8 611 216
3.0
1.5
0.0
−1.5
−3.0

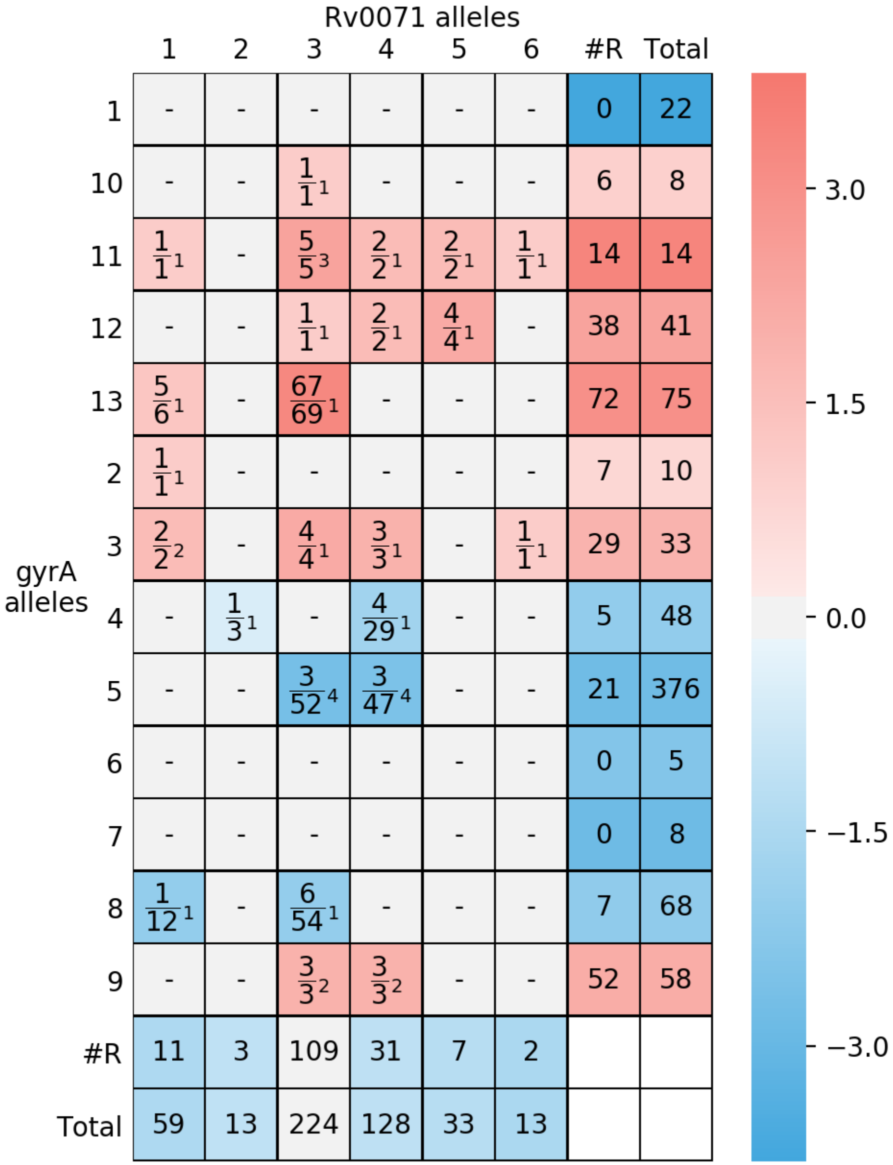

Rv0071 alleles
1
2
3
4
5
6
#R
Total
gyrA alleles
1
10
11
12
13
2
3
4
5
6
7
8
9
#R
Total
1: - - - - - - 0 22
10: - - 1/1 1 - - - 6 8
11: 1/1 1 - 5/5 3 2/2 1 2/2 1 1/1 1 14 14
12: - - 1/1 1 2/2 1 4/4 1 - 38 41
13: 5/6 1 - 67/69 1 - - - 72 75
2: 1/1 1 - - - - - 7 10
3: 2/2 2 - 4/4 1 3/3 1 - 1/1 1 29 33
4: - 1/3 1 - 4/29 1 - - 5 48
5: - - 3/52 4 3/47 4 - - 21 376
6: - - - - - - 0 5
7: - - - - - - 0 8
8: 1/12 1 - 6/54 1 - - - 7 68
9: - - 3/3 2 3/3 2 - - 52 58
#R: 11 3 109 31 7 2
Total: 59 13 224 128 33 13
3.0
1.5
0.0
−1.5
−3.0

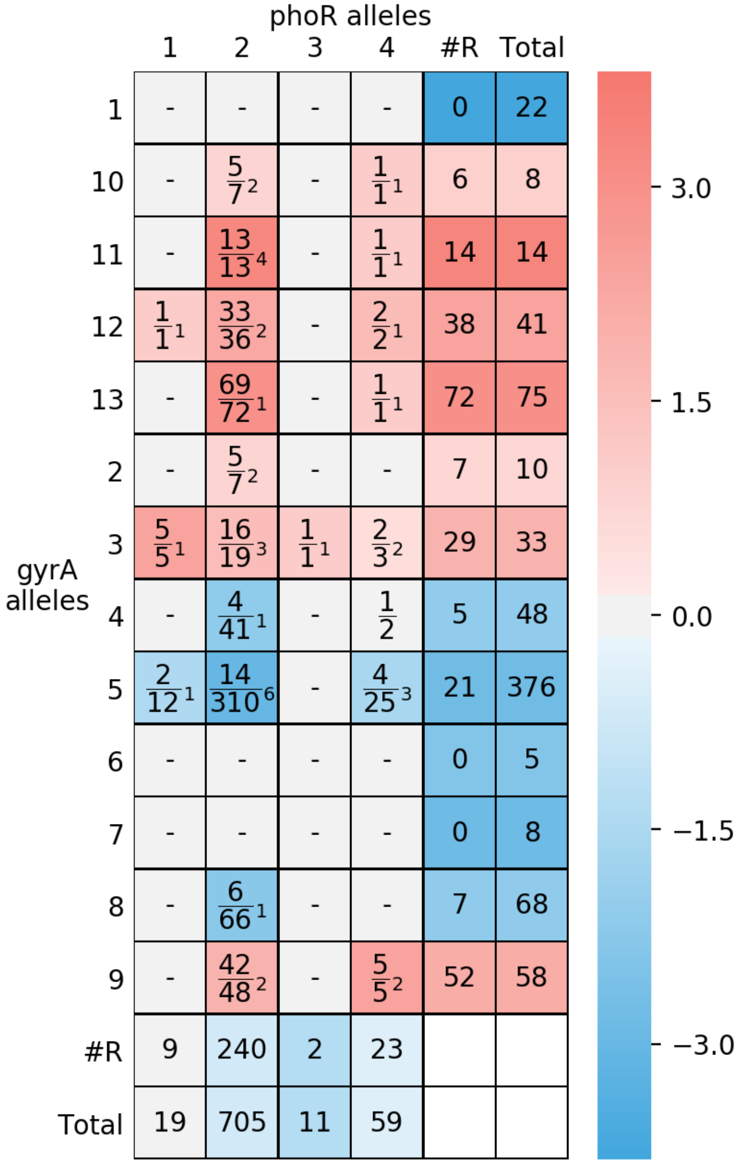
phoR alleles
1
2
3
4
#R
Total
gyrA alleles
1: - - - - 0 22
10: - 5/7 2 - 1/1 1 6 8
11: - 13/13 4 - 1/1 1 14 14
12: 1/1 1 33/36 2 - 2/2 1 38 41
13: - 69/72 1 - 1/1 1 72 75
2: - 5/7 2 - - 7 10
3: 5/5 1 16/19 3 1/1 1 2/3 2 29 33
4: - 4/41 1 - 1/2 5 48
5: 2/12 1 14/310 6 - 4/25 3 21 376
6: - - - - 0 5
7: - - - - 0 8
8: - 6/66 1 - - 7 68
9: - 42/48 2 - 5/5 2 52 58
#R: 9 240 2 23
Total: 19 705 11 59
3.0
1.5
0.0
−1.5
−3.0

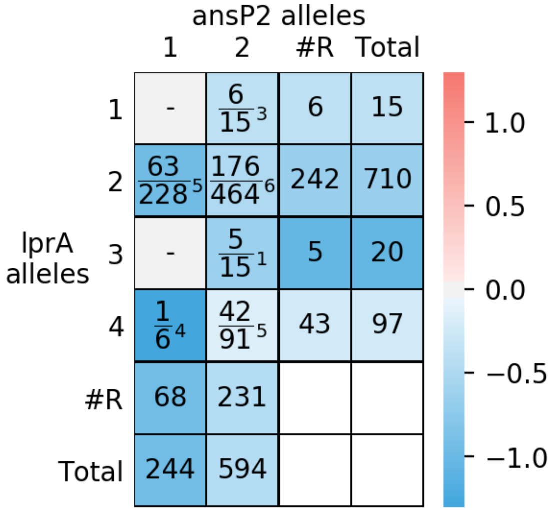
ansP2 alleles
1
2
#R
Total
lprA alleles
1
2
3
4
#R
Total
-
6/15 3
6
15
63/228 5
176/464 6
242
710
-
5/15 1
5
20
1/6 4
42/91 5
43
97
68
231
244
594
1.0
0.5
0.0
−0.5
−1.0

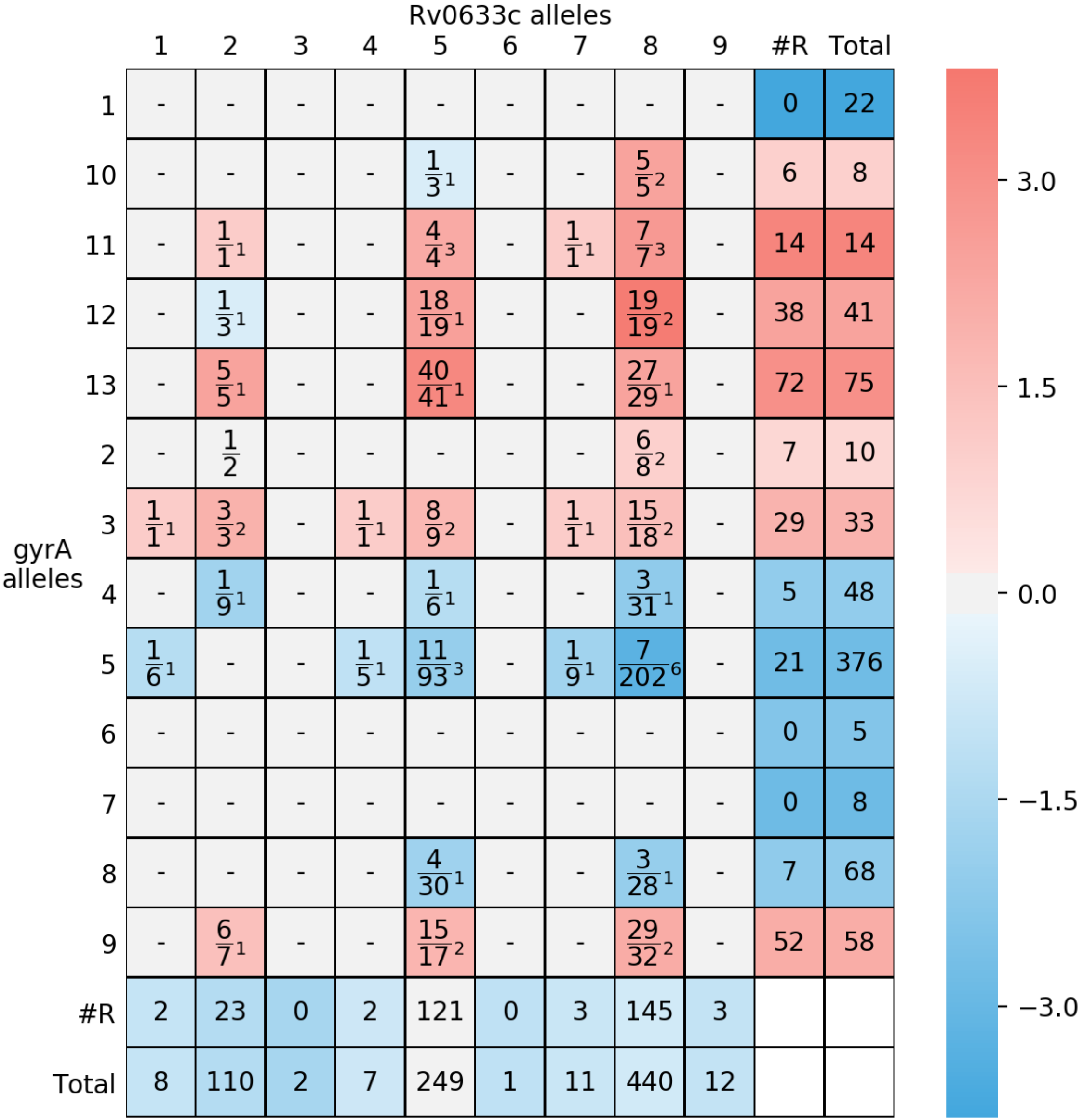

Rv0633c alleles
1 2 3 4 5 6 7 8 9 #R Total
gyrA alleles
1: - - - - - - - - - 0 22
10: - - - - 1/3 1 - - 5/5 2 - 6 8
11: - 1/1 1 - - 4/4 3 - 1/1 1 7/7 3 - 14 14
12: - 1/3 1 - - 18/19 1 - - 19/19 2 - 38 41
13: - 5/5 1 - - 40/41 1 - - 27/29 1 - 72 75
2: - 1/2 - - - - - 6/8 2 - 7 10
3: 1/1 1 3/3 2 - 1/1 1 8/9 2 - 1/1 1 15/18 2 - 29 33
4: - 1/9 1 - - 1/6 1 - - 3/31 1 - 5 48
5: 1/6 1 - - 1/5 1 11/93 3 - 1/9 1 7/202 6 - 21 376
6: - - - - - - - - - 0 5
7: - - - - - - - - - 0 8
8: - - - - 4/30 1 - - 3/28 1 - 7 68
9: - 6/7 1 - - 15/17 2 - - 29/32 2 - 52 58
#R: 2 23 0 2 121 0 3 145 3
Total: 8 110 2 7 249 1 11 440 12
3.0
1.5
0.0
−1.5
−3.0

Rv0428c alleles

Rv0071 alleles	1	2	3	4	5	6	7	8	#R	Total
1	-	$\frac{2}{9}^{2}$	-	$\frac{1}{3}^{1}$	$\frac{7}{44}^{5}$	$\frac{1}{2}$	-	-	11	59
2	-	-	-	-	-	-	$\frac{3}{3}^{1}$	-	3	13
3	-	$\frac{45}{69}^{4}$	-	$\frac{1}{3}^{1}$	$\frac{60}{142}^{4}$	-	$\frac{2}{4}$	-	109	224
4	$\frac{2}{4}$	$\frac{7}{25}^{4}$	$\frac{1}{1}^{1}$	-	$\frac{17}{76}^{3}$	$\frac{1}{6}^{3}$	$\frac{3}{6}$	-	31	128
5	-	$\frac{2}{2}^{1}$	-	-	$\frac{5}{23}^{3}$	-	-	-	7	33
6	$\frac{1}{1}^{1}$	-	-	-	$\frac{1}{7}^{2}$	-	-	-	2	13
#R	3	57	34	4	92	102	8	0		
Total	6	122	75	22	318	286	15	3		

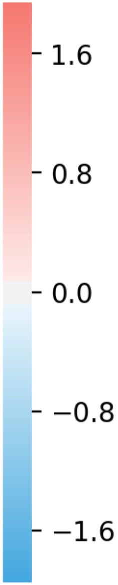

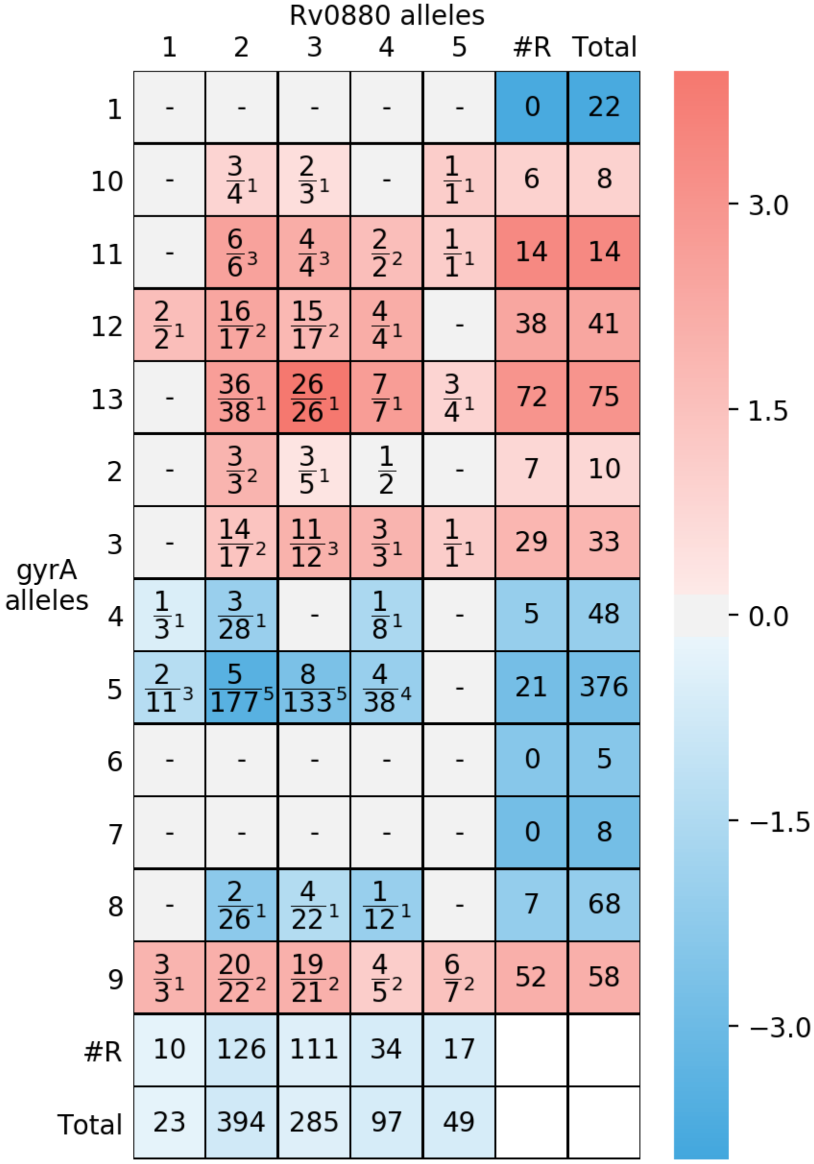

Rv0880 alleles
1 2 3 4 5 #R Total
gyrA alleles
1 - - - - - 0 22
10 - 3/4 1 2/3 1 - 1/1 1 6 8
11 - 6/6 3 4/4 3 2/2 2 1/1 1 14 14
12 2/2 1 16/17 2 15/17 2 4/4 1 - 38 41
13 - 36/38 1 26/26 1 7/7 1 3/4 1 72 75
2 - 3/3 2 3/5 1 1/2 - 7 10
3 - 14/17 2 11/12 3 3/3 1 1/1 1 29 33
4 1/3 1 3/28 1 - 1/8 1 - 5 48
5 2/11 3 5/177 5 8/133 5 4/38 4 - 21 376
6 - - - - - 0 5
7 - - - - - 0 8
8 - 2/26 1 4/22 1 1/12 1 - 7 68
9 3/3 1 20/22 2 19/21 2 4/5 2 6/7 2 52 58
#R 10 126 111 34 17
Total 23 394 285 97 49
3.0
1.5
0.0
−1.5
−3.0
