# Supplementary material for: Machine learning and structural analysis of Mycobacterium tuberculosis pan-genome identifies genetic signatures of antibiotic resistance
Source: Nat Commun. 2018 Oct 17;9:4306. doi: 10.1038/s41467-018-06634-y (PMC6193043; doi:10.1038/s41467-018-06634-y)
Supplement: Supplementary file 8 — Supplementary Data 5 [file 41467_2018_6634_MOESM8_ESM.zip › Supplementary Data 5/streptomycin_epistasis.pdf]

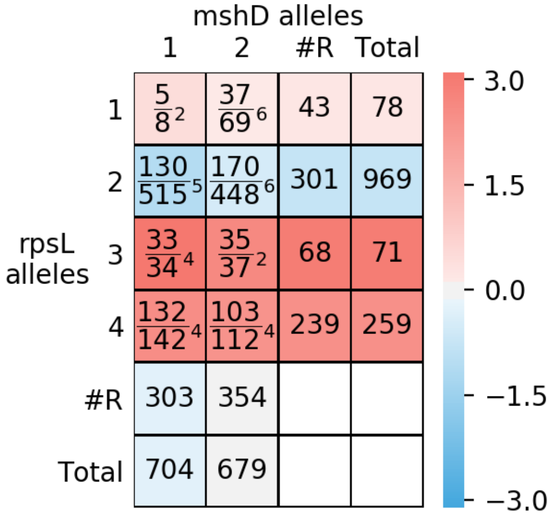
mshD alleles
1
2
#R
Total
rpsL alleles
1
2
3
4
#R
Total
5/8 2
37/69 6
43
78
130/515 5
170/448 6
301
969
33/34 4
35/37 2
68
71
132/142 4
103/112 4
239
259
303
354
704
679
3.0
1.5
0.0
−1.5
−3.0

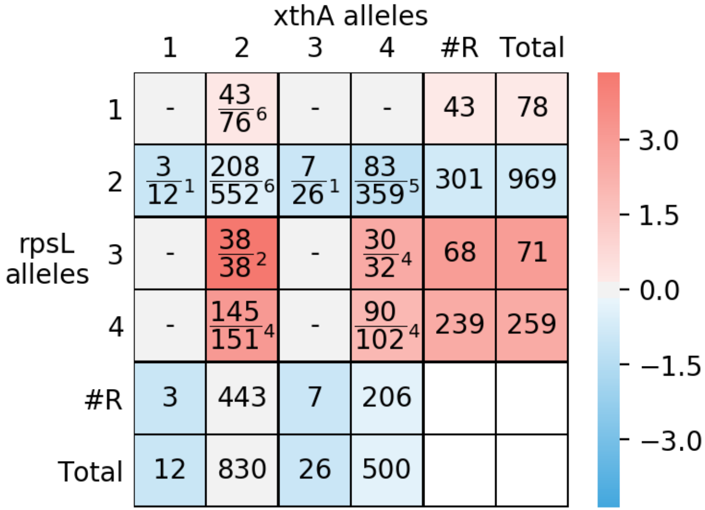

xthA alleles
1
2
3
4
#R
Total
rpsL alleles
1
2
3
4
#R
Total
-
43/76 6
-
-
43
78
3/12 1
208/552 6
7/26 1
83/359 5
301
969
-
38/38 2
-
30/32 4
68
71
-
145/151 4
-
90/102 4
239
259
3
443
7
206
12
830
26
500
3.0
1.5
0.0
−1.5
−3.0

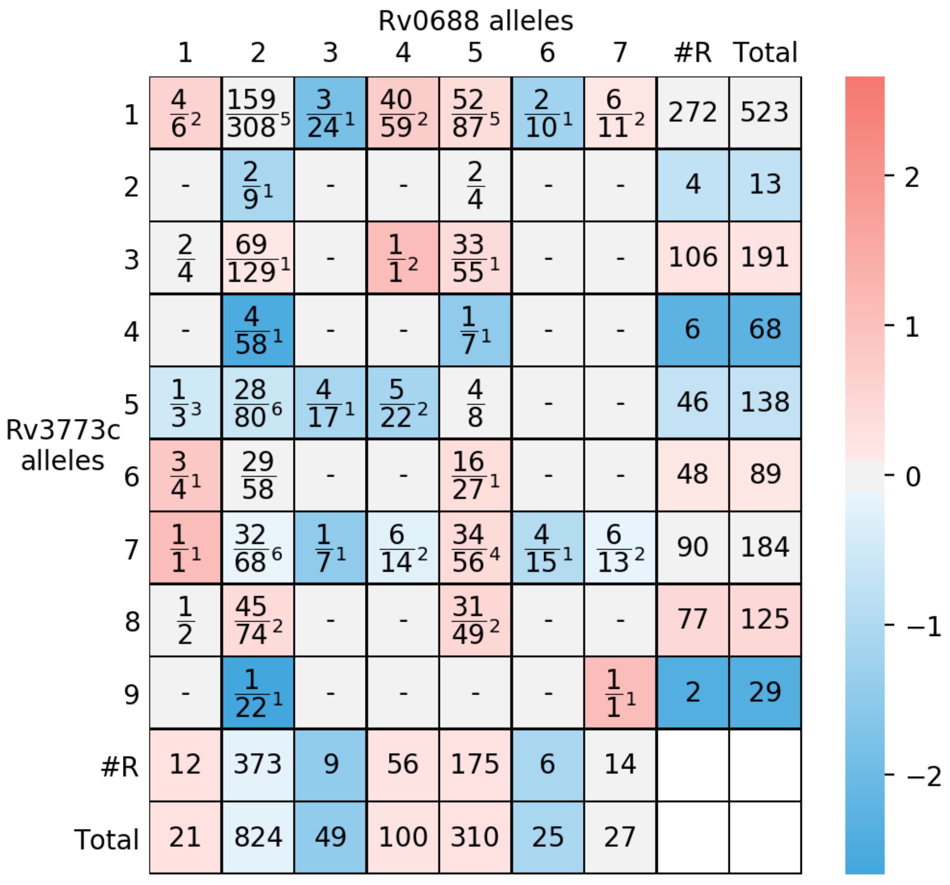
Rv0688 alleles
1 2 3 4 5 6 7 #R Total
Rv3773c alleles
1 4/6 2 | 159/308 5 | 3/24 1 | 40/59 2 | 52/87 5 | 2/10 1 | 6/11 2 | 272 | 523
2 - | 2/9 1 | - | - | 2/4 | - | - | 4 | 13
3 2/4 | 69/129 1 | - | 1/1 2 | 33/55 1 | - | - | 106 | 191
4 - | 4/58 1 | - | - | 1/7 1 | - | - | 6 | 68
5 1/3 3 | 28/80 6 | 4/17 1 | 5/22 2 | 4/8 | - | - | 46 | 138
6 3/4 1 | 29/58 | - | - | 16/27 1 | - | - | 48 | 89
7 1/1 1 | 32/68 6 | 1/7 1 | 6/14 2 | 34/56 4 | 4/15 1 | 6/13 2 | 90 | 184
8 1/2 | 45/74 2 | - | - | 31/49 2 | - | - | 77 | 125
9 - | 1/22 1 | - | - | - | - | 1/1 1 | 2 | 29
#R 12 | 373 | 9 | 56 | 175 | 6 | 14
Total 21 | 824 | 49 | 100 | 310 | 25 | 27
2
1
0
−1
−2

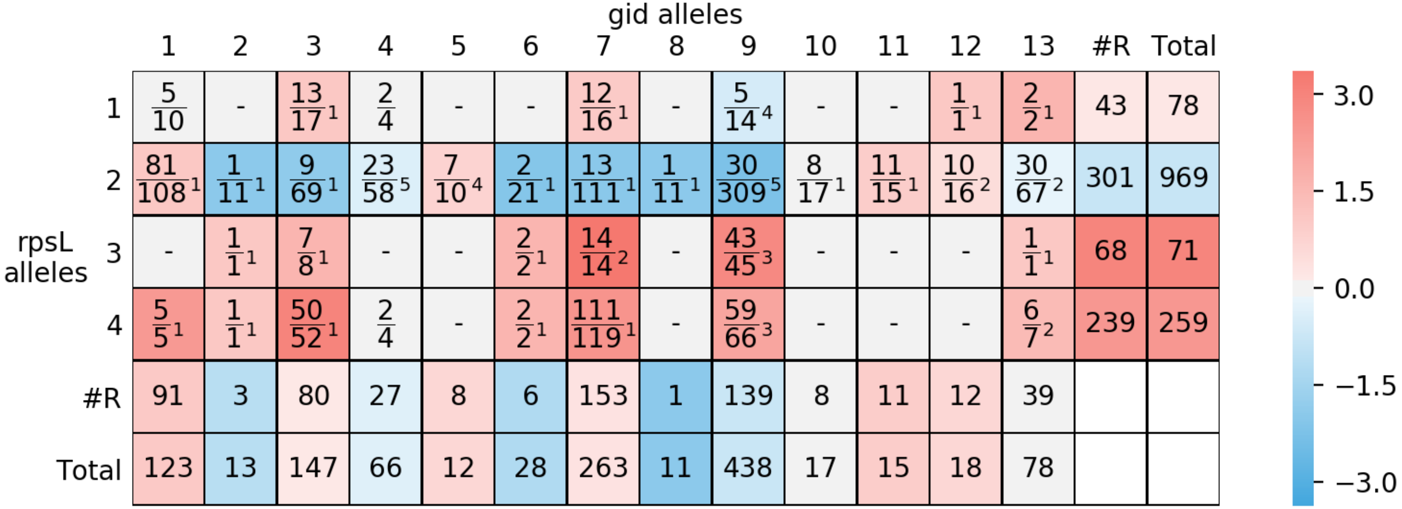
gid alleles
1 2 3 4 5 6 7 8 9 10 11 12 13 #R Total
rpsL alleles
1: 5/10 | - | 13/17 1 | 2/4 | - | - | 12/16 1 | - | 5/14 4 | - | - | 1/1 1 | 2/2 1 | 43 | 78
2: 81/108 1 | 1/11 1 | 9/69 1 | 23/58 5 | 7/10 4 | 2/21 1 | 13/111 1 | 1/11 1 | 30/309 5 | 8/17 1 | 11/15 1 | 10/16 2 | 30/67 2 | 301 | 969
3: - | 1/1 1 | 7/8 1 | - | - | 2/2 1 | 14/14 2 | - | 43/45 3 | - | - | - | 1/1 1 | 68 | 71
4: 5/5 1 | 1/1 1 | 50/52 1 | 2/4 | - | 2/2 1 | 111/119 1 | - | 59/66 3 | - | - | - | 6/7 2 | 239 | 259
#R: 91 | 3 | 80 | 27 | 8 | 6 | 153 | 1 | 139 | 8 | 11 | 12 | 39
Total: 123 | 13 | 147 | 66 | 12 | 28 | 263 | 11 | 438 | 17 | 15 | 18 | 78
3.0
1.5
0.0
−1.5
−3.0

Rv1591 alleles

yajC alleles	1	2	3	4	5	6	7	8	9	10	11	12	13	#R	Total
1	-	-	6/6 [1]	-	-	18/18 [1]	-	-	1/1 [1]	-	-	2/2 [1]	-	30	30
2	38/52 [1]	7/18 [1]	177/352 [6]	13/87 [2]	8/16	141/354 [6]	1/32 [2]	8/12 [1]	53/104 [6]	3/25 [2]	38/53 [1]	33/54 [3]	1/12 [1]	543	1209
3	5/6 [1]	1/1 [1]	22/38 [5]	3/10 [1]	2/2 [1]	32/59 [4]	-	-	4/9 [2]	-	1/1 [1]	6/8 [3]	-	84	147
#R	43	8	207	16	10	195	1	8	58	3	39	41	1		
Total	58	19	399	97	18	436	35	12	114	26	54	64	12		

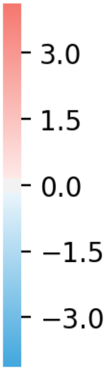

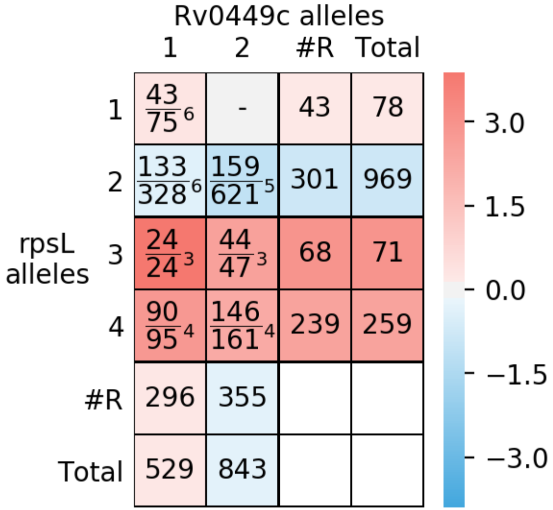
Rv0449c alleles
1
2
#R
Total
rpsL alleles
1
2
3
4
#R
Total
43/75 6
-
43
78
133/328 6
159/621 5
301
969
24/24 3
44/47 3
68
71
90/95 4
146/161 4
239
259
296
355
529
843
3.0
1.5
0.0
−1.5
−3.0

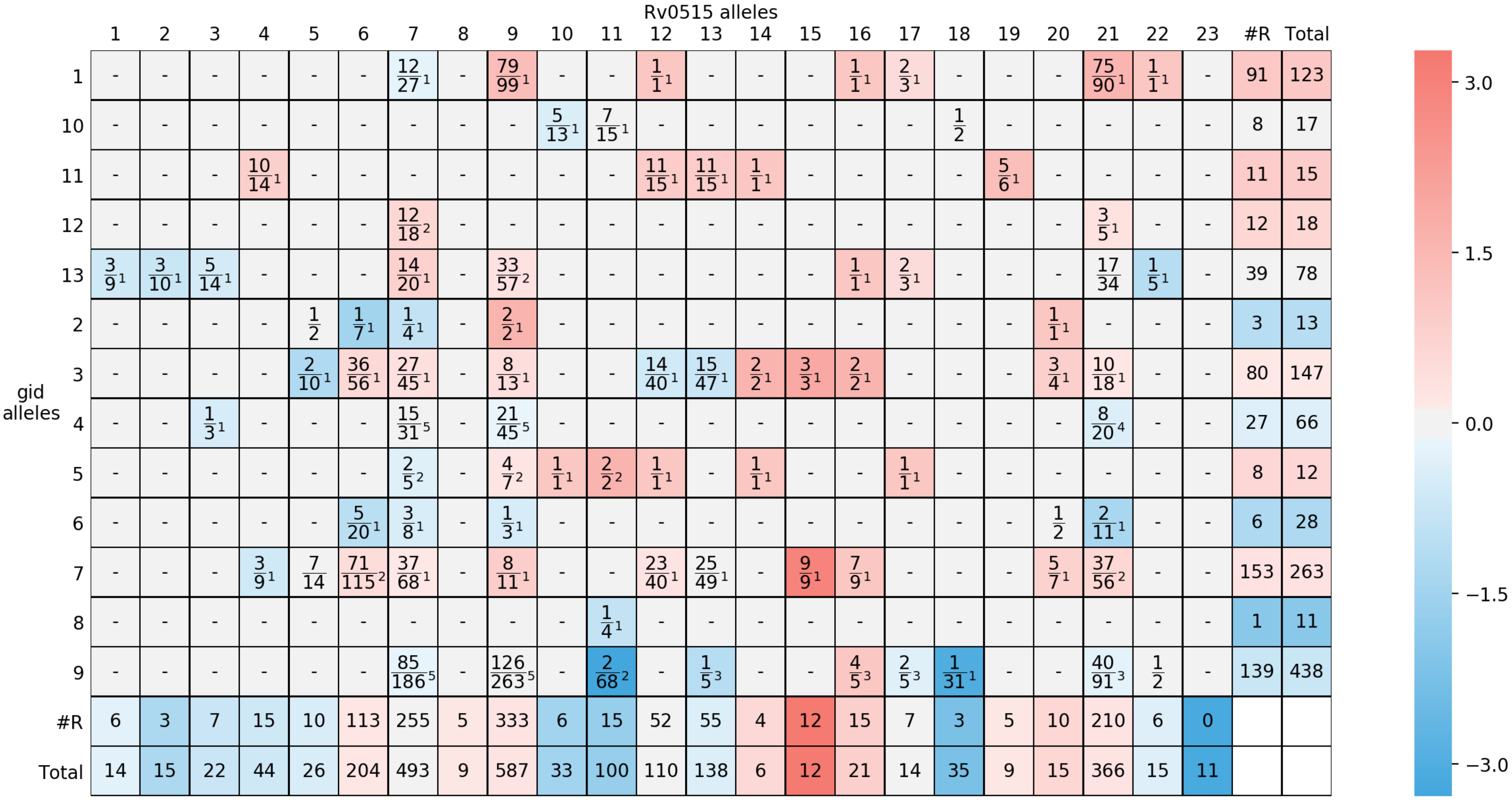

gid alleles \ Rv0515 alleles	1	2	3	4	5	6	7	8	9	10	11	12	13	14	15	16	17	18	19	20	21	22	23	#R	Total
1	-	-	-	-	-	-	$\frac{12}{27}^{1}$	-	$\frac{79}{99}^{1}$	-	-	$\frac{1}{1}^{1}$	-	-	-	$\frac{1}{1}^{1}$	$\frac{2}{3}^{1}$	-	-	-	$\frac{75}{90}^{1}$	$\frac{1}{1}^{1}$	-	91	123
10	-	-	-	-	-	-	-	-	-	$\frac{5}{13}^{1}$	$\frac{7}{15}^{1}$	-	-	-	-	-	-	$\frac{1}{2}$	-	-	-	-	-	8	17
11	-	-	-	$\frac{10}{14}^{1}$	-	-	-	-	-	-	-	$\frac{11}{15}^{1}$	$\frac{11}{15}^{1}$	$\frac{1}{1}^{1}$	-	-	-	-	$\frac{5}{6}^{1}$	-	-	-	-	11	15
12	-	-	-	-	-	-	$\frac{12}{18}^{2}$	-	-	-	-	-	-	-	-	-	-	-	-	-	$\frac{3}{5}^{1}$	-	-	12	18
13	$\frac{3}{9}^{1}$	$\frac{3}{10}^{1}$	$\frac{5}{14}^{1}$	-	-	-	$\frac{14}{20}^{1}$	-	$\frac{33}{57}^{2}$	-	-	-	-	-	-	$\frac{1}{1}^{1}$	$\frac{2}{3}^{1}$	-	-	-	$\frac{17}{34}$	$\frac{1}{5}^{1}$	-	39	78
2	-	-	-	-	$\frac{1}{2}$	$\frac{1}{7}^{1}$	$\frac{1}{4}^{1}$	-	$\frac{2}{2}^{1}$	-	-	-	-	-	-	-	-	-	-	$\frac{1}{1}^{1}$	-	-	-	3	13
3	-	-	-	-	$\frac{2}{10}^{1}$	$\frac{36}{56}^{1}$	$\frac{27}{45}^{1}$	-	$\frac{8}{13}^{1}$	-	-	$\frac{14}{40}^{1}$	$\frac{15}{47}^{1}$	$\frac{2}{2}^{1}$	$\frac{3}{3}^{1}$	$\frac{2}{2}^{1}$	-	-	-	$\frac{3}{4}^{1}$	$\frac{10}{18}^{1}$	-	-	80	147
4	-	-	$\frac{1}{3}^{1}$	-	-	-	$\frac{15}{31}^{5}$	-	$\frac{21}{45}^{5}$	-	-	-	-	-	-	-	-	-	-	-	$\frac{8}{20}^{4}$	-	-	27	66
5	-	-	-	-	-	-	$\frac{2}{5}^{2}$	-	$\frac{4}{7}^{2}$	$\frac{1}{1}^{1}$	$\frac{2}{2}^{2}$	$\frac{1}{1}^{1}$	-	$\frac{1}{1}^{1}$	-	-	$\frac{1}{1}^{1}$	-	-	-	-	-	-	8	12
6	-	-	-	-	-	$\frac{5}{20}^{1}$	$\frac{3}{8}^{1}$	-	$\frac{1}{3}^{1}$	-	-	-	-	-	-	-	-	-	-	$\frac{1}{2}$	$\frac{2}{11}^{1}$	-	-	6	28
7	-	-	-	$\frac{3}{9}^{1}$	$\frac{7}{14}$	$\frac{71}{115}^{2}$	$\frac{37}{68}^{1}$	-	$\frac{8}{11}^{1}$	-	-	$\frac{23}{40}^{1}$	$\frac{25}{49}^{1}$	-	$\frac{9}{9}^{1}$	$\frac{7}{9}^{1}$	-	-	-	$\frac{5}{7}^{1}$	$\frac{37}{56}^{2}$	-	-	153	263
8	-	-	-	-	-	-	-	-	-	-	$\frac{1}{4}^{1}$	-	-	-	-	-	-	-	-	-	-	-	-	1	11
9	-	-	-	-	-	-	$\frac{85}{186}^{5}$	-	$\frac{126}{263}^{5}$	-	$\frac{2}{68}^{2}$	-	$\frac{1}{5}^{3}$	-	-	$\frac{4}{5}^{3}$	$\frac{2}{5}^{3}$	$\frac{1}{31}^{1}$	-	-	$\frac{40}{91}^{3}$	$\frac{1}{2}$	-	139	438
#R	6	3	7	15	10	113	255	5	333	6	15	52	55	4	12	15	7	3	5	10	210	6	0		
Total	14	15	22	44	26	204	493	9	587	33	100	110	138	6	12	21	14	35	9	15	366	15	11		

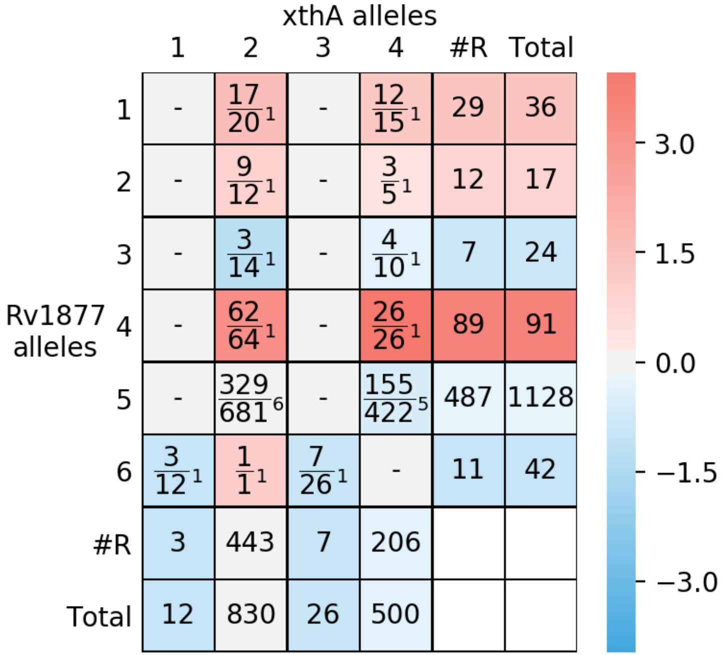
xthA alleles
1
2
3
4
#R
Total
Rv1877 alleles
1
2
3
4
5
6
#R
Total
-
17/20 1
-
12/15 1
29
36
-
9/12 1
-
3/5 1
12
17
-
3/14 1
-
4/10 1
7
24
-
62/64 1
-
26/26 1
89
91
-
329/681 6
-
155/422 5
487
1128
3/12 1
1/1 1
7/26 1
-
11
42
3
443
7
206
12
830
26
500
3.0
1.5
0.0
−1.5
−3.0

moeW alleles

yajC alleles	1	2	3	4	5	#R	Total
1	$\frac{7}{7}^{1}$	$\frac{20}{20}^{1}$	$\frac{2}{2}^{1}$	-	$\frac{1}{1}^{1}$	30	30
2	$\frac{318}{591}^{6}$	$\frac{50}{224}^{4}$	$\frac{62}{191}^{5}$	$\frac{6}{15}^{4}$	$\frac{81}{136}^{5}$	543	1209
3	$\frac{40}{65}^{5}$	$\frac{19}{40}^{4}$	$\frac{9}{19}^{4}$	$\frac{1}{2}$	$\frac{13}{18}^{4}$	84	147
#R	368	92	73	7	95		
Total	667	288	213	17	155		

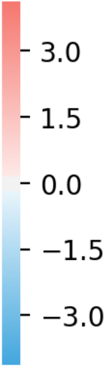

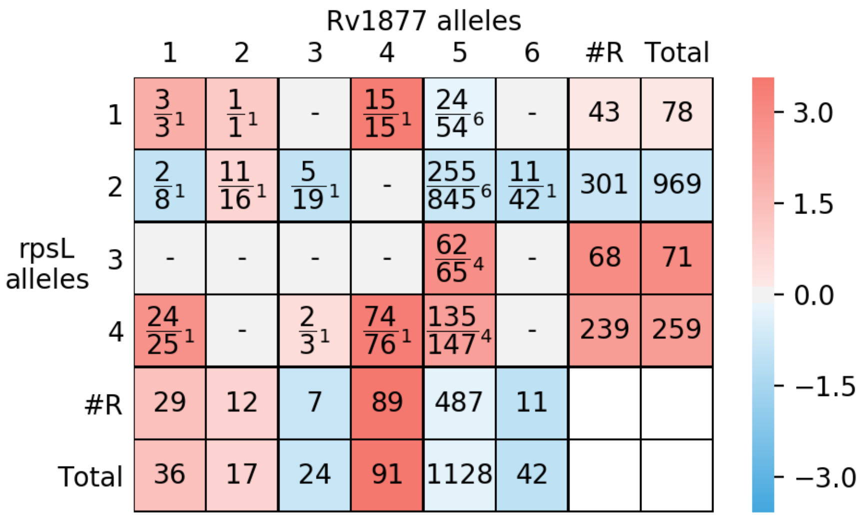

Rv1877 alleles
1
2
3
4
5
6
#R
Total
rpsL alleles
1
3/3 1
1/1 1
-
15/15 1
24/54 6
-
43
78
2
2/8 1
11/16 1
5/19 1
-
255/845 6
11/42 1
301
969
3
-
-
-
-
62/65 4
-
68
71
4
24/25 1
-
2/3 1
74/76 1
135/147 4
-
239
259
#R
29
12
7
89
487
11
Total
36
17
24
91
1128
42
3.0
1.5
0.0
−1.5
−3.0

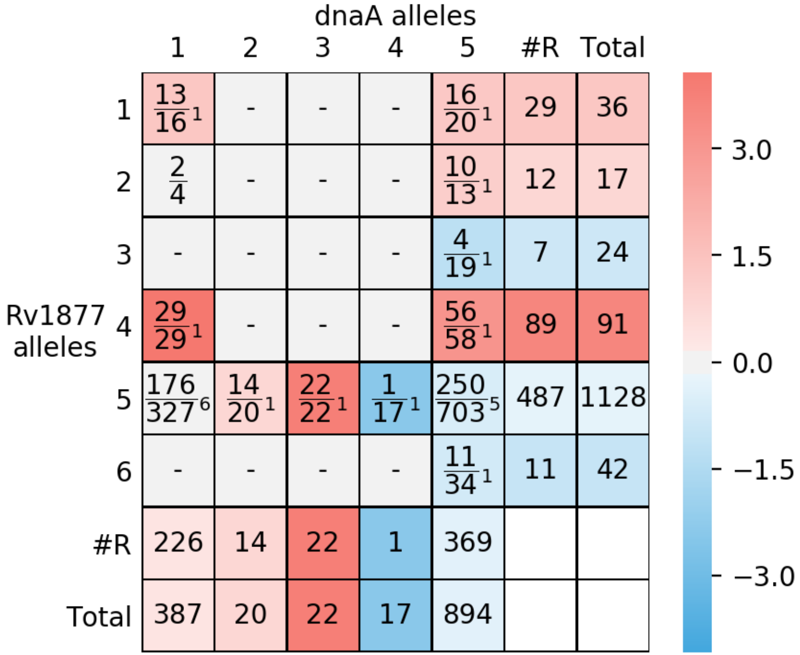
dnaA alleles
1
2
3
4
5
#R
Total
Rv1877 alleles
1
13/16 1
-
-
-
16/20 1
29
36
2
2/4
-
-
-
10/13 1
12
17
3
-
-
-
-
4/19 1
7
24
4
29/29 1
-
-
-
56/58 1
89
91
5
176/327 6
14/20 1
22/22 1
1/17 1
250/703 5
487
1128
6
-
-
-
-
11/34 1
11
42
#R
226
14
22
1
369
Total
387
20
22
17
894
3.0
1.5
0.0
−1.5
−3.0

Rv1591 alleles

rpsL alleles	1	2	3	4	5	6	7	8	9	10	11	12	13	#R	Total
1	$\frac{2}{6}^{1}$	$\frac{1}{1}^{1}$	$\frac{16}{30}^{4}$	-	$\frac{5}{7}^{2}$	$\frac{9}{17}^{4}$	-	$\frac{2}{2}^{1}$	$\frac{5}{8}^{3}$	-	$\frac{1}{1}^{1}$	$\frac{1}{2}$	-	43	78
2	$\frac{39}{50}^{1}$	$\frac{6}{17}^{1}$	$\frac{91}{262}^{5}$	$\frac{15}{94}^{2}$	$\frac{2}{8}^{2}$	$\frac{53}{277}^{6}$	$\frac{1}{34}^{2}$	$\frac{6}{10}^{1}$	$\frac{24}{73}^{6}$	$\frac{3}{24}^{2}$	$\frac{36}{51}^{1}$	$\frac{9}{30}^{3}$	$\frac{1}{11}^{1}$	301	969
3	-	-	$\frac{22}{23}^{3}$	-	-	$\frac{36}{36}^{3}$	-	-	$\frac{9}{9}^{2}$	-	-	$\frac{1}{2}$	-	68	71
4	$\frac{2}{2}^{1}$	-	$\frac{75}{80}^{2}$	-	-	$\frac{94}{100}^{4}$	-	-	$\frac{19}{22}^{3}$	-	$\frac{2}{2}^{1}$	$\frac{30}{30}^{1}$	-	239	259
#R	43	8	207	16	10	195	1	8	58	3	39	41	1		
Total	58	19	399	97	18	436	35	12	114	26	54	64	12		

Color scale: 3.0, 1.5, 0.0, −1.5, −3.0
