# Supplementary material for: Machine learning and structural analysis of Mycobacterium tuberculosis pan-genome identifies genetic signatures of antibiotic resistance
Source: Nat Commun. 2018 Oct 17;9:4306. doi: 10.1038/s41467-018-06634-y (PMC6193043; doi:10.1038/s41467-018-06634-y)
Supplement: Supplementary file 8 — Supplementary Data 5 [file 41467_2018_6634_MOESM8_ESM.zip › Supplementary Data 5/XDR_epistasis.pdf]

gyrA alleles \ katG alleles	1	2	3	4	5	6	7	8	#R	Total
1	-	-	-	-	-	-	-	-	0	24
10	-	-	$\frac{1}{6}{}^{1}$	-	-	-	$\frac{1}{5}{}^{1}$	-	4	16
11	-	-	-	-	-	-	$\frac{2}{2}{}^{1}$	-	2	18
12	-	-	$\frac{8}{10}{}^{1}$	$\frac{3}{6}$	-	-	$\frac{11}{23}{}^{1}$	$\frac{1}{2}$	25	48
13	-	-	-	$\frac{56}{64}{}^{1}$	-	-	-	$\frac{11}{15}{}^{1}$	68	80
2	-	-	$\frac{1}{2}$	$\frac{1}{1}{}^{1}$	-	-	$\frac{1}{4}{}^{1}$	-	5	10
3	-	-	$\frac{6}{8}{}^{1}$	-	$\frac{1}{2}$	-	$\frac{4}{10}{}^{1}$	$\frac{4}{4}{}^{1}$	15	35
4	-	-	-	-	-	-	-	$\frac{2}{17}{}^{1}$	2	88
5	-	$\frac{1}{87}{}^{6}$	-	$\frac{1}{109}{}^{4}$	-	$\frac{1}{24}{}^{4}$	$\frac{4}{113}{}^{3}$	-	8	769
6	-	-	-	-	-	-	-	-	0	22
7	-	-	-	-	-	-	-	-	0	140
8	-	-	-	$\frac{4}{47}{}^{1}$	-	-	-	$\frac{1}{31}{}^{1}$	5	95
9	-	-	$\frac{13}{21}{}^{1}$	$\frac{2}{11}{}^{2}$	-	-	$\frac{15}{23}{}^{1}$	-	32	71
#R	0	1	31	71	2	1	44	24		
Total	159	145	175	313	210	47	205	163		

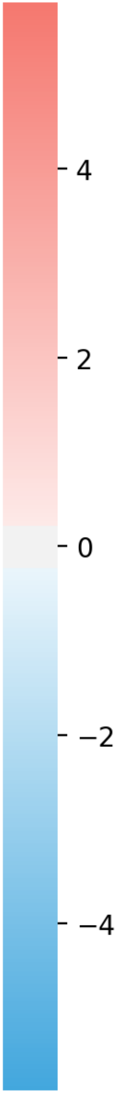

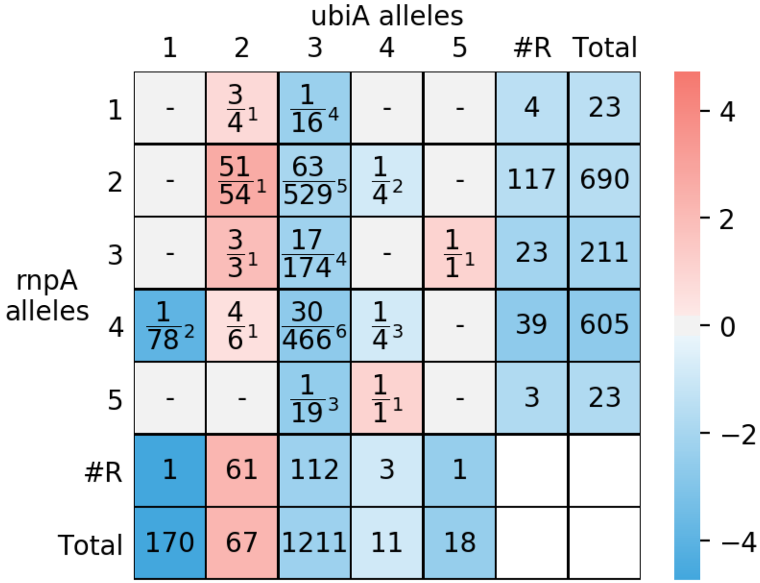
ubiA alleles
1
2
3
4
5
#R
Total
rnpA alleles
1
2
3
4
5
#R
Total
-
3/4 1
1/16 4
-
-
4
23
-
51/54 1
63/529 5
1/4 2
-
117
690
-
3/3 1
17/174 4
-
1/1 1
23
211
1/78 2
4/6 1
30/466 6
1/4 3
-
39
605
-
-
1/19 3
1/1 1
-
3
23
1
61
112
3
1
170
67
1211
11
18
4
2
0
−2
−4

pncA alleles (columns) × rpoB alleles (rows)

rpoB alleles	1	2	3	4	5	6	7	8	9	10	11	12	13	14	#R	Total
1	-	-	-	-	-	-	-	-	-	-	-	-	-	$\frac{10}{20}$	10	39
10	-	$\frac{1}{3}^{2}$	-	-	-	-	-	-	-	-	-	$\frac{1}{17}^{1}$	-	-	7	112
11	-	-	-	-	-	-	-	-	-	-	-	-	-	-	1	17
2	-	-	-	-	-	-	-	-	$\frac{5}{21}^{1}$	-	-	-	-	-	6	22
3	-	-	$\frac{3}{38}^{1}$	-	$\frac{12}{23}^{1}$	$\frac{4}{8}$	-	$\frac{8}{13}^{2}$	-	$\frac{3}{114}^{5}$	$\frac{9}{11}^{1}$	-	-	-	74	466
4	-	-	-	-	-	-	-	-	-	$\frac{2}{490}^{6}$	-	-	-	-	3	549
5	-	-	-	-	-	-	-	-	-	-	-	-	-	-	0	21
6	-	-	-	-	-	-	-	-	-	-	-	-	-	-	0	11
7	-	-	-	-	-	-	-	-	-	$\frac{1}{8}^{3}$	-	-	-	-	1	16
8	-	-	-	-	-	-	-	-	-	-	-	-	-	-	2	21
9	-	-	-	-	-	-	-	-	-	-	-	-	-	-	60	64
#R	0	1	3	0	12	4	0	8	5	9	9	1	0	10		
Total	11	11	38	17	24	16	15	15	22	740	11	22	16	21		

Colour scale: 4, 2, 0, −2, −4

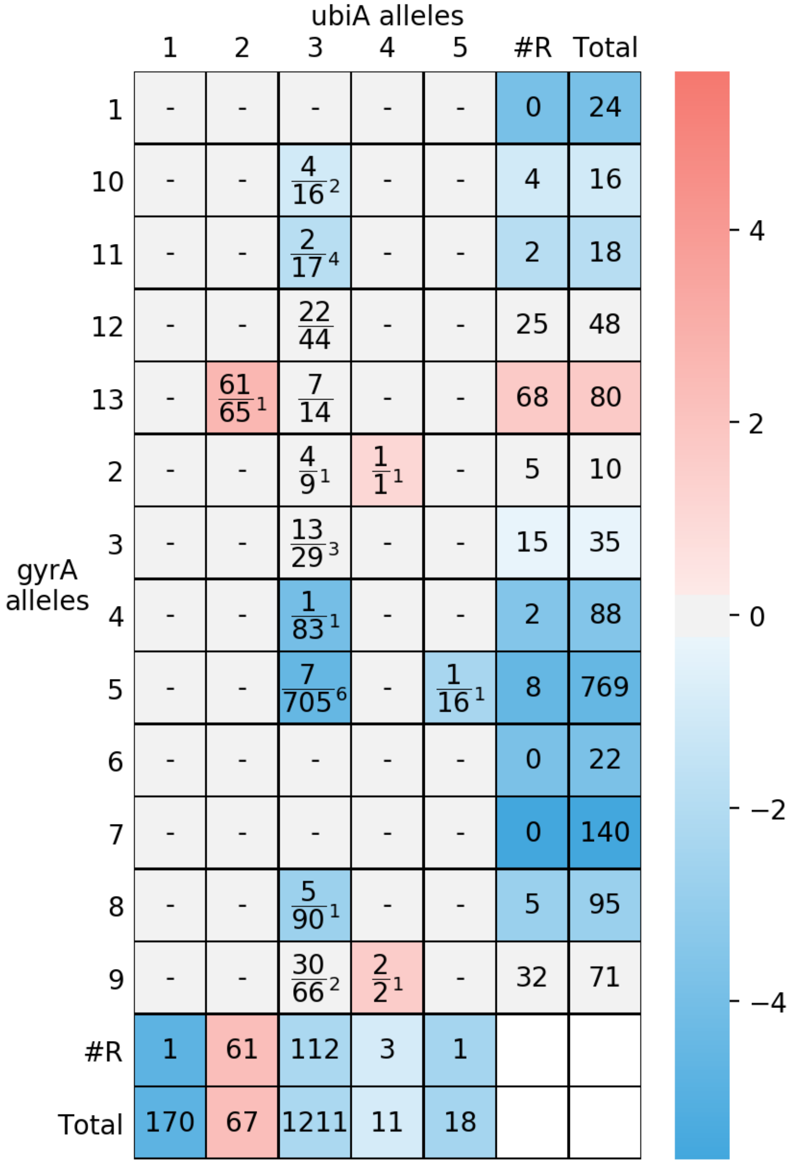

ubiA alleles
1 2 3 4 5 #R Total
gyrA alleles
1 - - - - - 0 24
10 - - 4/16 2 - - 4 16
11 - - 2/17 4 - - 2 18
12 - - 22/44 - - 25 48
13 - 61/65 1 7/14 - - 68 80
2 - - 4/9 1 1/1 1 - 5 10
3 - - 13/29 3 - - 15 35
4 - - 1/83 1 - - 2 88
5 - - 7/705 6 - 1/16 1 8 769
6 - - - - - 0 22
7 - - - - - 0 140
8 - - 5/90 1 - - 5 95
9 - - 30/66 2 2/2 1 - 32 71
#R 1 61 112 3 1
Total 170 67 1211 11 18
4
2
0
−2
−4

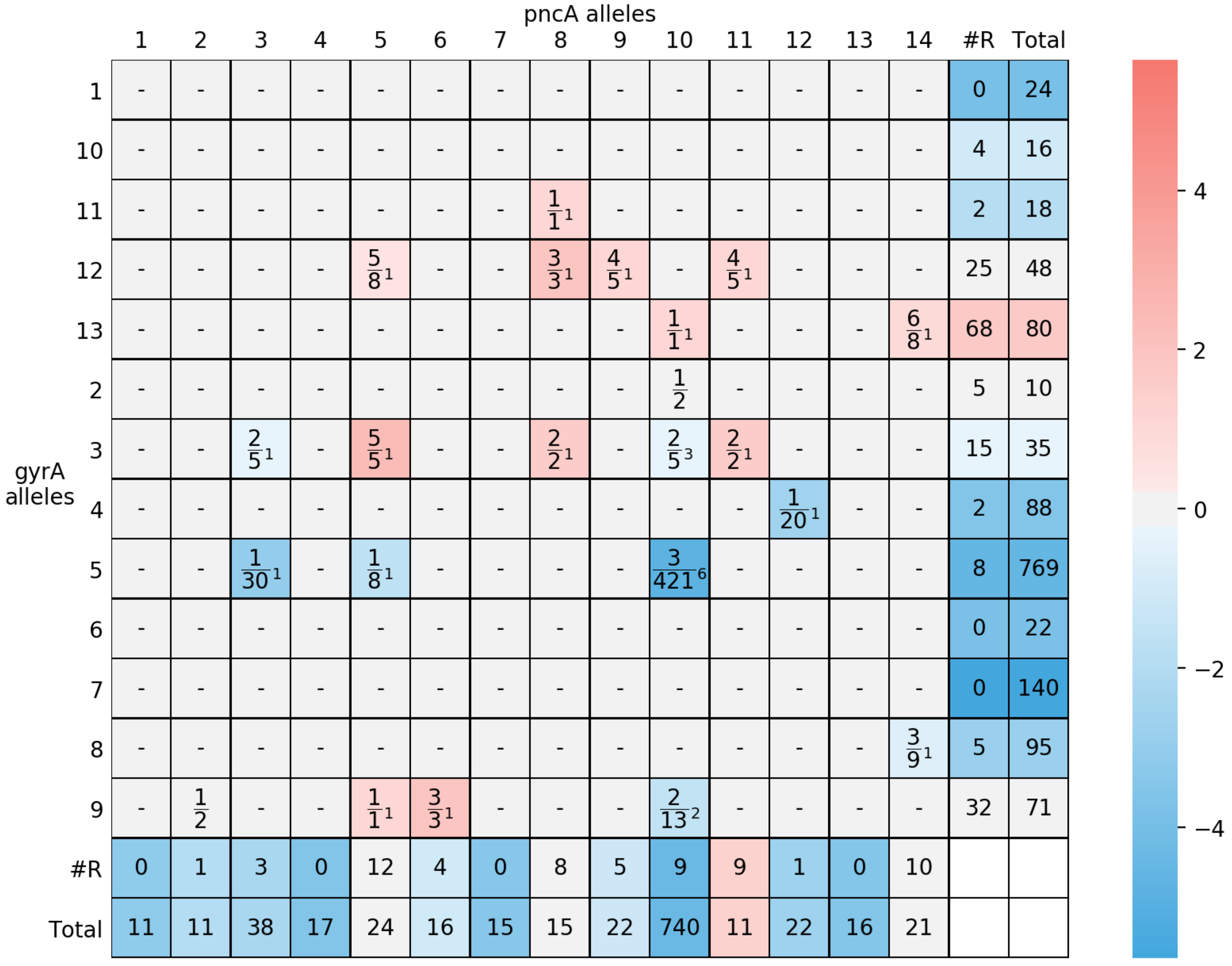

gyrA alleles \ pncA alleles	1	2	3	4	5	6	7	8	9	10	11	12	13	14	#R	Total
1	-	-	-	-	-	-	-	-	-	-	-	-	-	-	0	24
10	-	-	-	-	-	-	-	-	-	-	-	-	-	-	4	16
11	-	-	-	-	-	-	-	$\frac{1}{1}^{1}$	-	-	-	-	-	-	2	18
12	-	-	-	-	$\frac{5}{8}^{1}$	-	-	$\frac{3}{3}^{1}$	$\frac{4}{5}^{1}$	-	$\frac{4}{5}^{1}$	-	-	-	25	48
13	-	-	-	-	-	-	-	-	-	$\frac{1}{1}^{1}$	-	-	-	$\frac{6}{8}^{1}$	68	80
2	-	-	-	-	-	-	-	-	-	$\frac{1}{2}$	-	-	-	-	5	10
3	-	-	$\frac{2}{5}^{1}$	-	$\frac{5}{5}^{1}$	-	-	$\frac{2}{2}^{1}$	-	$\frac{2}{5}^{3}$	$\frac{2}{2}^{1}$	-	-	-	15	35
4	-	-	-	-	-	-	-	-	-	-	-	$\frac{1}{20}^{1}$	-	-	2	88
5	-	-	$\frac{1}{30}^{1}$	-	$\frac{1}{8}^{1}$	-	-	-	-	$\frac{3}{421}^{6}$	-	-	-	-	8	769
6	-	-	-	-	-	-	-	-	-	-	-	-	-	-	0	22
7	-	-	-	-	-	-	-	-	-	-	-	-	-	-	0	140
8	-	-	-	-	-	-	-	-	-	-	-	-	-	$\frac{3}{9}^{1}$	5	95
9	-	$\frac{1}{2}$	-	-	$\frac{1}{1}^{1}$	$\frac{3}{3}^{1}$	-	-	-	$\frac{2}{13}^{2}$	-	-	-	-	32	71
#R	0	1	3	0	12	4	0	8	5	9	9	1	0	10		
Total	11	11	38	17	24	16	15	15	22	740	11	22	16	21		

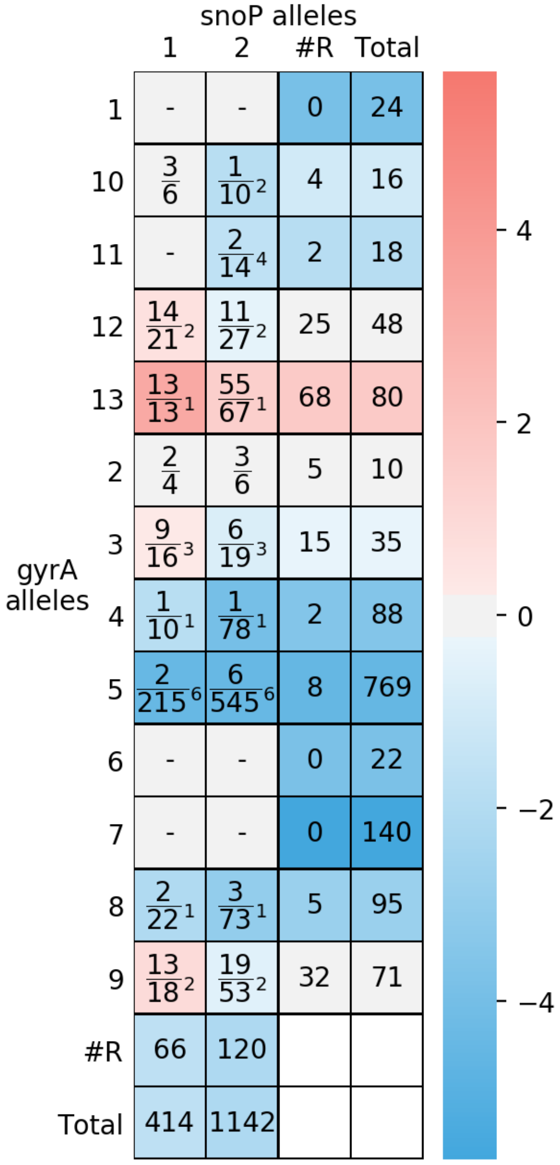
snoP alleles
1
2
#R
Total
gyrA alleles
1 - - 0 24
10 3/6 1/10 2 4 16
11 - 2/14 4 2 18
12 14/21 2 11/27 2 25 48
13 13/13 1 55/67 1 68 80
2 2/4 3/6 5 10
3 9/16 3 6/19 3 15 35
4 1/10 1 1/78 1 2 88
5 2/215 6 6/545 6 8 769
6 - - 0 22
7 - - 0 140
8 2/22 1 3/73 1 5 95
9 13/18 2 19/53 2 32 71
#R 66 120
Total 414 1142
4
2
0
−2
−4

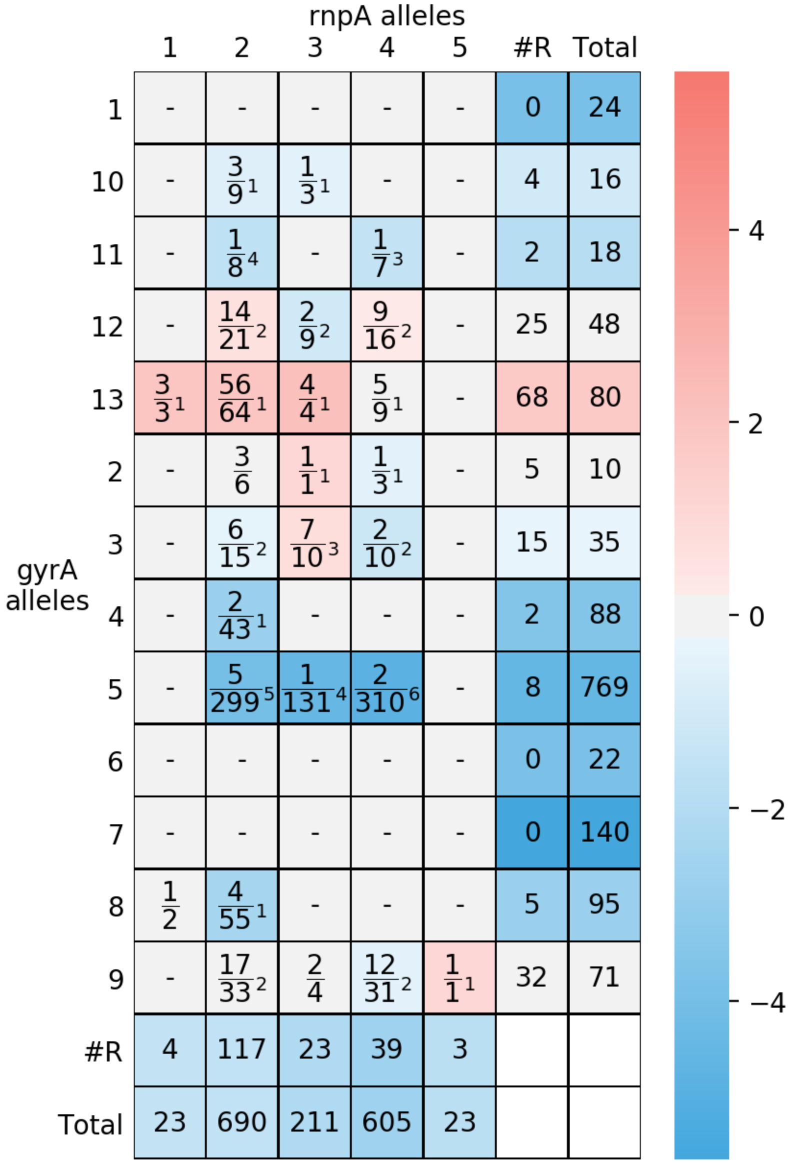
rnpA alleles
1 2 3 4 5 #R Total
gyrA alleles
1 - - - - - 0 24
10 - 3/9 1 1/3 1 - - 4 16
11 - 1/8 4 - 1/7 3 - 2 18
12 - 14/21 2 2/9 2 9/16 2 - 25 48
13 3/3 1 56/64 1 4/4 1 5/9 1 - 68 80
2 - 3/6 1/1 1 1/3 1 - 5 10
3 - 6/15 2 7/10 3 2/10 2 - 15 35
4 - 2/43 1 - - - 2 88
5 - 5/299 5 1/131 4 2/310 6 - 8 769
6 - - - - - 0 22
7 - - - - - 0 140
8 1/2 4/55 1 - - - 5 95
9 - 17/33 2 2/4 12/31 2 1/1 1 32 71
#R 4 117 23 39 3
Total 23 690 211 605 23
4
2
0
−2
−4

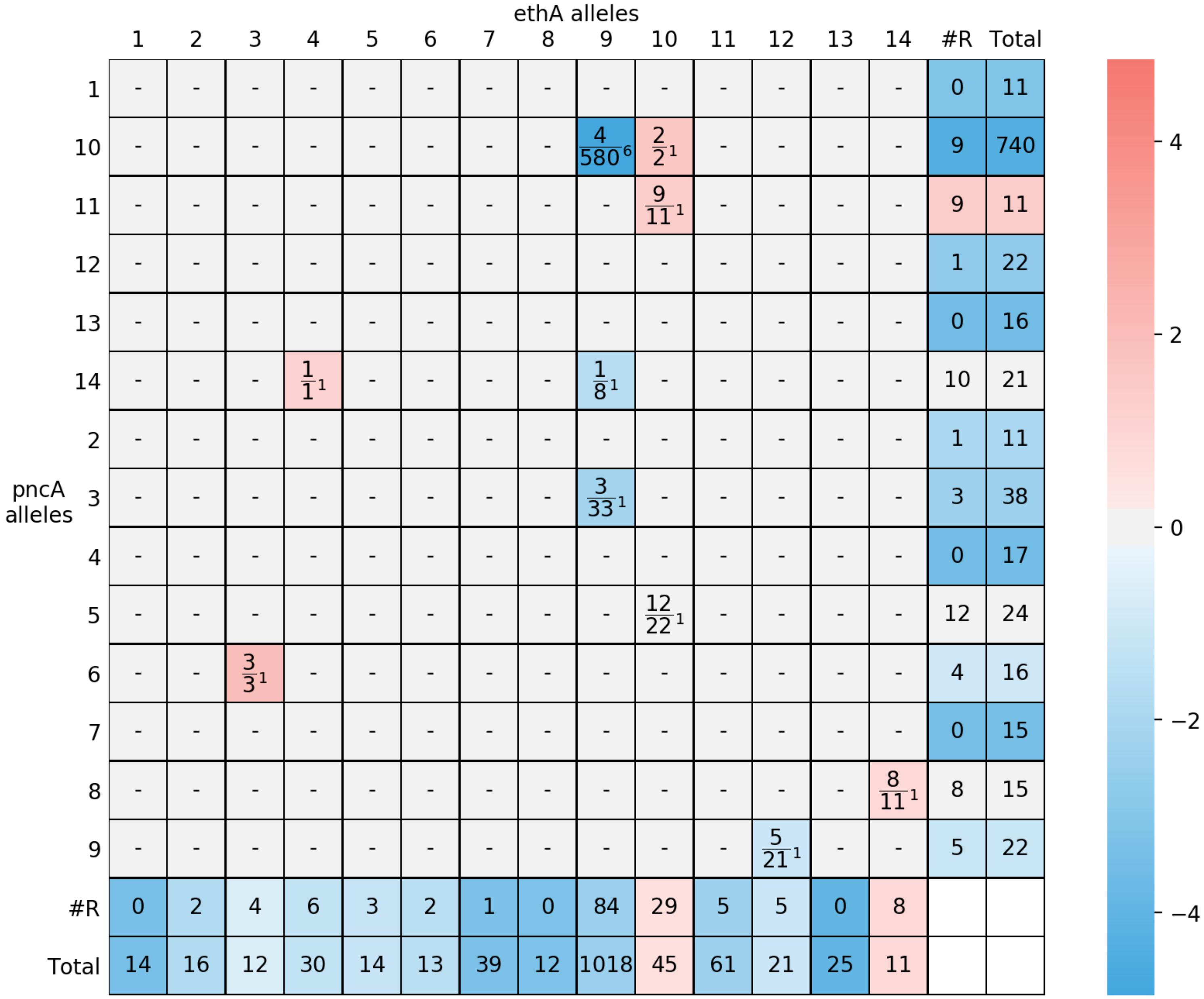
ethA alleles
1 2 3 4 5 6 7 8 9 10 11 12 13 14 #R Total
pncA alleles
1: - - - - - - - - - - - - - - 0 11
10: - - - - - - - - 4/580 6 2/2 1 - - - - 9 740
11: - - - - - - - - - 9/11 1 - - - - 9 11
12: - - - - - - - - - - - - - - 1 22
13: - - - - - - - - - - - - - - 0 16
14: - - - 1/1 1 - - - - 1/8 1 - - - - - 10 21
2: - - - - - - - - - - - - - - 1 11
3: - - - - - - - - 3/33 1 - - - - - 3 38
4: - - - - - - - - - - - - - - 0 17
5: - - - - - - - - - 12/22 1 - - - - 12 24
6: - - 3/3 1 - - - - - - - - - - - 4 16
7: - - - - - - - - - - - - - - 0 15
8: - - - - - - - - - - - - - 8/11 1 8 15
9: - - - - - - - - - - - 5/21 1 - - 5 22
#R: 0 2 4 6 3 2 1 0 84 29 5 5 0 8
Total: 14 16 12 30 14 13 39 12 1018 45 61 21 25 11
4
2
0
−2
−4

thyA alleles

gyrA alleles	1	2	3	4	5	6	#R	Total
1	-	-	-	-	-	-	0	24
10	-	-	-	$\frac{1}{13}^{2}$	-	-	4	16
11	$\frac{1}{1}^{1}$	-	-	-	-	-	2	18
12	$\frac{3}{3}^{1}$	-	-	$\frac{20}{41}^{2}$	-	-	25	48
13	-	$\frac{66}{74}^{1}$	-	-	$\frac{2}{2}^{1}$	-	68	80
2	-	-	-	$\frac{3}{8}^{1}$	-	-	5	10
3	$\frac{2}{2}^{1}$	-	-	$\frac{13}{31}^{3}$	-	-	15	35
4	-	-	-	$\frac{2}{78}^{1}$	-	-	2	88
5	-	-	-	$\frac{5}{626}^{6}$	-	$\frac{3}{40}^{3}$	8	769
6	-	-	-	-	-	-	0	22
7	-	-	-	-	-	-	0	140
8	-	$\frac{4}{90}^{1}$	-	-	$\frac{1}{5}^{1}$	-	5	95
9	-	-	$\frac{3}{3}^{1}$	$\frac{25}{60}^{2}$	-	$\frac{1}{1}^{1}$	32	71
#R	8	73	3	79	3	7		
Total	12	244	15	1151	12	73		

Colour scale: 4, 2, 0, −2, −4

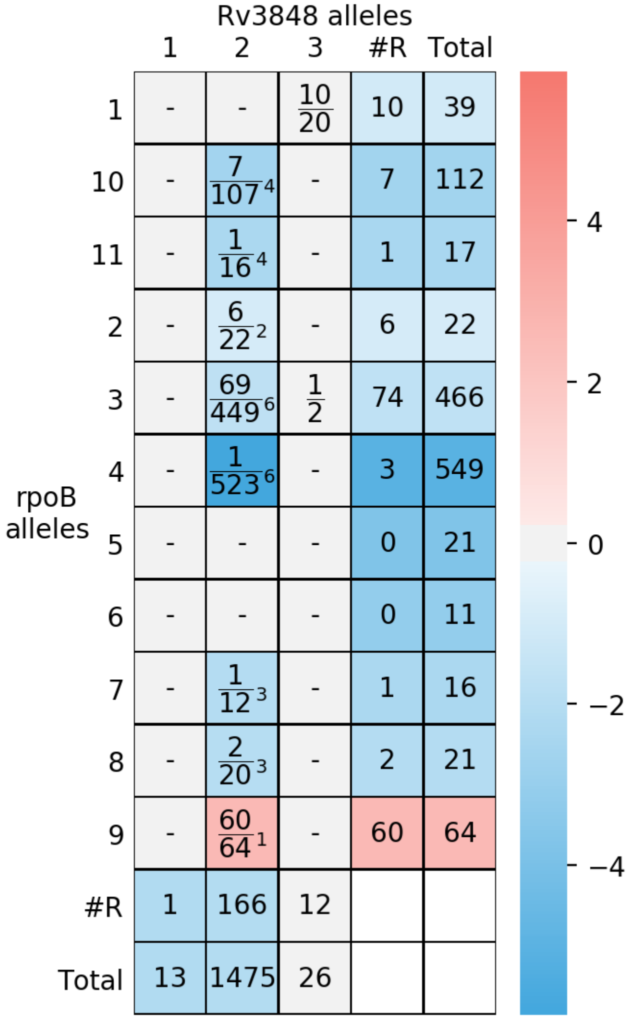
Rv3848 alleles
1
2
3
#R
Total
rpoB alleles
1 - - 10/20 10 39
10 - 7/107 4 - 7 112
11 - 1/16 4 - 1 17
2 - 6/22 2 - 6 22
3 - 69/449 6 1/2 74 466
4 - 1/523 6 - 3 549
5 - - - 0 21
6 - - - 0 11
7 - 1/12 3 - 1 16
8 - 2/20 3 - 2 21
9 - 60/64 1 - 60 64
#R 1 166 12
Total 13 1475 26
4
2
0
−2
−4

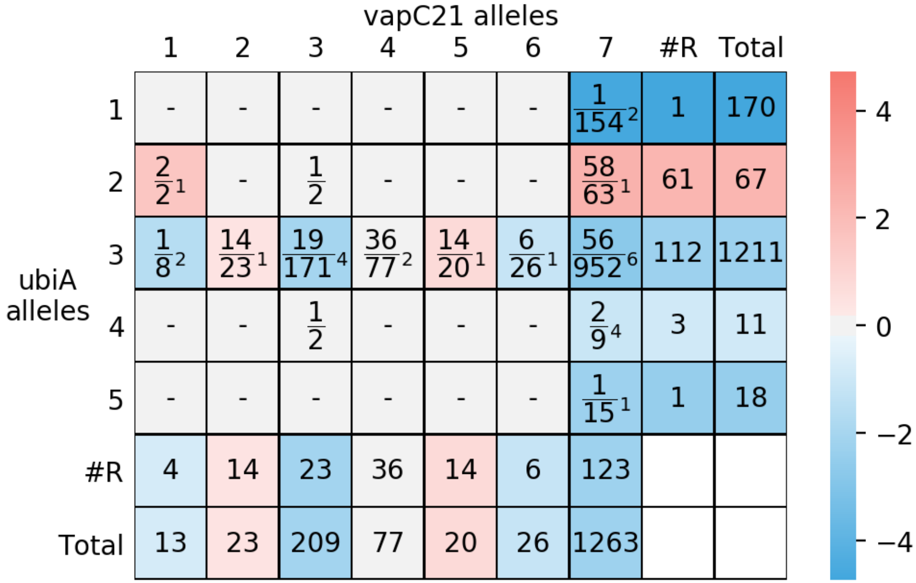
vapC21 alleles
1
2
3
4
5
6
7
#R
Total
ubiA alleles
1
-
-
-
-
-
-
1/154 2
1
170
2
2/2 1
-
1/2
-
-
-
58/63 1
61
67
3
1/8 2
14/23 1
19/171 4
36/77 2
14/20 1
6/26 1
56/952 6
112
1211
4
-
-
1/2
-
-
-
2/9 4
3
11
5
-
-
-
-
-
-
1/15 1
1
18
#R
4
14
23
36
14
6
123
Total
13
23
209
77
20
26
1263
4
2
0
−2
−4

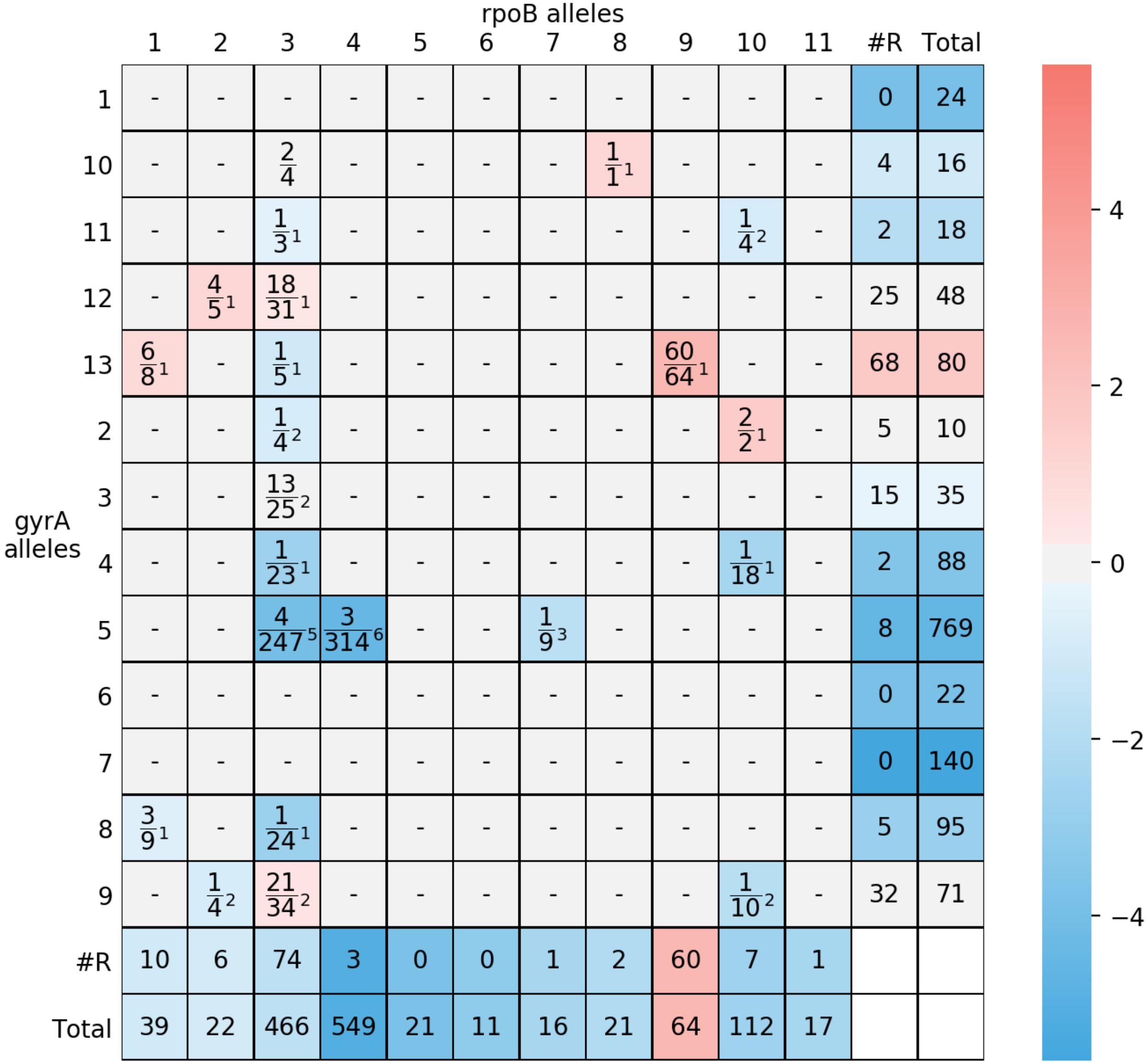
rpoB alleles
1 2 3 4 5 6 7 8 9 10 11 #R Total
gyrA alleles
1 - - - - - - - - - - - 0 24
10 - - 2/4 - - - - 1/1 1 - - - 4 16
11 - - 1/3 1 - - - - - - 1/4 2 - 2 18
12 - 4/5 1 18/31 1 - - - - - - - - 25 48
13 6/8 1 - 1/5 1 - - - - - 60/64 1 - - 68 80
2 - - 1/4 2 - - - - - - 2/2 1 - 5 10
3 - - 13/25 2 - - - - - - - - 15 35
4 - - 1/23 1 - - - - - - 1/18 1 - 2 88
5 - - 4/247 5 3/314 6 - - 1/9 3 - - - - 8 769
6 - - - - - - - - - - - 0 22
7 - - - - - - - - - - - 0 140
8 3/9 1 - 1/24 1 - - - - - - - - 5 95
9 - 1/4 2 21/34 2 - - - - - - 1/10 2 - 32 71
#R 10 6 74 3 0 0 1 2 60 7 1
Total 39 22 466 549 21 11 16 21 64 112 17
4
2
0
−2
−4

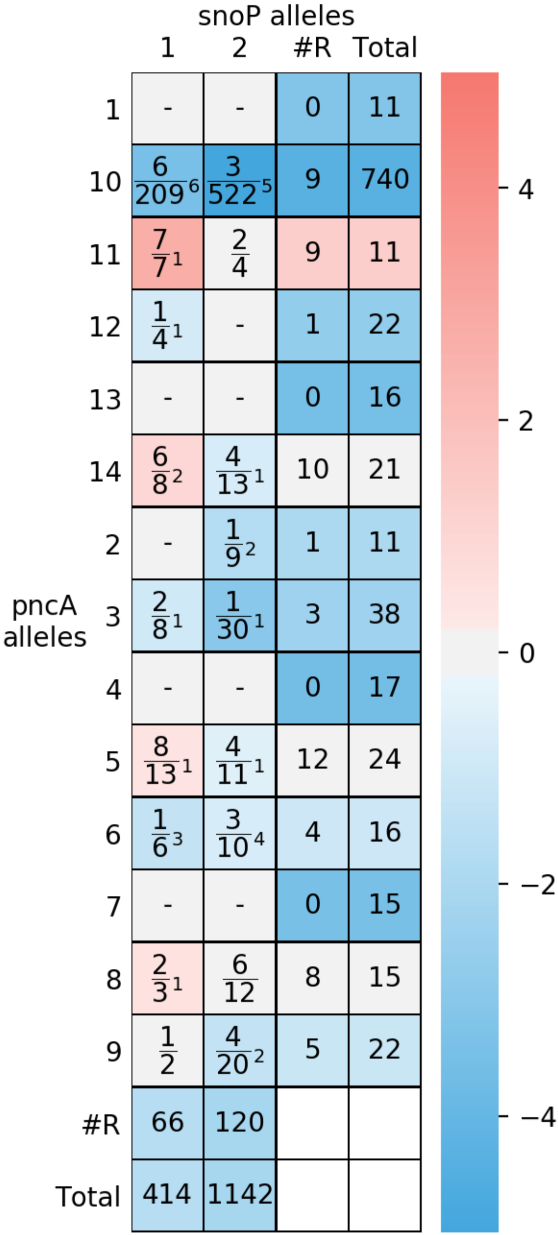
snoP alleles
1
2
#R
Total
pncA alleles
1 - - 0 11
10 6/209 6 3/522 5 9 740
11 7/7 1 2/4 9 11
12 1/4 1 - 1 22
13 - - 0 16
14 6/8 2 4/13 1 10 21
2 - 1/9 2 1 11
3 2/8 1 1/30 1 3 38
4 - - 0 17
5 8/13 1 4/11 1 12 24
6 1/6 3 3/10 4 4 16
7 - - 0 15
8 2/3 1 6/12 8 15
9 1/2 4/20 2 5 22
#R 66 120
Total 414 1142
4
2
0
−2
−4

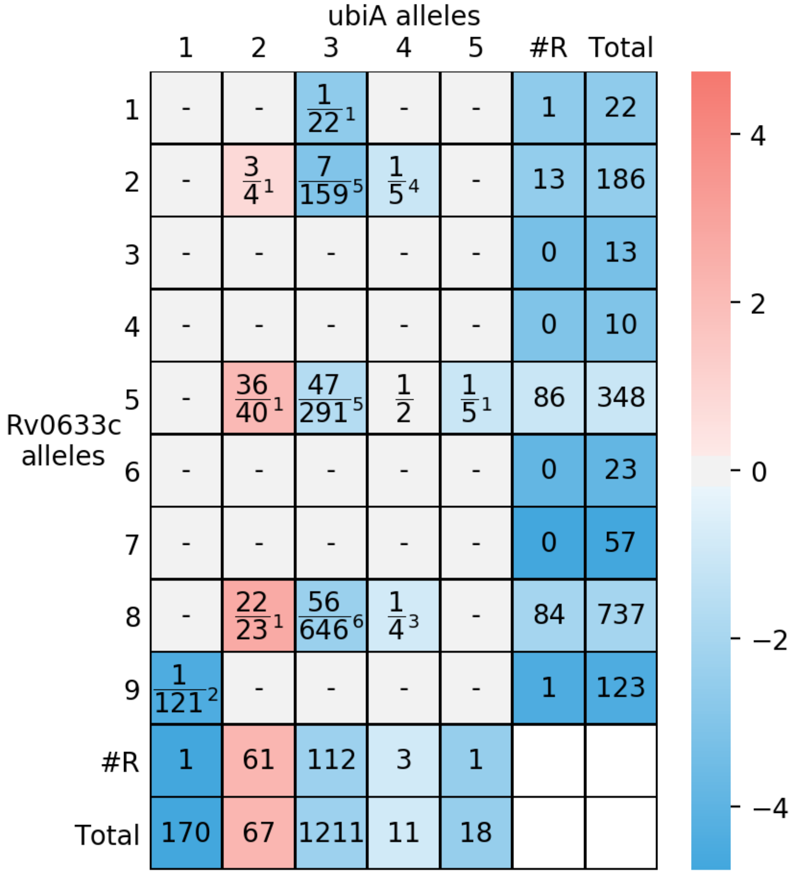
ubiA alleles
1 2 3 4 5 #R Total
Rv0633c alleles
1 - - 1/22 1 - - 1 22
2 - 3/4 1 7/159 5 1/5 4 - 13 186
3 - - - - - 0 13
4 - - - - - 0 10
5 - 36/40 1 47/291 5 1/2 1/5 1 86 348
6 - - - - - 0 23
7 - - - - - 0 57
8 - 22/23 1 56/646 6 1/4 3 - 84 737
9 1/121 2 - - - - 1 123
#R 1 61 112 3 1
Total 170 67 1211 11 18
4
2
0
−2
−4

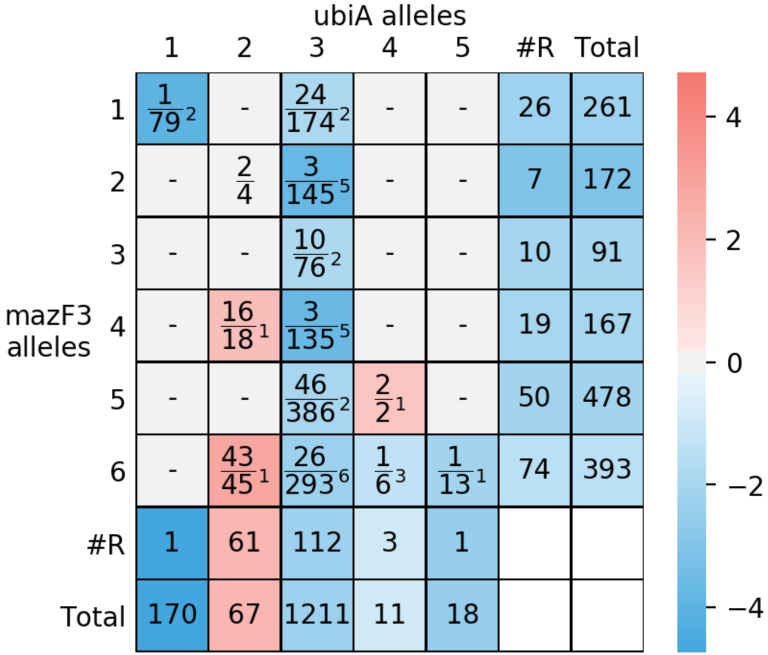

ubiA alleles
1
2
3
4
5
#R
Total
mazF3 alleles
1
2
3
4
5
6
#R
Total
1/79 2
-
24/174 2
-
-
26
261
-
2/4
3/145 5
-
-
7
172
-
-
10/76 2
-
-
10
91
-
16/18 1
3/135 5
-
-
19
167
-
-
46/386 2
2/2 1
-
50
478
-
43/45 1
26/293 6
1/6 3
1/13 1
74
393
1
61
112
3
1
170
67
1211
11
18
4
2
0
−2
−4

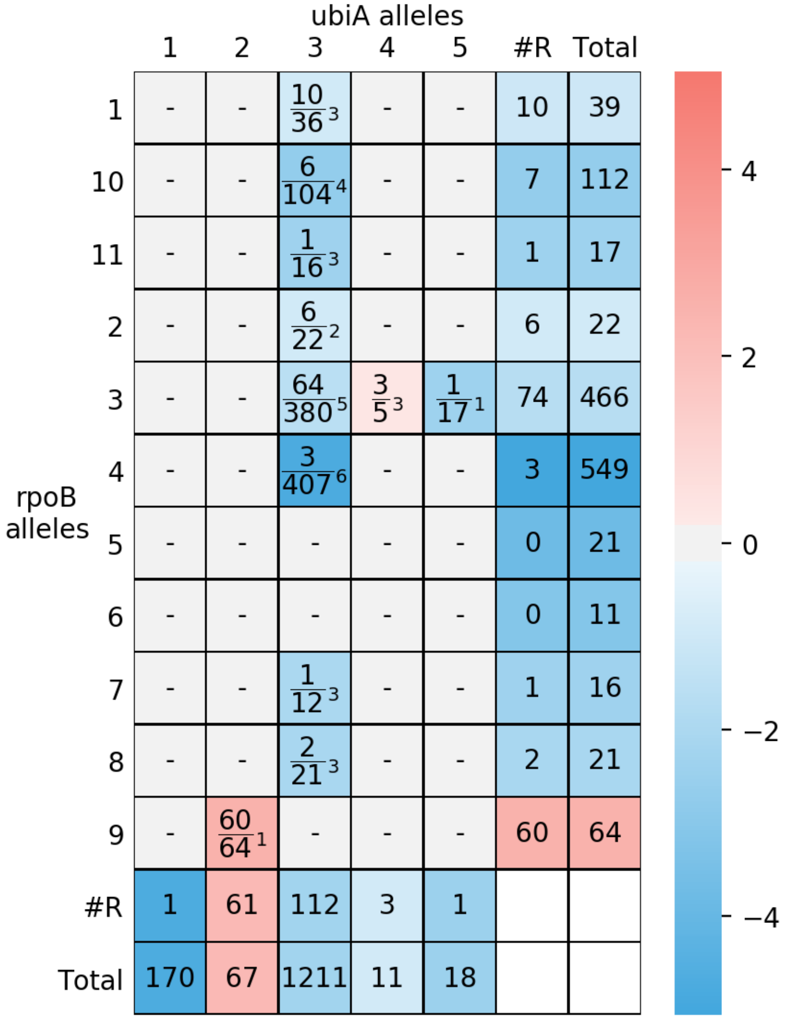

ubiA alleles
1 2 3 4 5 #R Total
rpoB alleles
1: - - 10/36 3 - - 10 39
10: - - 6/104 4 - - 7 112
11: - - 1/16 3 - - 1 17
2: - - 6/22 2 - - 6 22
3: - - 64/380 5 3/5 3 1/17 1 74 466
4: - - 3/407 6 - - 3 549
5: - - - - - 0 21
6: - - - - - 0 11
7: - - 1/12 3 - - 1 16
8: - - 2/21 3 - - 2 21
9: - 60/64 1 - - - 60 64
#R: 1 61 112 3 1
Total: 170 67 1211 11 18
4
2
0
−2
−4

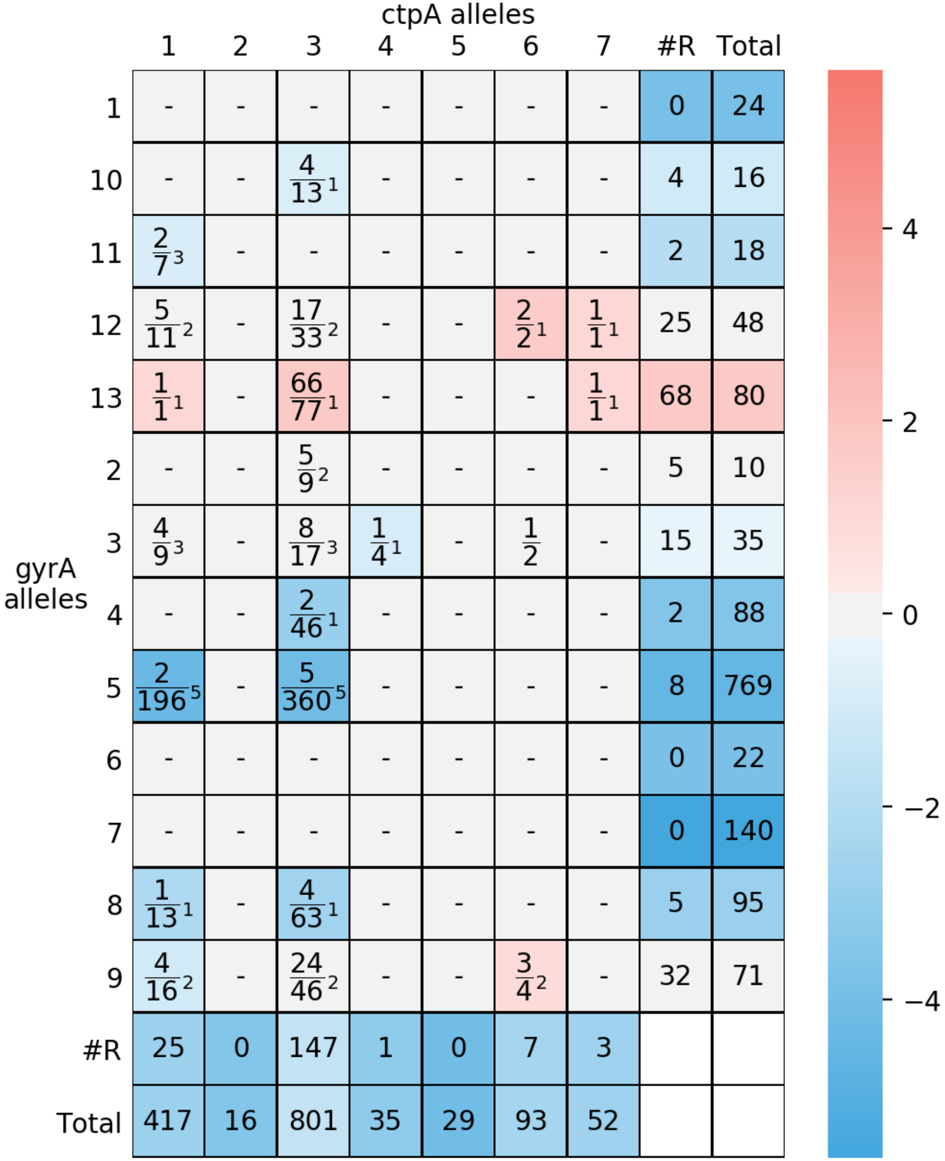
ctpA alleles
1 2 3 4 5 6 7 #R Total
gyrA alleles
1 - - - - - - - 0 24
10 - - 4/13 1 - - - - 4 16
11 2/7 3 - - - - - - 2 18
12 5/11 2 - 17/33 2 - - 2/2 1 1/1 1 25 48
13 1/1 1 - 66/77 1 - - - 1/1 1 68 80
2 - - 5/9 2 - - - - 5 10
3 4/9 3 - 8/17 3 1/4 1 - 1/2 - 15 35
4 - - 2/46 1 - - - - 2 88
5 2/196 5 - 5/360 5 - - - - 8 769
6 - - - - - - - 0 22
7 - - - - - - - 0 140
8 1/13 1 - 4/63 1 - - - - 5 95
9 4/16 2 - 24/46 2 - - 3/4 2 - 32 71
#R 25 0 147 1 0 7 3
Total 417 16 801 35 29 93 52
4
2
0
−2
−4
