# Supplementary material for: Machine learning and structural analysis of Mycobacterium tuberculosis pan-genome identifies genetic signatures of antibiotic resistance
Source: Nat Commun. 2018 Oct 17;9:4306. doi: 10.1038/s41467-018-06634-y (PMC6193043; doi:10.1038/s41467-018-06634-y)
Supplement: Supplementary file 8 — Supplementary Data 5 [file 41467_2018_6634_MOESM8_ESM.zip › Supplementary Data 5/isoniazid_epistasis.pdf]

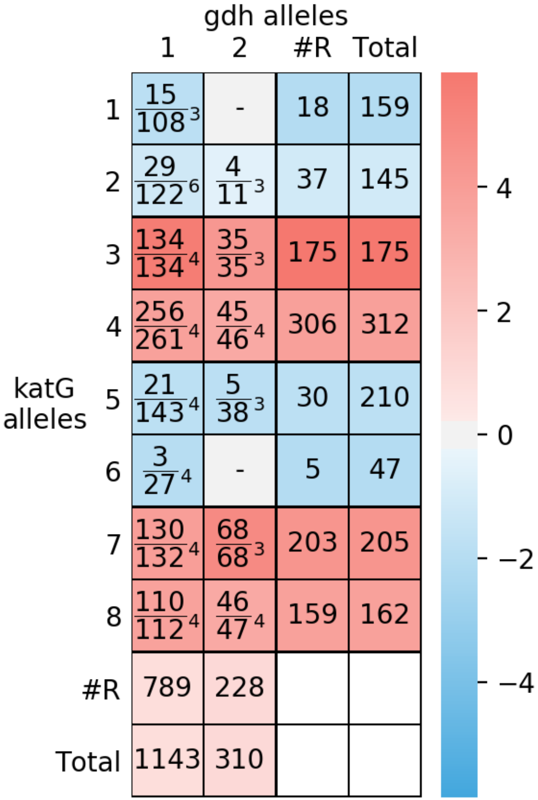
gdh alleles
1
2
#R
Total
katG alleles
1
15/108 3
-
18
159
2
29/122 6
4/11 3
37
145
3
134/134 4
35/35 3
175
175
4
256/261 4
45/46 4
306
312
5
21/143 4
5/38 3
30
210
6
3/27 4
-
5
47
7
130/132 4
68/68 3
203
205
8
110/112 4
46/47 4
159
162
#R
789
228
Total
1143
310
4
2
0
−2
−4

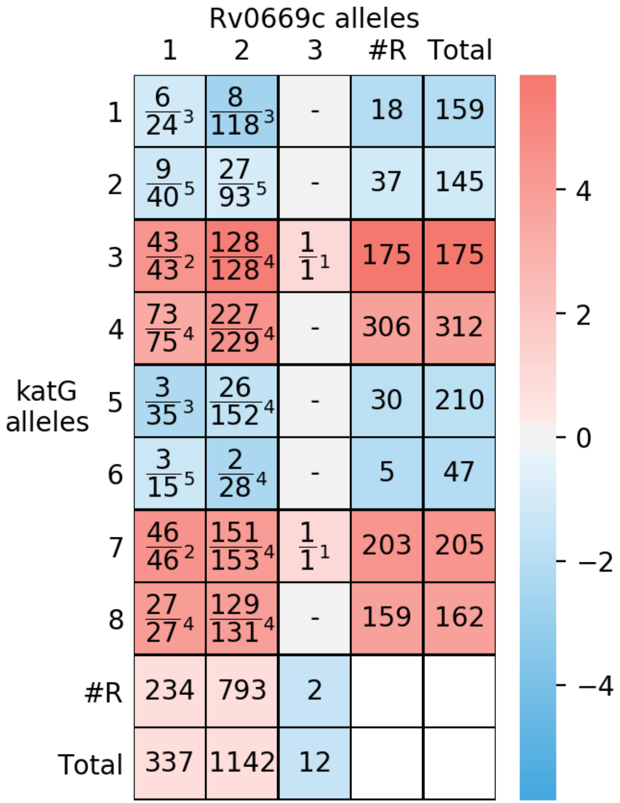
Rv0669c alleles
1
2
3
#R
Total
katG alleles
1
6/24 3
8/118 3
-
18
159
2
9/40 5
27/93 5
-
37
145
3
43/43 2
128/128 4
1/1 1
175
175
4
73/75 4
227/229 4
-
306
312
5
3/35 3
26/152 4
-
30
210
6
3/15 5
2/28 4
-
5
47
7
46/46 2
151/153 4
1/1 1
203
205
8
27/27 4
129/131 4
-
159
162
#R
234
793
2
Total
337
1142
12
4
2
0
−2
−4

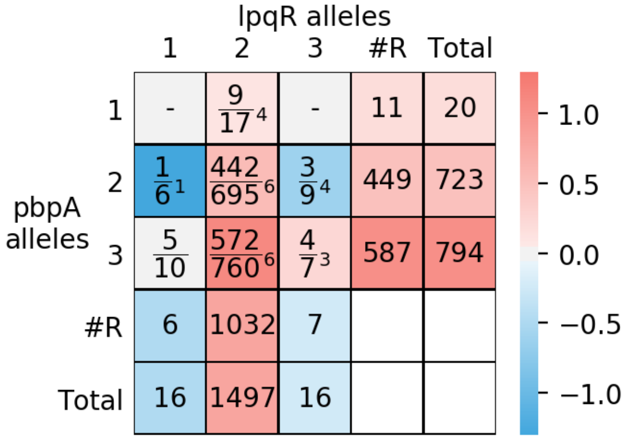
lpqR alleles
1
2
3
#R
Total
pbpA alleles
1
2
3
#R
Total
-
9/17 4
-
11
20
1/6 1
442/695 6
3/9 4
449
723
5/10
572/760 6
4/7 3
587
794
6
1032
7
16
1497
16
1.0
0.5
0.0
−0.5
−1.0

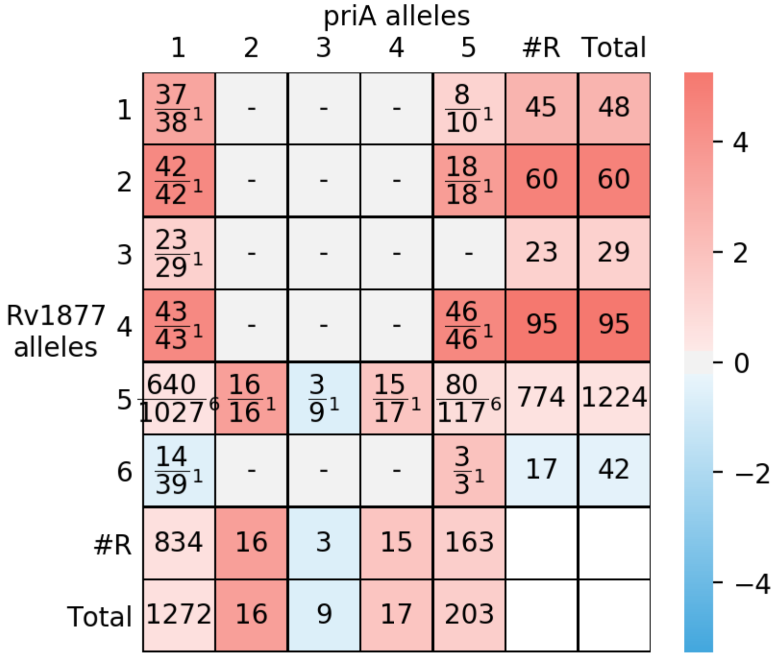
priA alleles
1
2
3
4
5
#R
Total
Rv1877 alleles
1
2
3
4
5
6
#R
Total
37/38 1
8/10 1
45
48
42/42 1
18/18 1
60
60
23/29 1
23
29
43/43 1
46/46 1
95
95
640/1027 6
16/16 1
3/9 1
15/17 1
80/117 6
774
1224
14/39 1
3/3 1
17
42
834
16
3
15
163
1272
16
9
17
203
4
2
0
−2
−4
-

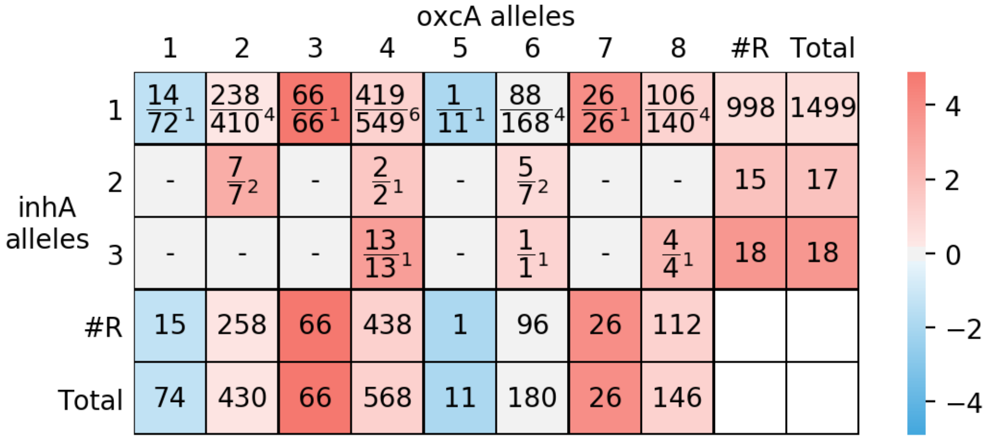
oxcA alleles
1
2
3
4
5
6
7
8
#R
Total
inhA alleles
1
2
3
#R
Total
14/72 1
238/410 4
66/66 1
419/549 6
1/11 1
88/168 4
26/26 1
106/140 4
998
1499
-
7/7 2
-
2/2 1
-
5/7 2
-
-
15
17
-
-
-
13/13 1
-
1/1 1
-
4/4 1
18
18
15
258
66
438
1
96
26
112
74
430
66
568
11
180
26
146
4
2
0
−2
−4

Rv0265c alleles

katG alleles	1	2	#R	Total
1	$\frac{10}{82}$ 3	$\frac{8}{71}$ 3	18	159
2	$\frac{24}{83}$ 5	$\frac{13}{60}$ 6	37	145
3	$\frac{67}{67}$ 4	$\frac{103}{103}$ 3	175	175
4	$\frac{183}{185}$ 4	$\frac{120}{124}$ 4	306	312
5	$\frac{16}{89}$ 3	$\frac{14}{115}$ 4	30	210
6	$\frac{4}{32}$ 5	$\frac{1}{12}$ 3	5	47
7	$\frac{79}{80}$ 3	$\frac{121}{122}$ 4	203	205
8	$\frac{99}{101}$ 4	$\frac{60}{61}$ 4	159	162
#R	550	492		
Total	796	732		

Color scale: 4, 2, 0, −2, −4

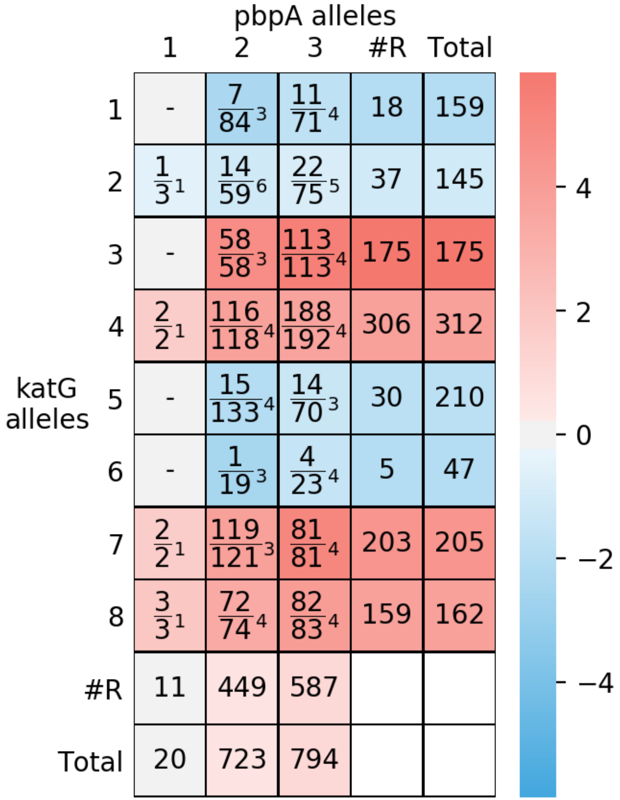
pbpA alleles
1 2 3 #R Total
katG alleles
1 - 7/84 3 11/71 4 18 159
2 1/3 1 14/59 6 22/75 5 37 145
3 - 58/58 3 113/113 4 175 175
4 2/2 1 116/118 4 188/192 4 306 312
5 - 15/133 4 14/70 3 30 210
6 - 1/19 3 4/23 4 5 47
7 2/2 1 119/121 3 81/81 4 203 205
8 3/3 1 72/74 4 82/83 4 159 162
#R 11 449 587
Total 20 723 794
4
2
0
−2
−4

katG alleles \ oxcA alleles	1	2	3	4	5	6	7	8	#R	Total
1	$\frac{1}{30}^{1}$	$\frac{12}{68}^{4}$	-	-	-	$\frac{3}{48}^{3}$	-	-	18	159
2	-	-	-	$\frac{25}{106}^{6}$	-	-	-	$\frac{12}{37}^{4}$	37	145
3	$\frac{4}{4}^{1}$	$\frac{78}{78}^{3}$	$\frac{29}{29}^{1}$	$\frac{1}{1}^{1}$	$\frac{1}{1}^{1}$	$\frac{43}{43}^{3}$	$\frac{14}{14}^{1}$	$\frac{1}{1}^{1}$	175	175
4	-	-	-	$\frac{241}{245}^{4}$	-	-	-	$\frac{62}{64}^{4}$	306	312
5	$\frac{3}{32}^{1}$	$\frac{17}{118}^{3}$	-	-	-	$\frac{6}{41}^{3}$	-	-	30	210
6	-	-	-	$\frac{5}{40}^{5}$	-	-	-	-	5	47
7	$\frac{5}{5}^{1}$	$\frac{108}{110}^{3}$	$\frac{36}{36}^{1}$	-	-	$\frac{29}{29}^{4}$	$\frac{11}{11}^{1}$	-	203	205
8	-	-	-	$\frac{126}{129}^{4}$	-	-	-	$\frac{28}{28}^{4}$	159	162
#R	15	258	66	438	1	96	26	112		
Total	74	430	66	568	11	180	26	146		

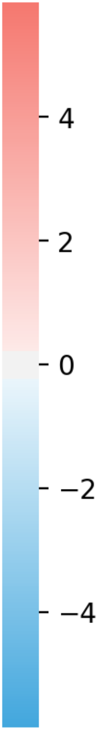

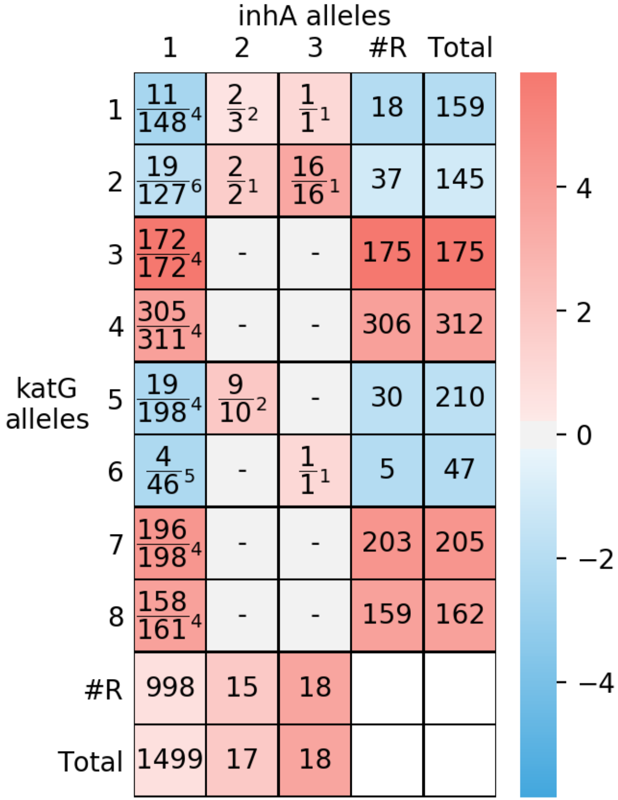

inhA alleles
1
2
3
#R
Total
katG alleles
1
2
3
4
5
6
7
8
#R
Total
11/148 4
2/3 2
1/1 1
18
159
19/127 6
2/2 1
16/16 1
37
145
172/172 4
-
-
175
175
305/311 4
-
-
306
312
19/198 4
9/10 2
-
30
210
4/46 5
-
1/1 1
5
47
196/198 4
-
-
203
205
158/161 4
-
-
159
162
998
15
18
1499
17
18
4
2
0
−2
−4

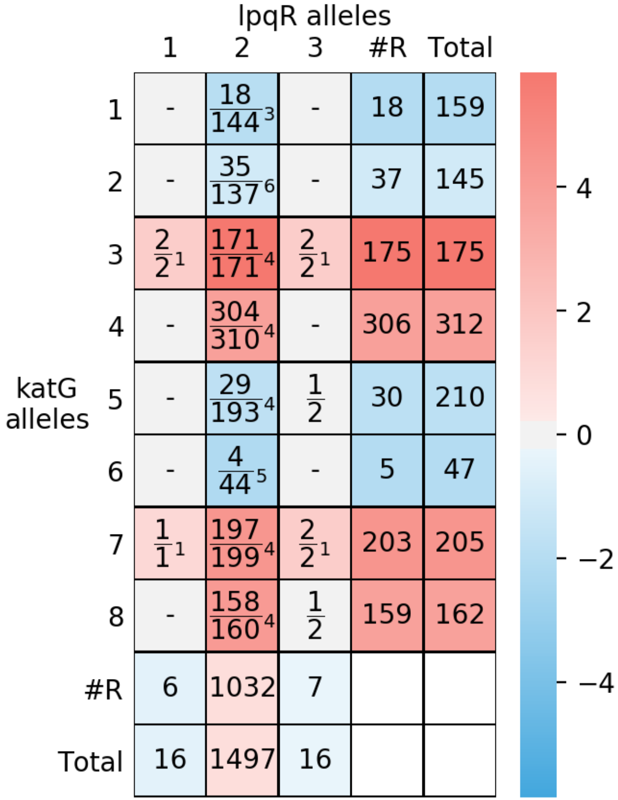
lpqR alleles
1
2
3
#R
Total
katG alleles
1
-
18/144 3
-
18
159
2
-
35/137 6
-
37
145
3
2/2 1
171/171 4
2/2 1
175
175
4
-
304/310 4
-
306
312
5
-
29/193 4
1/2
30
210
6
-
4/44 5
-
5
47
7
1/1 1
197/199 4
2/2 1
203
205
8
-
158/160 4
1/2
159
162
#R
6
1032
7
Total
16
1497
16
4
2
0
−2
−4

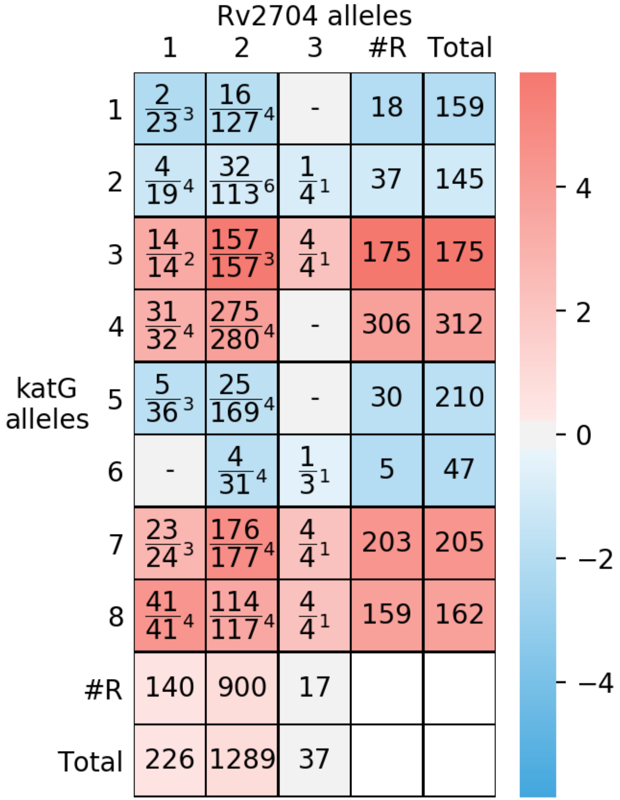
Rv2704 alleles
1
2
3
#R
Total
katG alleles
1
2/23 3
16/127 4
-
18
159
2
4/19 4
32/113 6
1/4 1
37
145
3
14/14 2
157/157 3
4/4 1
175
175
4
31/32 4
275/280 4
-
306
312
5
5/36 3
25/169 4
-
30
210
6
-
4/31 4
1/3 1
5
47
7
23/24 3
176/177 4
4/4 1
203
205
8
41/41 4
114/117 4
4/4 1
159
162
#R
140
900
17
Total
226
1289
37
4
2
0
−2
−4

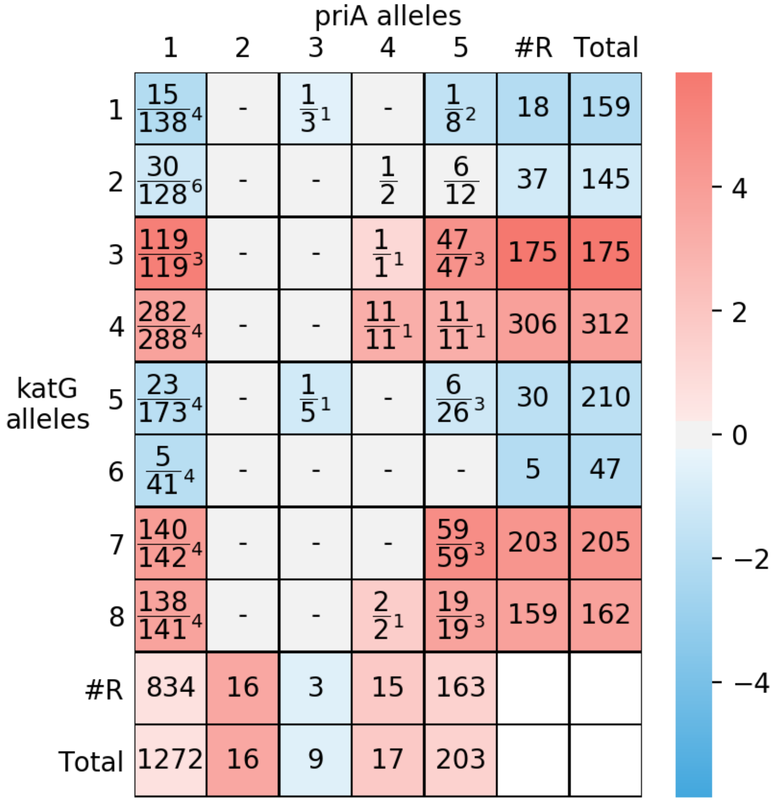

priA alleles
1
2
3
4
5
#R
Total
katG alleles
1: 15/138 [4], -, 1/3 [1], -, 1/8 [2], 18, 159
2: 30/128 [6], -, -, 1/2, 6/12, 37, 145
3: 119/119 [3], -, -, 1/1 [1], 47/47 [3], 175, 175
4: 282/288 [4], -, -, 11/11 [1], 11/11 [1], 306, 312
5: 23/173 [4], -, 1/5 [1], -, 6/26 [3], 30, 210
6: 5/41 [4], -, -, -, -, 5, 47
7: 140/142 [4], -, -, -, 59/59 [3], 203, 205
8: 138/141 [4], -, -, 2/2 [1], 19/19 [3], 159, 162
#R: 834, 16, 3, 15, 163
Total: 1272, 16, 9, 17, 203
4
2
0
−2
−4
