# Supplementary material for: Machine learning and structural analysis of Mycobacterium tuberculosis pan-genome identifies genetic signatures of antibiotic resistance
Source: Nat Commun. 2018 Oct 17;9:4306. doi: 10.1038/s41467-018-06634-y (PMC6193043; doi:10.1038/s41467-018-06634-y)

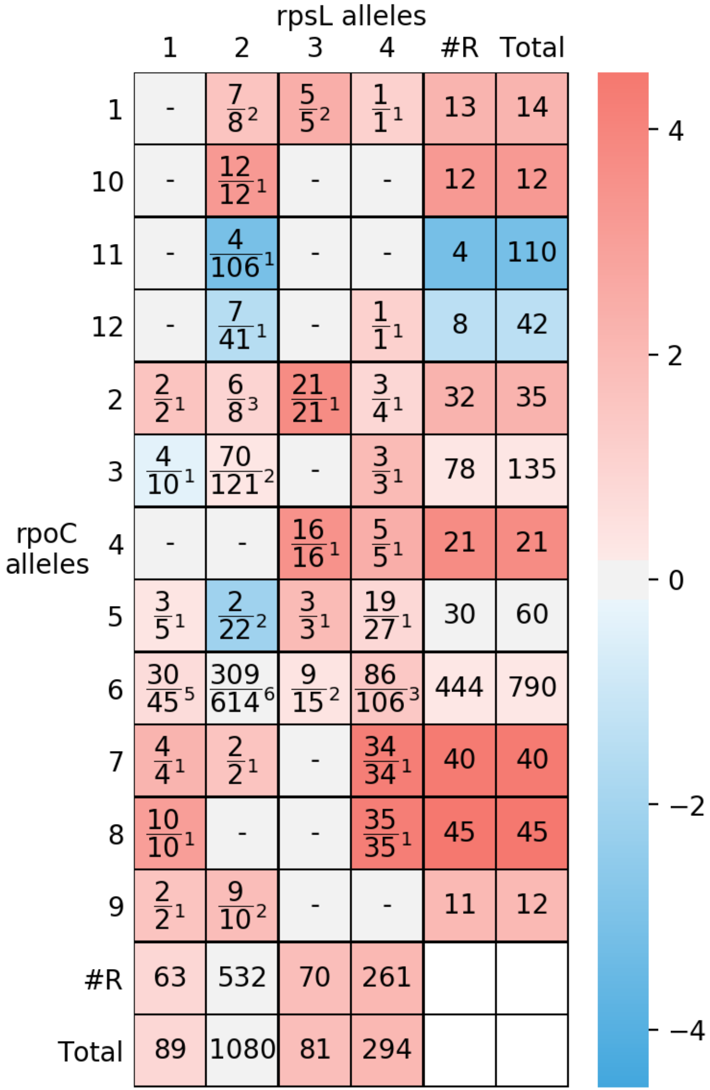

rpsL alleles
1
2
3
4
#R
Total
rpoC alleles
1
-
7/8 2
5/5 2
1/1 1
13
14
10
-
12/12 1
-
-
12
12
11
-
4/106 1
-
-
4
110
12
-
7/41 1
-
1/1 1
8
42
2
2/2 1
6/8 3
21/21 1
3/4 1
32
35
3
4/10 1
70/121 2
-
3/3 1
78
135
4
-
-
16/16 1
5/5 1
21
21
5
3/5 1
2/22 2
3/3 1
19/27 1
30
60
6
30/45 5
309/614 6
9/15 2
86/106 3
444
790
7
4/4 1
2/2 1
-
34/34 1
40
40
8
10/10 1
-
-
35/35 1
45
45
9
2/2 1
9/10 2
-
-
11
12
#R
63
532
70
261
Total
89
1080
81
294
4
2
0
−2
−4

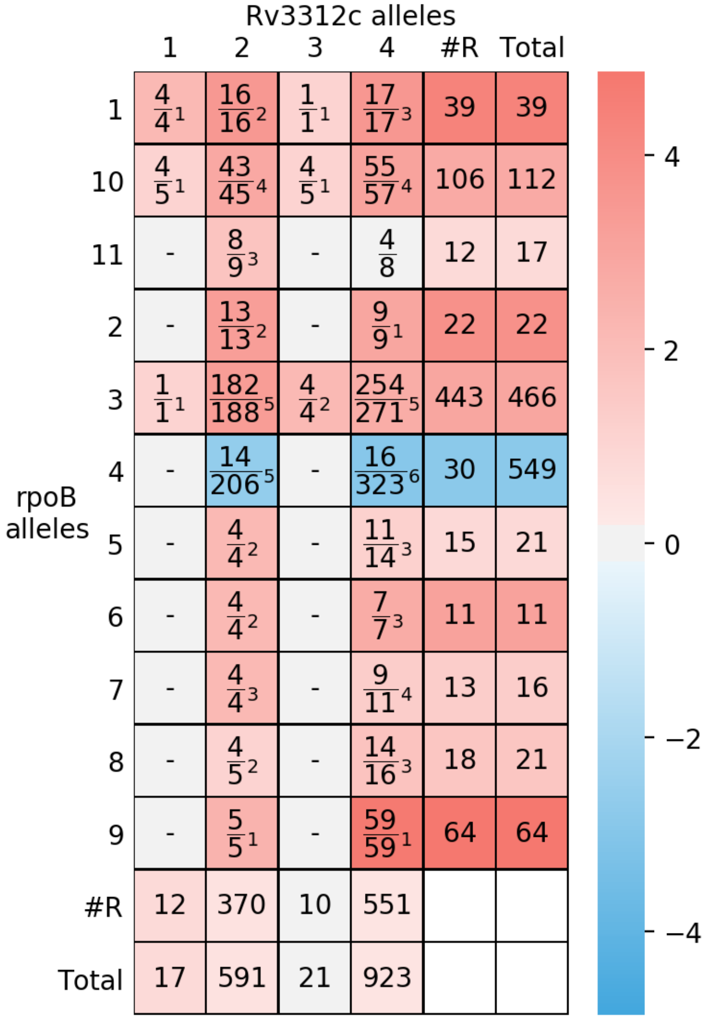
Rv3312c alleles
1
2
3
4
#R
Total
rpoB alleles
1: 4/4 1, 16/16 2, 1/1 1, 17/17 3, 39, 39
10: 4/5 1, 43/45 4, 4/5 1, 55/57 4, 106, 112
11: -, 8/9 3, -, 4/8, 12, 17
2: -, 13/13 2, -, 9/9 1, 22, 22
3: 1/1 1, 182/188 5, 4/4 2, 254/271 5, 443, 466
4: -, 14/206 5, -, 16/323 6, 30, 549
5: -, 4/4 2, -, 11/14 3, 15, 21
6: -, 4/4 2, -, 7/7 3, 11, 11
7: -, 4/4 3, -, 9/11 4, 13, 16
8: -, 4/5 2, -, 14/16 3, 18, 21
9: -, 5/5 1, -, 59/59 1, 64, 64
#R: 12, 370, 10, 551
Total: 17, 591, 21, 923
4
2
0
−2
−4

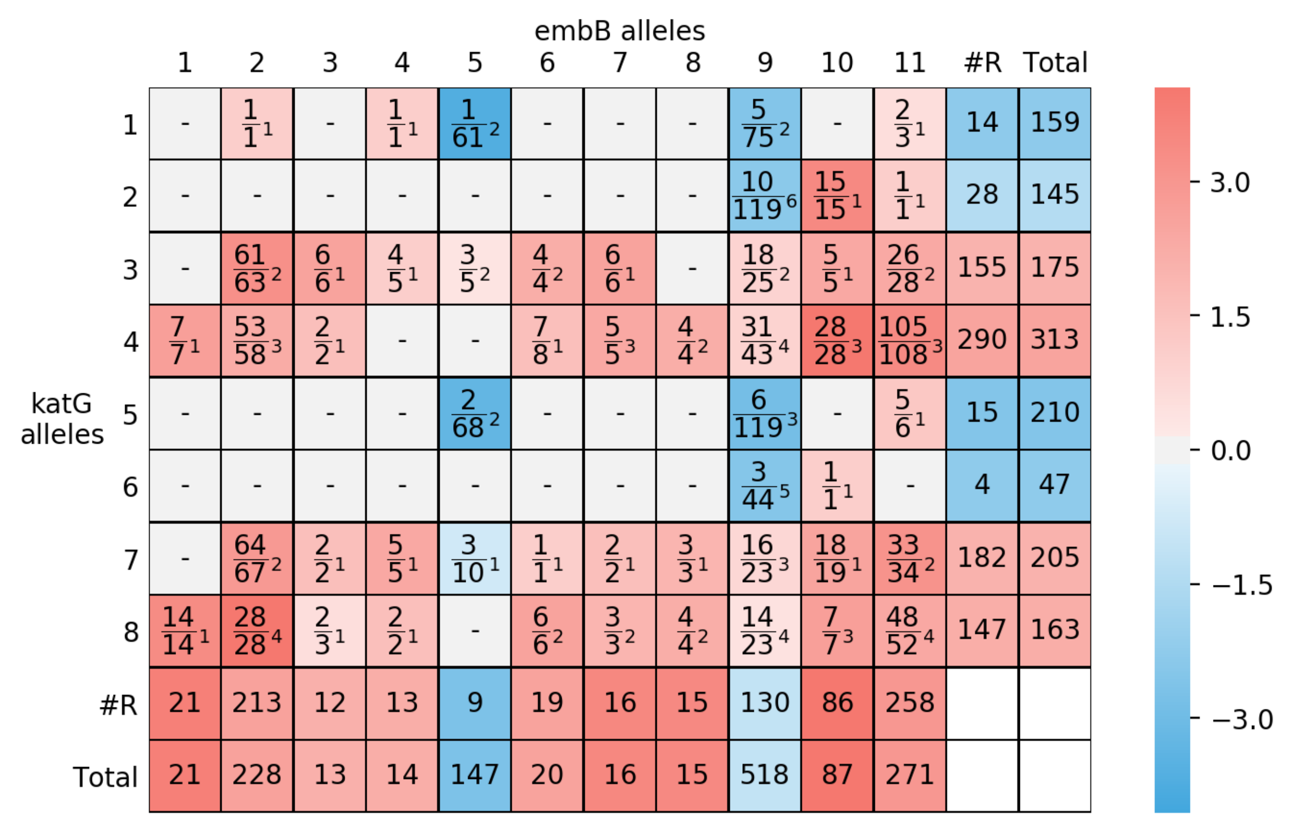
embB alleles
1 2 3 4 5 6 7 8 9 10 11 #R Total
katG alleles
1: - | 1/1 1 | - | 1/1 1 | 1/61 2 | - | - | - | 5/75 2 | - | 2/3 1 | 14 | 159
2: - | - | - | - | - | - | - | - | 10/119 6 | 15/15 1 | 1/1 1 | 28 | 145
3: - | 61/63 2 | 6/6 1 | 4/5 1 | 3/5 2 | 4/4 2 | 6/6 1 | - | 18/25 2 | 5/5 1 | 26/28 2 | 155 | 175
4: 7/7 1 | 53/58 3 | 2/2 1 | - | - | 7/8 1 | 5/5 3 | 4/4 2 | 31/43 4 | 28/28 3 | 105/108 3 | 290 | 313
5: - | - | - | - | 2/68 2 | - | - | - | 6/119 3 | - | 5/6 1 | 15 | 210
6: - | - | - | - | - | - | - | - | 3/44 5 | 1/1 1 | - | 4 | 47
7: - | 64/67 2 | 2/2 1 | 5/5 1 | 3/10 1 | 1/1 1 | 2/2 1 | 3/3 1 | 16/23 3 | 18/19 1 | 33/34 2 | 182 | 205
8: 14/14 1 | 28/28 4 | 2/3 1 | 2/2 1 | - | 6/6 2 | 3/3 2 | 4/4 2 | 14/23 4 | 7/7 3 | 48/52 4 | 147 | 163
#R: 21 | 213 | 12 | 13 | 9 | 19 | 16 | 15 | 130 | 86 | 258
Total: 21 | 228 | 13 | 14 | 147 | 20 | 16 | 15 | 518 | 87 | 271
3.0
1.5
0.0
−1.5
−3.0

pncA alleles \ embB alleles	1	2	3	4	5	6	7	8	9	10	11	#R	Total
1	-	$\frac{4}{4}^{1}$	-	$\frac{2}{2}^{1}$	-	$\frac{1}{1}^{1}$	-	-	-	-	-	9	11
10	$\frac{14}{14}^{1}$	$\frac{26}{34}^{5}$	$\frac{1}{1}^{1}$	$\frac{5}{5}^{2}$	$\frac{7}{141}^{2}$	$\frac{6}{7}^{2}$	$\frac{2}{2}^{2}$	$\frac{3}{3}^{2}$	$\frac{76}{412}^{6}$	$\frac{12}{13}^{3}$	$\frac{21}{23}^{2}$	212	740
11	-	$\frac{11}{11}^{1}$	-	-	-	-	-	-	-	-	-	11	11
12	-	$\frac{4}{4}^{1}$	-	-	-	-	$\frac{3}{3}^{1}$	-	$\frac{3}{4}^{1}$	-	$\frac{6}{7}^{1}$	19	22
13	-	-	-	-	-	-	-	-	-	$\frac{16}{16}^{1}$	-	16	16
14	-	-	-	-	-	-	-	-	-	$\frac{21}{21}^{2}$	-	21	21
2	-	-	$\frac{2}{2}^{1}$	$\frac{1}{1}^{1}$	-	-	-	-	$\frac{2}{2}^{1}$	-	$\frac{2}{2}^{1}$	11	11
3	-	$\frac{17}{17}^{1}$	-	-	-	-	-	-	-	-	$\frac{19}{19}^{1}$	38	38
4	-	-	-	-	-	-	-	-	-	-	$\frac{17}{17}^{1}$	17	17
5	-	$\frac{21}{21}^{1}$	-	-	-	-	-	-	-	$\frac{1}{1}^{1}$	$\frac{1}{1}^{1}$	24	24
6	-	$\frac{1}{1}^{1}$	-	-	-	-	$\frac{4}{4}^{1}$	$\frac{3}{3}^{1}$	$\frac{1}{1}^{1}$	$\frac{2}{2}^{2}$	$\frac{4}{4}^{2}$	16	16
7	-	-	-	-	-	-	-	-	-	-	$\frac{13}{14}^{1}$	14	15
8	-	$\frac{1}{1}^{1}$	-	-	-	-	-	-	$\frac{2}{2}^{1}$	$\frac{10}{10}^{1}$	-	15	15
9	-	$\frac{21}{21}^{1}$	-	-	-	-	-	-	-	-	-	22	22
#R	21	213	12	13	9	19	16	15	130	86	258		
Total	21	228	13	14	147	20	16	15	518	87	271		

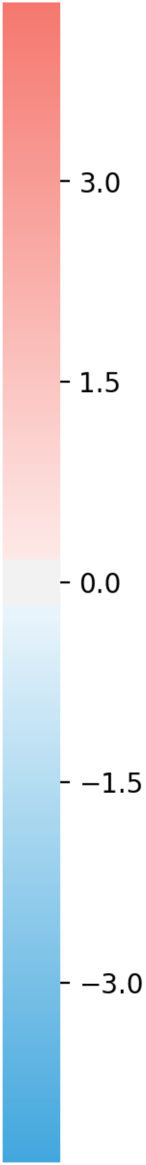

pncA alleles

rpoB alleles	1	2	3	4	5	6	7	8	9	10	11	12	13	14	#R	Total
1	-	-	-	-	-	-	-	-	$\frac{1}{1}$ 1	$\frac{10}{10}$ 3	-	-	-	$\frac{20}{20}$ 1	39	39
10	-	$\frac{3}{3}$ 2	-	-	-	$\frac{3}{3}$ 1	$\frac{1}{1}$ 1	-	-	$\frac{13}{14}$ 3	-	$\frac{16}{17}$ 1	-	-	106	112
11	-	-	-	-	-	-	-	-	-	$\frac{5}{7}$ 3	-	-	-	-	12	17
2	-	-	-	-	-	-	-	-	$\frac{21}{21}$ 1	-	-	-	-	-	22	22
3	$\frac{5}{7}$ 2	$\frac{5}{5}$ 2	$\frac{38}{38}$ 1	$\frac{17}{17}$ 1	$\frac{23}{23}$ 1	$\frac{8}{8}$ 3	$\frac{6}{6}$ 2	$\frac{13}{13}$ 2	-	$\frac{105}{114}$ 5	$\frac{11}{11}$ 1	$\frac{1}{1}$ 1	$\frac{16}{16}$ 1	$\frac{1}{1}$ 1	443	466
4	-	-	-	-	-	-	$\frac{1}{1}$ 1	-	-	$\frac{21}{490}$ 6	-	-	-	-	30	549
5	-	-	-	-	-	-	-	-	-	$\frac{5}{5}$ 3	-	-	-	-	15	21
6	-	-	-	-	-	$\frac{2}{2}$ 1	-	-	-	$\frac{2}{2}$ 1	-	-	-	-	11	11
7	-	-	-	-	-	-	-	-	-	$\frac{7}{8}$ 3	-	-	-	-	13	16
8	-	-	-	-	-	-	-	-	-	$\frac{9}{12}$ 3	-	-	-	-	18	21
9	-	-	-	-	-	-	-	-	-	-	-	-	-	-	64	64
#R	9	11	38	17	24	16	14	15	22	212	11	19	16	21		
Total	11	11	38	17	24	16	15	15	22	740	11	22	16	21		

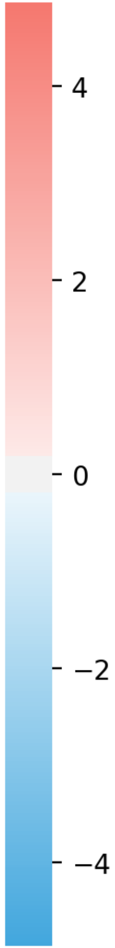

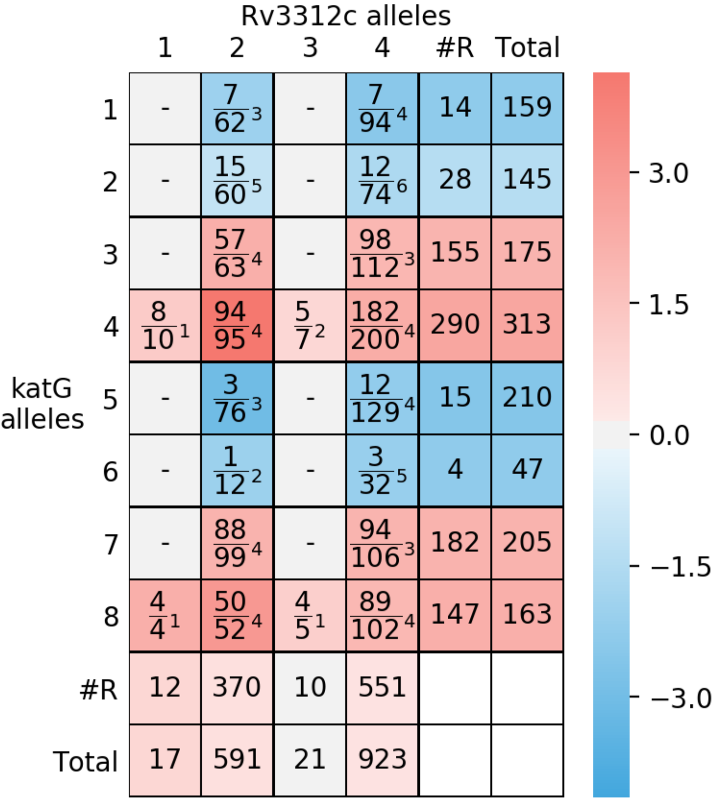

Rv3312c alleles
1
2
3
4
#R
Total
katG alleles
1
- 7/62 3 - 7/94 4 14 159
2
- 15/60 5 - 12/74 6 28 145
3
- 57/63 4 - 98/112 3 155 175
4
8/10 1 94/95 4 5/7 2 182/200 4 290 313
5
- 3/76 3 - 12/129 4 15 210
6
- 1/12 2 - 3/32 5 4 47
7
- 88/99 4 - 94/106 3 182 205
8
4/4 1 50/52 4 4/5 1 89/102 4 147 163
#R
12 370 10 551
Total
17 591 21 923
3.0
1.5
0.0
−1.5
−3.0

katG alleles \ gid alleles	1	2	3	4	5	6	7	8	9	10	11	12	13	#R	Total
1	-	$\frac{1}{5}^{1}$	$\frac{4}{16}^{1}$	$\frac{1}{4}^{1}$	-	$\frac{1}{6}^{1}$	-	-	$\frac{4}{65}^{4}$	$\frac{2}{8}^{1}$	-	-	-	14	159
2	-	-	-	$\frac{4}{8}$	$\frac{1}{2}$	-	-	-	$\frac{20}{92}^{5}$	-	-	-	$\frac{2}{31}^{2}$	28	145
3	-	$\frac{1}{1}^{1}$	$\frac{25}{28}^{1}$	$\frac{11}{12}^{1}$	$\frac{2}{2}^{2}$	$\frac{1}{2}$	$\frac{68}{74}^{1}$	-	$\frac{9}{17}^{2}$	$\frac{1}{2}$	$\frac{28}{28}^{1}$	-	-	155	175
4	$\frac{87}{89}^{1}$	-	-	$\frac{15}{19}^{4}$	$\frac{5}{5}^{1}$	-	-	-	$\frac{73}{79}^{4}$	-	-	$\frac{8}{12}^{1}$	$\frac{31}{34}^{2}$	290	313
5	-	-	$\frac{3}{33}^{1}$	$\frac{1}{3}^{1}$	-	-	$\frac{5}{52}^{1}$	-	$\frac{4}{80}^{3}$	$\frac{2}{6}^{1}$	-	-	-	15	210
6	-	-	-	$\frac{1}{5}^{2}$	-	-	-	-	$\frac{3}{32}^{4}$	-	-	-	-	4	47
7	-	-	$\frac{52}{58}^{1}$	$\frac{8}{8}^{2}$	-	$\frac{3}{3}^{1}$	$\frac{72}{79}^{2}$	-	$\frac{5}{11}^{2}$	$\frac{1}{1}^{1}$	$\frac{25}{25}^{1}$	-	-	182	205
8	$\frac{37}{41}^{1}$	-	-	$\frac{9}{10}^{2}$	$\frac{1}{1}^{1}$	-	-	-	$\frac{58}{64}^{3}$	-	-	$\frac{5}{5}^{1}$	$\frac{6}{7}^{2}$	147	163
#R	125	5	94	60	9	9	171	0	212	6	53	13	40		
Total	132	14	152	82	15	30	283	11	484	17	53	18	81		

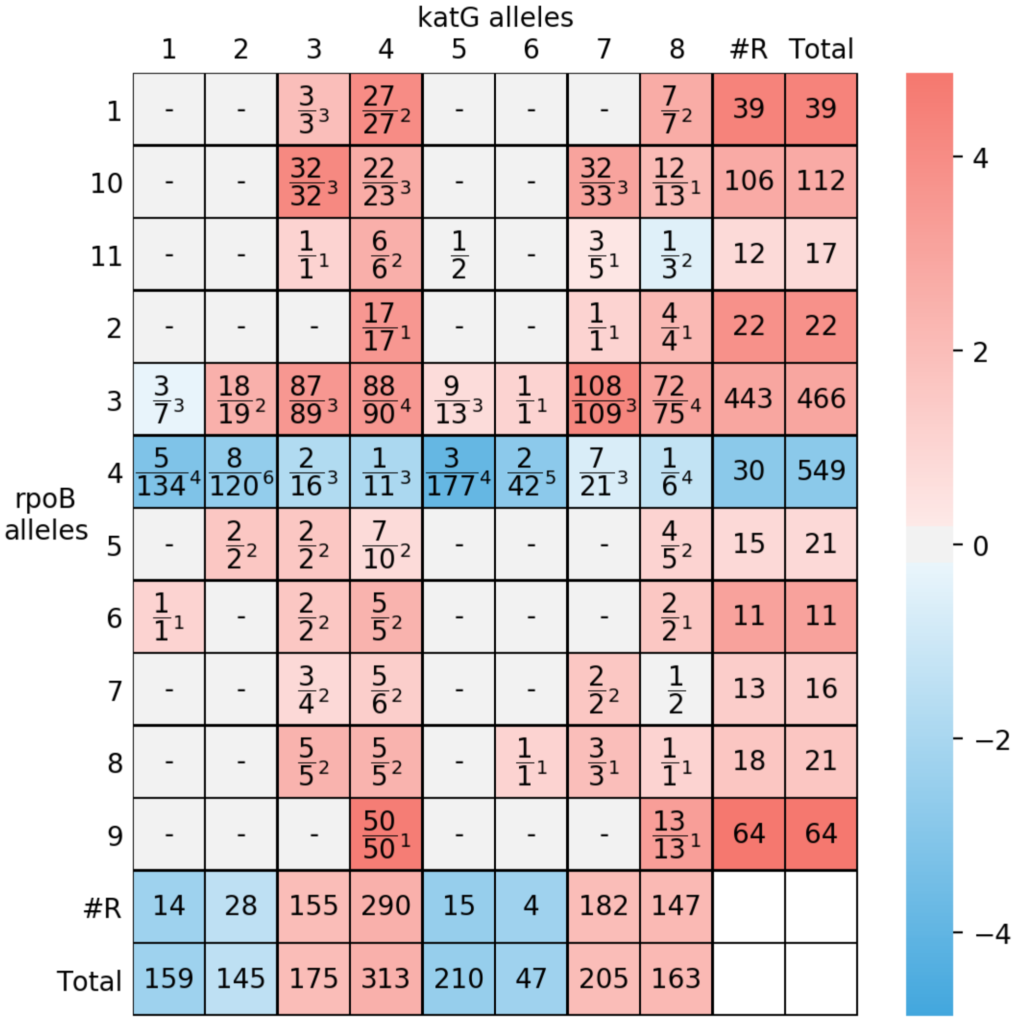
katG alleles
1 2 3 4 5 6 7 8 #R Total
rpoB alleles
1 - - 3/3 3 27/27 2 - - - 7/7 2 39 39
10 - - 32/32 3 22/23 3 - - 32/33 3 12/13 1 106 112
11 - - 1/1 1 6/6 2 1/2 - 3/5 1 1/3 2 12 17
2 - - - 17/17 1 - - 1/1 1 4/4 1 22 22
3 3/7 3 18/19 2 87/89 3 88/90 4 9/13 3 1/1 1 108/109 3 72/75 4 443 466
4 5/134 4 8/120 6 2/16 3 1/11 3 3/177 4 2/42 5 7/21 3 1/6 4 30 549
5 - 2/2 2 2/2 2 7/10 2 - - - 4/5 2 15 21
6 1/1 1 - 2/2 2 5/5 2 - - - 2/2 1 11 11
7 - - 3/4 2 5/6 2 - - 2/2 2 1/2 13 16
8 - - 5/5 2 5/5 2 - 1/1 1 3/3 1 1/1 1 18 21
9 - - - 50/50 1 - - - 13/13 1 64 64
#R 14 28 155 290 15 4 182 147
Total 159 145 175 313 210 47 205 163
4
2
0
−2
−4

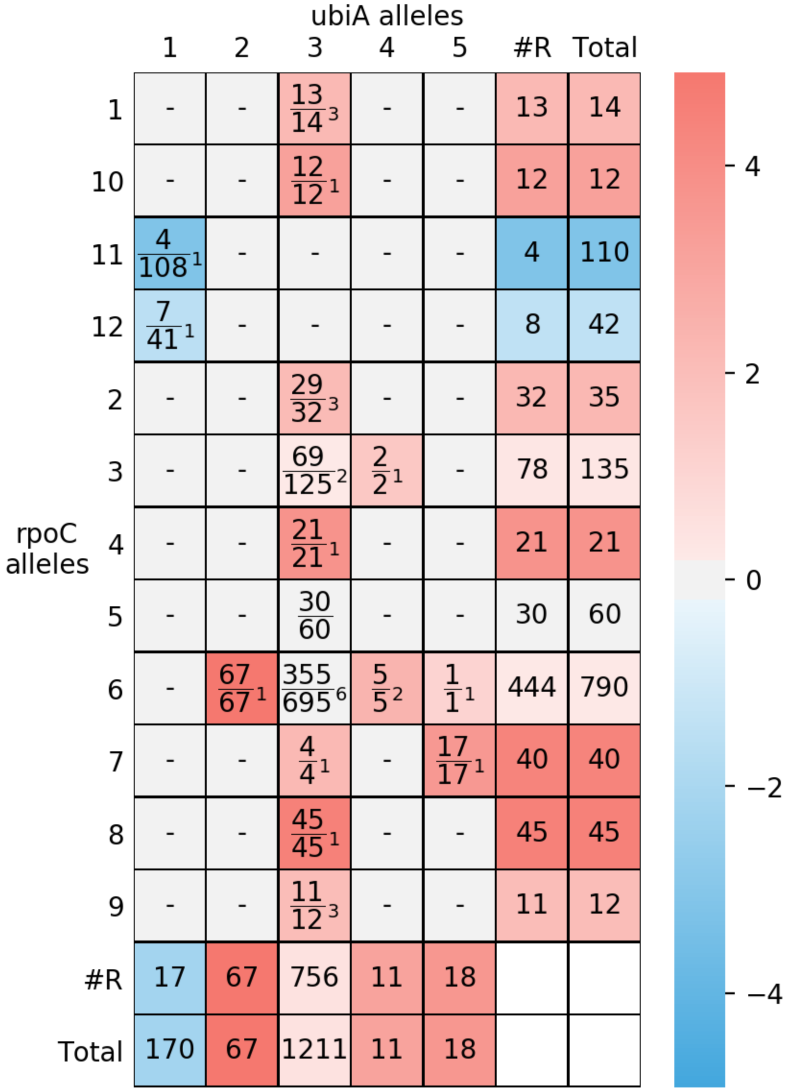

ubiA alleles
1
2
3
4
5
#R
Total
rpoC alleles
1
- - 13/14 3 - - 13 14
10
- - 12/12 1 - - 12 12
11
4/108 1 - - - - 4 110
12
7/41 1 - - - - 8 42
2
- - 29/32 3 - - 32 35
3
- - 69/125 2 2/2 1 - 78 135
4
- - 21/21 1 - - 21 21
5
- - 30/60 - - 30 60
6
- 67/67 1 355/695 6 5/5 2 1/1 1 444 790
7
- - 4/4 1 - 17/17 1 40 40
8
- - 45/45 1 - - 45 45
9
- - 11/12 3 - - 11 12
#R
17 67 756 11 18
Total
170 67 1211 11 18
4
2
0
−2
−4

katG alleles \ gyrA alleles	1	2	3	4	5	6	7	8	9	10	11	12	13	#R	Total
1	-	-	$\frac{1}{2}$	-	$\frac{6}{82}^{3}$	-	$\frac{2}{53}^{2}$	-	-	-	-	-	-	14	159
2	-	-	$\frac{1}{1}^{1}$	$\frac{1}{17}^{1}$	$\frac{21}{87}^{6}$	-	-	-	-	$\frac{1}{1}^{1}$	-	-	-	28	145
3	-	$\frac{2}{2}^{1}$	$\frac{8}{8}^{1}$	-	$\frac{88}{100}^{3}$	-	$\frac{6}{12}$	-	$\frac{20}{21}^{1}$	$\frac{6}{6}^{1}$	$\frac{5}{5}^{2}$	$\frac{10}{10}^{1}$	-	155	175
4	$\frac{6}{7}^{1}$	$\frac{1}{1}^{1}$	$\frac{2}{2}^{1}$	$\frac{21}{22}^{1}$	$\frac{96}{109}^{4}$	$\frac{14}{14}^{1}$	-	$\frac{44}{47}^{1}$	$\frac{11}{11}^{2}$	$\frac{1}{1}^{1}$	$\frac{5}{5}^{2}$	$\frac{5}{6}^{2}$	$\frac{64}{64}^{1}$	290	313
5	-	-	$\frac{1}{2}$	-	$\frac{10}{117}^{2}$	-	$\frac{2}{62}^{2}$	-	-	-	-	-	-	15	210
6	-	-	-	$\frac{1}{12}^{1}$	$\frac{3}{24}^{4}$	-	-	-	-	-	-	-	-	4	47
7	-	$\frac{4}{4}^{1}$	$\frac{10}{10}^{1}$	-	$\frac{99}{113}^{3}$	-	$\frac{4}{9}^{1}$	-	$\frac{22}{23}^{1}$	$\frac{5}{5}^{1}$	$\frac{2}{2}^{1}$	$\frac{23}{23}^{1}$	-	182	205
8	$\frac{3}{4}^{1}$	-	$\frac{4}{4}^{1}$	$\frac{15}{17}^{1}$	$\frac{55}{62}^{3}$	$\frac{6}{6}^{1}$	-	$\frac{26}{31}^{1}$	$\frac{1}{1}^{1}$	-	-	$\frac{2}{2}^{1}$	$\frac{15}{15}^{1}$	147	163
#R	10	9	33	58	431	21	15	70	61	15	17	43	80		
Total	24	10	35	88	769	22	140	95	71	16	18	48	80		

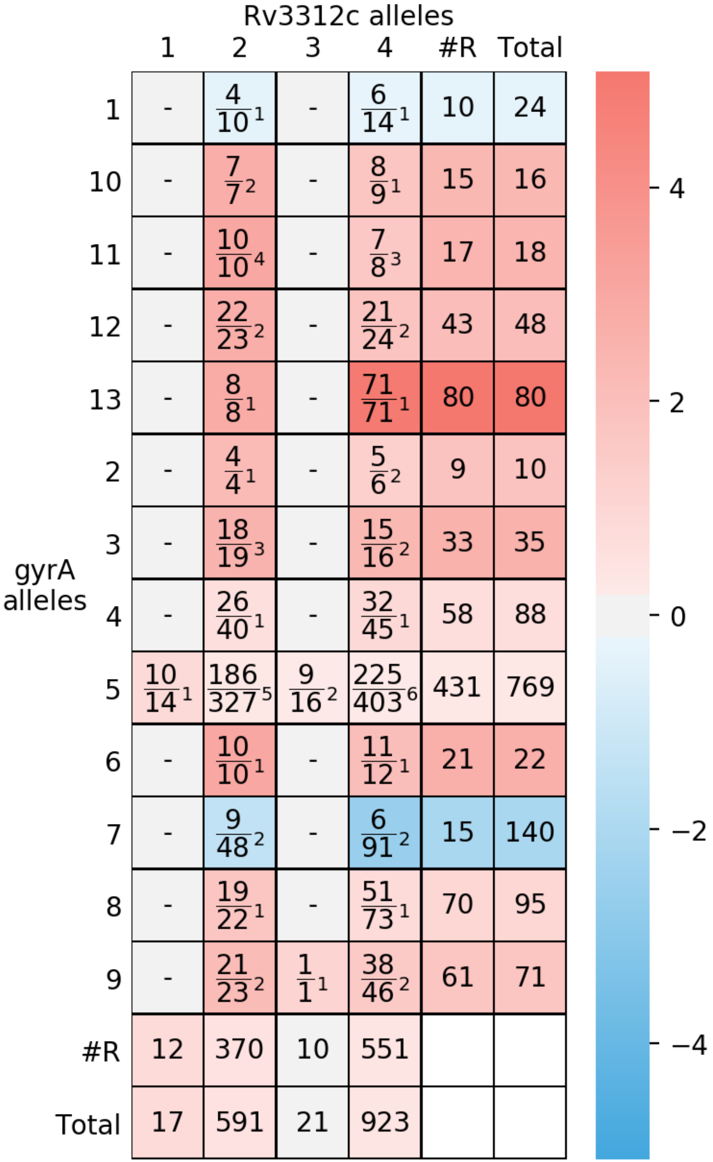
Rv3312c alleles
1
2
3
4
#R
Total
gyrA alleles
1
-
4/10 1
-
6/14 1
10
24
10
-
7/7 2
-
8/9 1
15
16
11
-
10/10 4
-
7/8 3
17
18
12
-
22/23 2
-
21/24 2
43
48
13
-
8/8 1
-
71/71 1
80
80
2
-
4/4 1
-
5/6 2
9
10
3
-
18/19 3
-
15/16 2
33
35
4
-
26/40 1
-
32/45 1
58
88
5
10/14 1
186/327 5
9/16 2
225/403 6
431
769
6
-
10/10 1
-
11/12 1
21
22
7
-
9/48 2
-
6/91 2
15
140
8
-
19/22 1
-
51/73 1
70
95
9
-
21/23 2
1/1 1
38/46 2
61
71
#R
12
370
10
551
Total
17
591
21
923
4
2
0
−2
−4

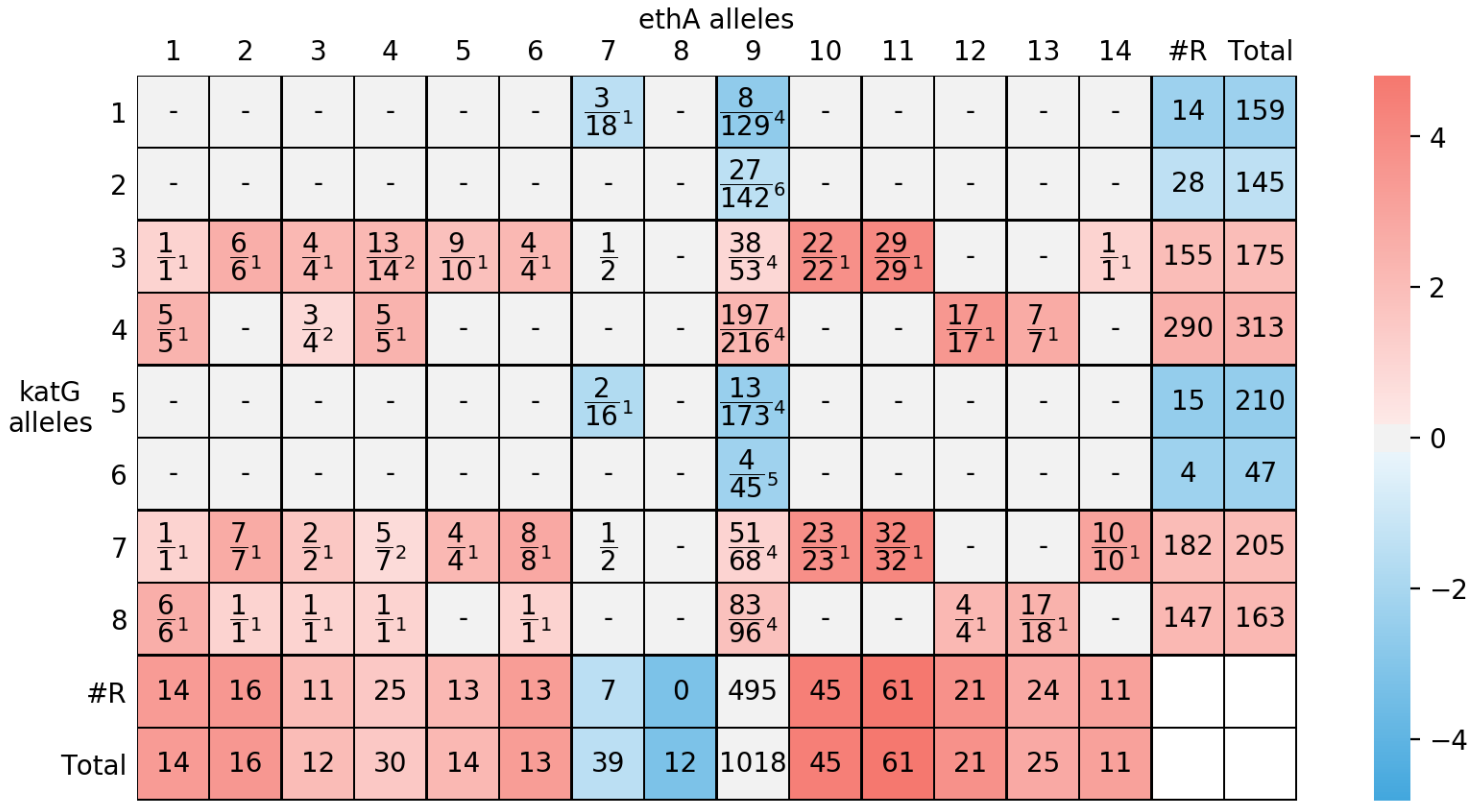

katG alleles \ ethA alleles	1	2	3	4	5	6	7	8	9	10	11	12	13	14	#R	Total
1	-	-	-	-	-	-	3/18 [1]	-	8/129 [4]	-	-	-	-	-	14	159
2	-	-	-	-	-	-	-	-	27/142 [6]	-	-	-	-	-	28	145
3	1/1 [1]	6/6 [1]	4/4 [1]	13/14 [2]	9/10 [1]	4/4 [1]	1/2	-	38/53 [4]	22/22 [1]	29/29 [1]	-	-	1/1 [1]	155	175
4	5/5 [1]	-	3/4 [2]	5/5 [1]	-	-	-	-	197/216 [4]	-	-	17/17 [1]	7/7 [1]	-	290	313
5	-	-	-	-	-	-	2/16 [1]	-	13/173 [4]	-	-	-	-	-	15	210
6	-	-	-	-	-	-	-	-	4/45 [5]	-	-	-	-	-	4	47
7	1/1 [1]	7/7 [1]	2/2 [1]	5/7 [2]	4/4 [1]	8/8 [1]	1/2	-	51/68 [4]	23/23 [1]	32/32 [1]	-	-	10/10 [1]	182	205
8	6/6 [1]	1/1 [1]	1/1 [1]	1/1 [1]	-	1/1 [1]	-	-	83/96 [4]	-	-	4/4 [1]	17/18 [1]	-	147	163
#R	14	16	11	25	13	13	7	0	495	45	61	21	24	11		
Total	14	16	12	30	14	13	39	12	1018	45	61	21	25	11		

gid alleles

ethA alleles	1	2	3	4	5	6	7	8	9	10	11	12	13	#R	Total
1	-	-	$\frac{1}{1}^{1}$	$\frac{1}{1}^{1}$	-	-	$\frac{1}{1}^{1}$	-	-	-	-	$\frac{1}{1}^{1}$	-	14	14
10	-	-	$\frac{9}{9}^{1}$	-	-	-	$\frac{36}{36}^{1}$	-	-	-	-	-	-	45	45
11	-	-	-	-	-	-	-	-	-	-	$\frac{53}{53}^{1}$	-	-	61	61
12	-	-	-	$\frac{1}{1}^{1}$	-	-	-	-	$\frac{20}{20}^{1}$	-	-	-	-	21	21
13	-	-	-	-	-	-	-	-	$\frac{24}{25}^{1}$	-	-	-	-	24	25
14	-	-	$\frac{11}{11}^{1}$	-	-	-	-	-	-	-	-	-	-	11	11
2	-	-	$\frac{3}{3}^{1}$	-	-	-	$\frac{12}{12}^{1}$	-	-	-	-	-	-	16	16
3	-	-	$\frac{1}{1}^{1}$	$\frac{5}{5}^{3}$	-	-	$\frac{4}{4}^{1}$	-	-	-	-	-	-	11	12
4	-	-	$\frac{5}{5}^{1}$	$\frac{3}{3}^{1}$	-	-	$\frac{11}{13}^{1}$	-	$\frac{2}{4}$	-	-	-	$\frac{1}{1}^{1}$	25	30
5	-	-	$\frac{3}{3}^{1}$	-	-	-	$\frac{10}{11}^{1}$	-	-	-	-	-	-	13	14
6	-	-	$\frac{6}{6}^{1}$	-	-	-	$\frac{6}{6}^{1}$	-	$\frac{1}{1}^{1}$	-	-	-	-	13	13
7	-	-	-	-	-	-	-	-	-	$\frac{5}{16}^{1}$	-	-	-	7	39
8	-	-	-	-	-	-	-	-	-	-	-	-	-	0	12
9	$\frac{125}{132}^{1}$	$\frac{5}{14}^{1}$	$\frac{27}{71}^{1}$	$\frac{37}{54}^{4}$	$\frac{7}{11}^{4}$	$\frac{9}{29}^{1}$	$\frac{50}{152}^{2}$	-	$\frac{126}{365}^{6}$	-	-	$\frac{9}{13}^{2}$	$\frac{26}{66}^{2}$	495	1018
#R	125	5	94	60	9	9	171	0	212	6	53	13	40		
Total	132	14	152	82	15	30	283	11	484	17	53	18	81		

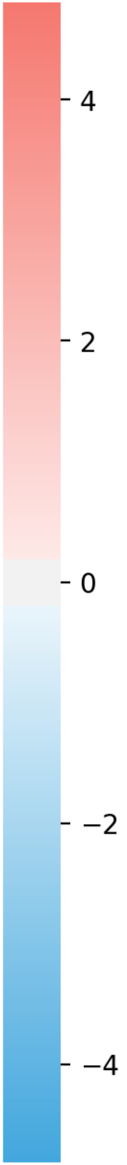

rpoC alleles

katG alleles	1	2	3	4	5	6	7	8	9	10	11	12	#R	Total
1	-	-	-	-	-	$\frac{9}{76}$ 2	-	-	-	-	-	$\frac{1}{16}$ 1	14	159
2	-	-	$\frac{3}{33}$ 2	$\frac{15}{15}$ 1	-	$\frac{7}{90}$ 5	-	-	-	-	-	-	28	145
3	$\frac{5}{5}$ 2	$\frac{3}{3}$ 1	$\frac{1}{1}$ 1	-	$\frac{8}{11}$ 1	$\frac{61}{69}$ 2	-	$\frac{22}{22}$ 1	-	$\frac{1}{1}$ 1	$\frac{2}{7}$ 1	$\frac{1}{3}$ 1	155	175
4	$\frac{3}{3}$ 1	$\frac{8}{8}$ 1	$\frac{52}{59}$ 2	-	-	$\frac{156}{170}$ 4	$\frac{25}{25}$ 1	-	$\frac{7}{7}$ 1	$\frac{1}{1}$ 1	-	-	290	313
5	-	-	-	-	$\frac{1}{15}$ 2	$\frac{5}{106}$ 2	-	-	-	-	$\frac{1}{50}$ 1	$\frac{1}{16}$ 1	15	210
6	-	-	$\frac{1}{14}$ 2	$\frac{1}{1}$ 1	-	$\frac{1}{30}$ 4	-	-	-	-	-	-	4	47
7	$\frac{1}{1}$ 2	$\frac{4}{4}$ 1	-	-	$\frac{17}{20}$ 1	$\frac{73}{82}$ 2	-	$\frac{23}{23}$ 1	$\frac{2}{2}$ 1	$\frac{10}{10}$ 1	$\frac{1}{7}$ 1	$\frac{4}{5}$ 1	182	205
8	$\frac{4}{4}$ 1	$\frac{14}{14}$ 1	$\frac{17}{20}$ 2	-	-	$\frac{70}{80}$ 3	$\frac{14}{14}$ 1	-	$\frac{1}{2}$	-	-	-	147	163
#R	13	32	78	21	30	444	40	45	11	12	4	8		
Total	14	35	135	21	60	790	40	45	12	12	110	42		

4
2
0
−2
−4

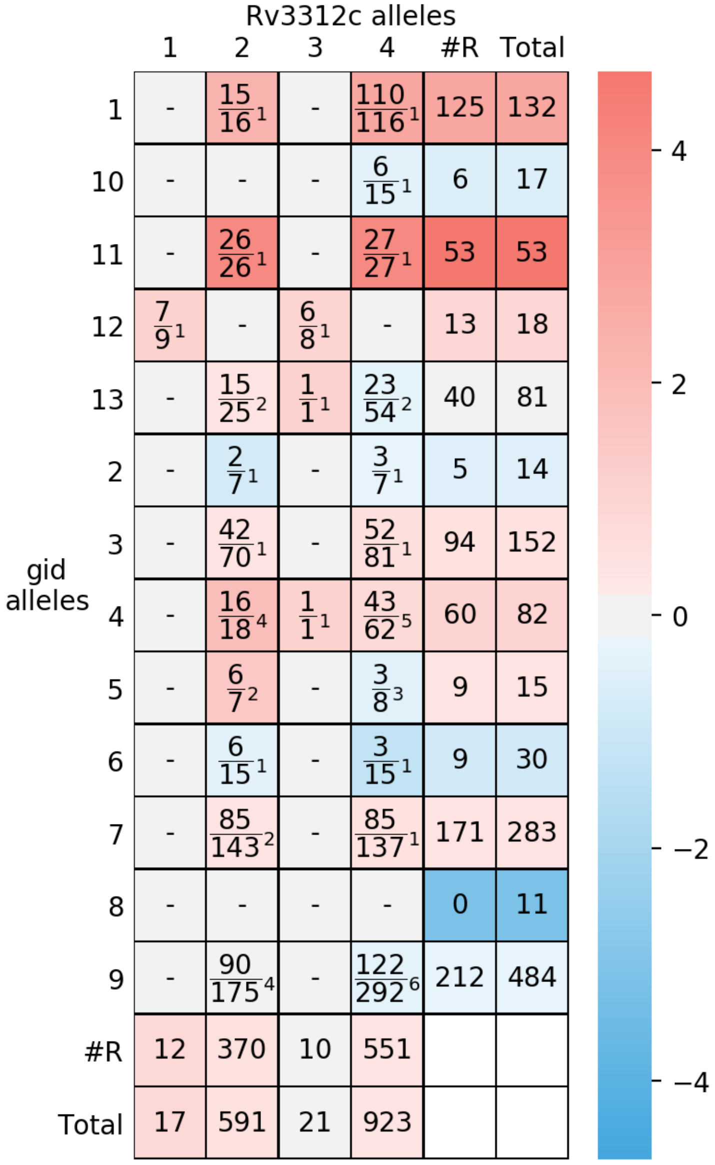
Rv3312c alleles
1 2 3 4 #R Total
gid alleles
1: - | 15/16 1 | - | 110/116 1 | 125 | 132
10: - | - | - | 6/15 1 | 6 | 17
11: - | 26/26 1 | - | 27/27 1 | 53 | 53
12: 7/9 1 | - | 6/8 1 | - | 13 | 18
13: - | 15/25 2 | 1/1 1 | 23/54 2 | 40 | 81
2: - | 2/7 1 | - | 3/7 1 | 5 | 14
3: - | 42/70 1 | - | 52/81 1 | 94 | 152
4: - | 16/18 4 | 1/1 1 | 43/62 5 | 60 | 82
5: - | 6/7 2 | - | 3/8 3 | 9 | 15
6: - | 6/15 1 | - | 3/15 1 | 9 | 30
7: - | 85/143 2 | - | 85/137 1 | 171 | 283
8: - | - | - | - | 0 | 11
9: - | 90/175 4 | - | 122/292 6 | 212 | 484
#R: 12 | 370 | 10 | 551
Total: 17 | 591 | 21 | 923
4
2
0
−2
−4

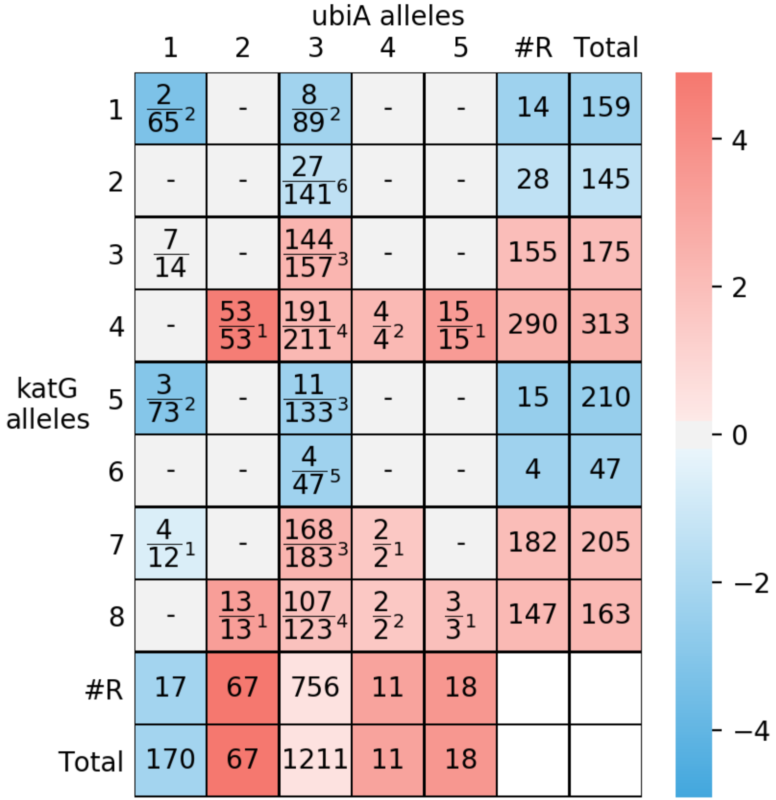

ubiA alleles
1
2
3
4
5
#R
Total
katG alleles
1
2/65 2
-
8/89 2
-
-
14
159
2
-
-
27/141 6
-
-
28
145
3
7/14
-
144/157 3
-
-
155
175
4
-
53/53 1
191/211 4
4/4 2
15/15 1
290
313
5
3/73 2
-
11/133 3
-
-
15
210
6
-
-
4/47 5
-
-
4
47
7
4/12 1
-
168/183 3
2/2 1
-
182
205
8
-
13/13 1
107/123 4
2/2 2
3/3 1
147
163
#R
17
67
756
11
18
Total
170
67
1211
11
18
4
2
0
−2
−4

rpoB alleles \ gyrA alleles	1	2	3	4	5	6	7	8	9	10	11	12	13	#R	Total
1	-	-	-	$\frac{1}{1}^{1}$	$\frac{11}{11}^{3}$	$\frac{3}{3}^{1}$	$\frac{1}{1}^{1}$	$\frac{9}{9}^{1}$	-	-	-	-	$\frac{8}{8}^{1}$	39	39
10	-	$\frac{2}{2}^{1}$	-	$\frac{17}{18}^{1}$	$\frac{49}{52}^{3}$	-	$\frac{3}{3}^{2}$	$\frac{1}{1}^{1}$	$\frac{8}{10}^{2}$	$\frac{8}{8}^{1}$	$\frac{4}{4}^{2}$	$\frac{4}{4}^{1}$	$\frac{1}{1}^{1}$	106	112
11	-	-	-	-	$\frac{7}{10}^{2}$	-	$\frac{1}{1}^{1}$	-	-	-	$\frac{3}{4}^{1}$	-	-	12	17
2	-	-	-	-	$\frac{11}{11}^{1}$	-	-	-	$\frac{4}{4}^{2}$	$\frac{1}{1}^{1}$	-	$\frac{5}{5}^{1}$	-	22	22
3	$\frac{9}{12}^{1}$	$\frac{3}{4}^{2}$	$\frac{24}{25}^{2}$	$\frac{22}{23}^{1}$	$\frac{240}{247}^{5}$	$\frac{18}{18}^{1}$	$\frac{5}{5}^{2}$	$\frac{21}{24}^{1}$	$\frac{32}{34}^{2}$	$\frac{3}{4}^{1}$	$\frac{3}{3}^{1}$	$\frac{29}{31}^{1}$	$\frac{5}{5}^{1}$	443	466
4	-	-	-	$\frac{3}{30}^{1}$	$\frac{20}{314}^{6}$	-	$\frac{3}{111}^{2}$	$\frac{1}{17}^{1}$	-	-	-	-	-	30	549
5	-	-	-	-	$\frac{2}{6}^{3}$	-	$\frac{1}{1}^{1}$	$\frac{8}{8}^{1}$	-	-	$\frac{1}{1}^{1}$	-	-	15	21
6	-	-	-	-	$\frac{5}{5}^{3}$	-	-	$\frac{4}{4}^{1}$	$\frac{1}{1}^{1}$	-	-	-	-	11	11
7	-	-	-	-	$\frac{7}{9}^{3}$	-	-	$\frac{1}{1}^{1}$	-	-	$\frac{1}{1}^{1}$	-	-	13	16
8	-	-	-	-	$\frac{12}{15}^{3}$	-	-	$\frac{2}{2}^{1}$	$\frac{1}{1}^{1}$	$\frac{1}{1}^{1}$	-	$\frac{1}{1}^{1}$	-	18	21
9	-	-	-	-	-	-	-	-	-	-	-	-	$\frac{64}{64}^{1}$	64	64
#R	10	9	33	58	431	21	15	70	61	15	17	43	80		
Total	24	10	35	88	769	22	140	95	71	16	18	48	80		

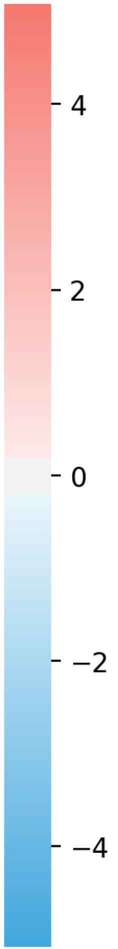

Supplement: Supplementary file 8 — Supplementary Data 5 [file 41467_2018_6634_MOESM8_ESM.zip › Supplementary Data 5/MDR_epistasis.pdf]
