# Supplementary material for: Machine learning and structural analysis of Mycobacterium tuberculosis pan-genome identifies genetic signatures of antibiotic resistance
Source: Nat Commun. 2018 Oct 17;9:4306. doi: 10.1038/s41467-018-06634-y (PMC6193043; doi:10.1038/s41467-018-06634-y)

Rv2680 alleles

Rv1877 alleles	1	2	3	4	5	6	7	8	9	10	#R	Total
1	-	-	$\frac{1}{1}{}^{1}$	$\frac{7}{7}{}^{1}$	-	$\frac{26}{27}{}^{1}$	$\frac{2}{2}{}^{1}$	$\frac{9}{11}{}^{1}$	-	-	45	48
2	$\frac{6}{6}{}^{1}$	$\frac{2}{2}{}^{1}$	$\frac{1}{1}{}^{1}$	$\frac{33}{33}{}^{1}$	-	$\frac{15}{15}{}^{1}$	-	$\frac{3}{3}{}^{1}$	-	-	60	60
3	$\frac{3}{4}{}^{1}$	-	-	$\frac{1}{1}{}^{1}$	-	$\frac{10}{15}{}^{1}$	$\frac{1}{2}$	$\frac{3}{6}$	-	-	18	29
4	$\frac{3}{3}{}^{1}$	$\frac{1}{1}{}^{1}$	$\frac{2}{2}{}^{1}$	$\frac{6}{6}{}^{1}$	-	$\frac{10}{12}{}^{1}$	$\frac{1}{1}{}^{1}$	-	$\frac{11}{12}{}^{1}$	$\frac{54}{54}{}^{1}$	91	94
5	$\frac{40}{67}{}^{5}$	$\frac{2}{6}{}^{2}$	$\frac{62}{87}{}^{4}$	$\frac{76}{124}{}^{5}$	$\frac{8}{9}{}^{1}$	$\frac{249}{477}{}^{6}$	$\frac{63}{96}{}^{4}$	$\frac{167}{257}{}^{6}$	$\frac{18}{32}{}^{3}$	$\frac{21}{36}{}^{3}$	720	1224
6	-	-	-	-	-	$\frac{8}{31}{}^{1}$	-	$\frac{2}{5}{}^{1}$	-	-	10	42
#R	59	5	75	125	8	331	68	187	30	77		
Total	89	10	102	180	10	601	104	289	45	93		

Colour scale: 4, 2, 0, −2, −4

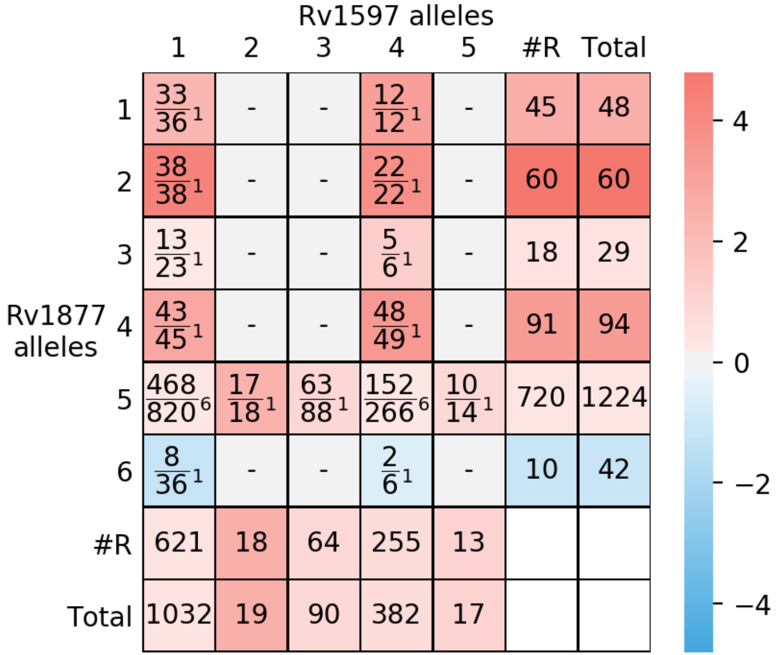

Rv1597 alleles
1 2 3 4 5 #R Total
Rv1877 alleles
1: 33/36 1, -, -, 12/12 1, -, 45, 48
2: 38/38 1, -, -, 22/22 1, -, 60, 60
3: 13/23 1, -, -, 5/6 1, -, 18, 29
4: 43/45 1, -, -, 48/49 1, -, 91, 94
5: 468/820 6, 17/18 1, 63/88 1, 152/266 6, 10/14 1, 720, 1224
6: 8/36 1, -, -, 2/6 1, -, 10, 42
#R: 621, 18, 64, 255, 13
Total: 1032, 19, 90, 382, 17
4
2
0
−2
−4

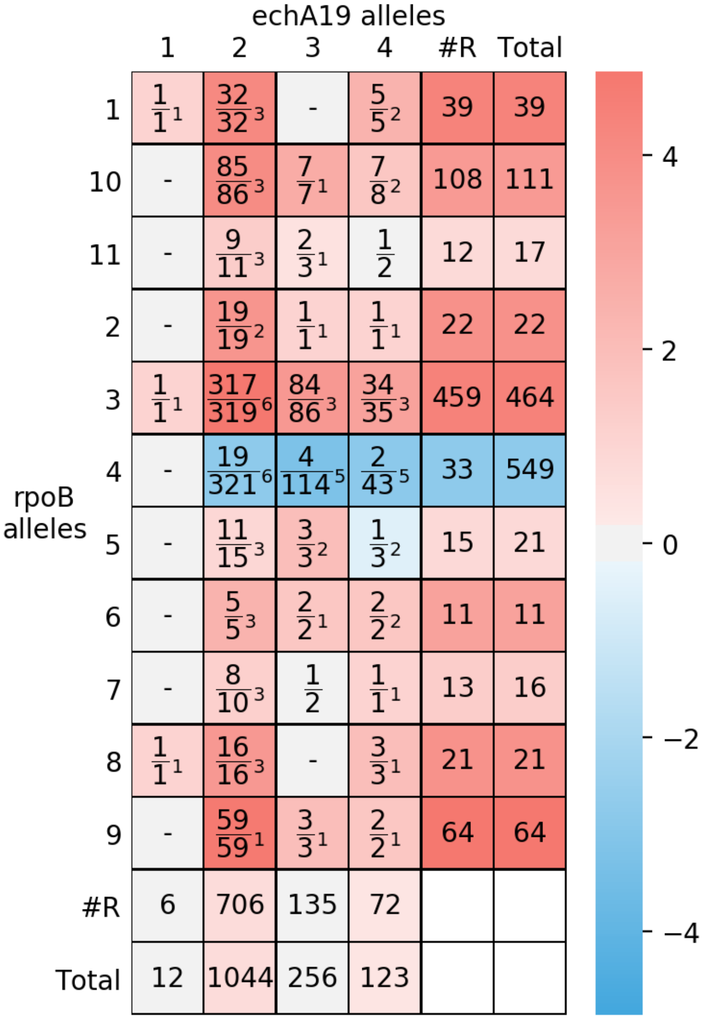

echA19 alleles
1 2 3 4 #R Total
rpoB alleles
1: 1/1 1 | 32/32 3 | - | 5/5 2 | 39 | 39
10: - | 85/86 3 | 7/7 1 | 7/8 2 | 108 | 111
11: - | 9/11 3 | 2/3 1 | 1/2 | 12 | 17
2: - | 19/19 2 | 1/1 1 | 1/1 1 | 22 | 22
3: 1/1 1 | 317/319 6 | 84/86 3 | 34/35 3 | 459 | 464
4: - | 19/321 6 | 4/114 5 | 2/43 5 | 33 | 549
5: - | 11/15 3 | 3/3 2 | 1/3 2 | 15 | 21
6: - | 5/5 3 | 2/2 1 | 2/2 2 | 11 | 11
7: - | 8/10 3 | 1/2 | 1/1 1 | 13 | 16
8: 1/1 1 | 16/16 3 | - | 3/3 1 | 21 | 21
9: - | 59/59 1 | 3/3 1 | 2/2 1 | 64 | 64
#R: 6 | 706 | 135 | 72
Total: 12 | 1044 | 256 | 123
4
2
0
−2
−4

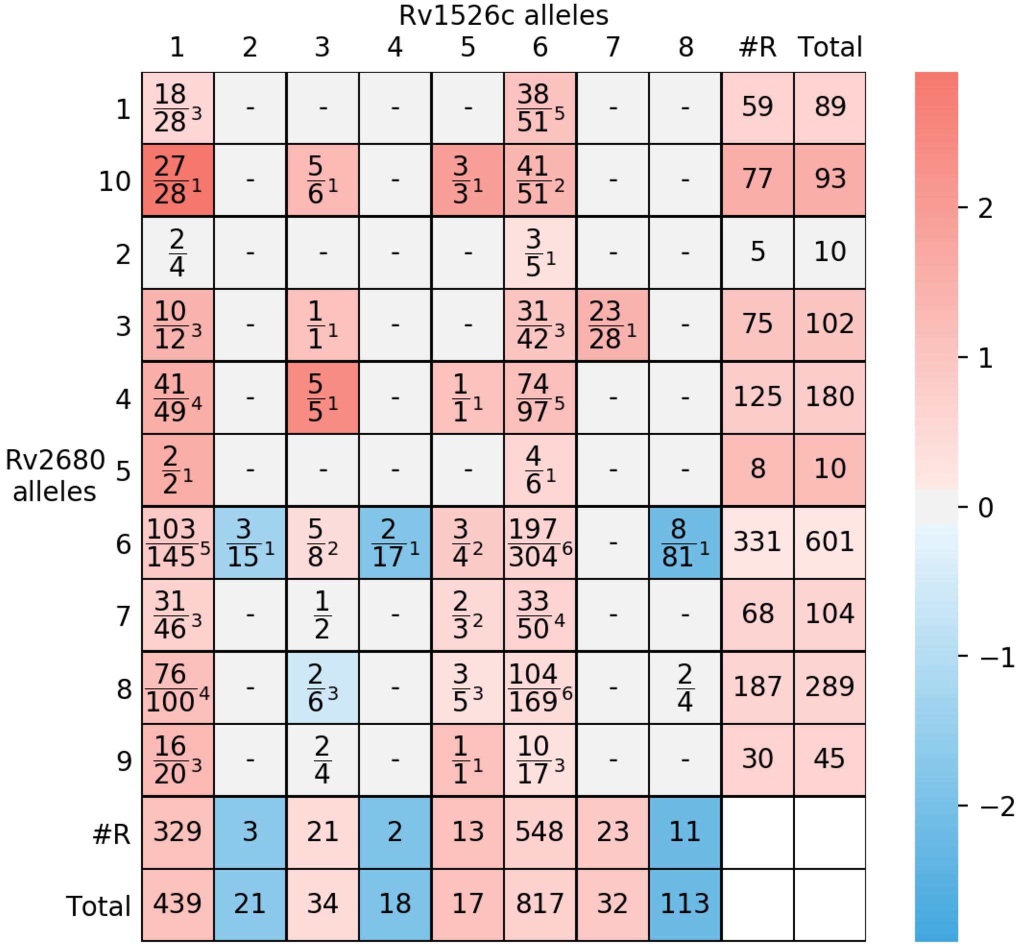
Rv1526c alleles
1 2 3 4 5 6 7 8 #R Total
Rv2680 alleles
1: 18/28 3, -, -, -, -, 38/51 5, -, -, 59, 89
10: 27/28 1, -, 5/6 1, -, 3/3 1, 41/51 2, -, -, 77, 93
2: 2/4, -, -, -, -, 3/5 1, -, -, 5, 10
3: 10/12 3, -, 1/1 1, -, -, 31/42 3, 23/28 1, -, 75, 102
4: 41/49 4, -, 5/5 1, -, 1/1 1, 74/97 5, -, -, 125, 180
5: 2/2 1, -, -, -, -, 4/6 1, -, -, 8, 10
6: 103/145 5, 3/15 1, 5/8 2, 2/17 1, 3/4 2, 197/304 6, -, 8/81 1, 331, 601
7: 31/46 3, -, 1/2, -, 2/3 2, 33/50 4, -, -, 68, 104
8: 76/100 4, -, 2/6 3, -, 3/5 3, 104/169 6, -, 2/4, 187, 289
9: 16/20 3, -, 2/4, -, 1/1 1, 10/17 3, -, -, 30, 45
#R: 329, 3, 21, 2, 13, 548, 23, 11
Total: 439, 21, 34, 18, 17, 817, 32, 113
2
1
0
−1
−2

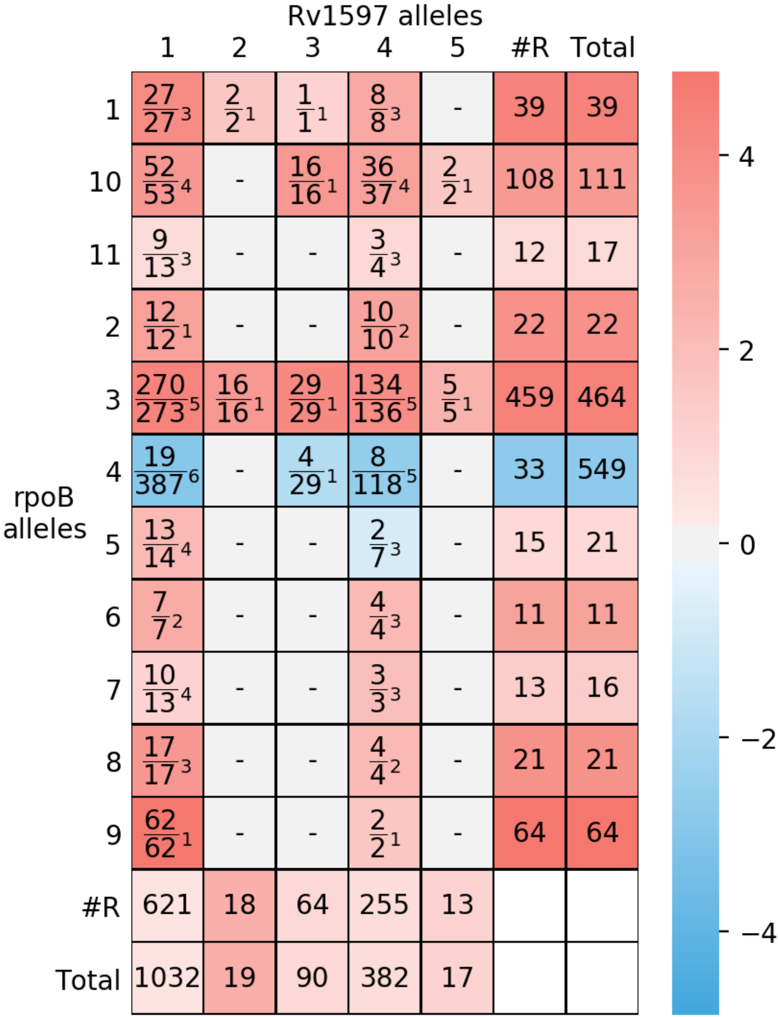
Rv1597 alleles
1 2 3 4 5 #R Total
rpoB alleles
1: 27/27 3 | 2/2 1 | 1/1 1 | 8/8 3 | - | 39 | 39
10: 52/53 4 | - | 16/16 1 | 36/37 4 | 2/2 1 | 108 | 111
11: 9/13 3 | - | - | 3/4 3 | - | 12 | 17
2: 12/12 1 | - | - | 10/10 2 | - | 22 | 22
3: 270/273 5 | 16/16 1 | 29/29 1 | 134/136 5 | 5/5 1 | 459 | 464
4: 19/387 6 | - | 4/29 1 | 8/118 5 | - | 33 | 549
5: 13/14 4 | - | - | 2/7 3 | - | 15 | 21
6: 7/7 2 | - | - | 4/4 3 | - | 11 | 11
7: 10/13 4 | - | - | 3/3 3 | - | 13 | 16
8: 17/17 3 | - | - | 4/4 2 | - | 21 | 21
9: 62/62 1 | - | - | 2/2 1 | - | 64 | 64
#R: 621 | 18 | 64 | 255 | 13
Total: 1032 | 19 | 90 | 382 | 17
4
2
0
−2
−4

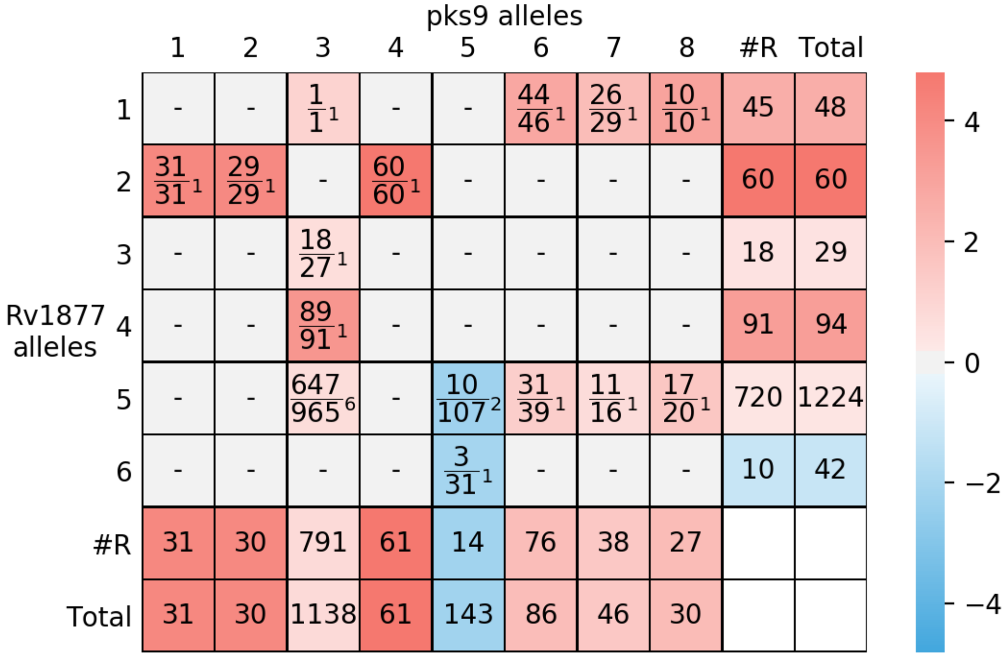
pks9 alleles
1
2
3
4
5
6
7
8
#R
Total
Rv1877 alleles
1
-
-
1/1 1
-
-
44/46 1
26/29 1
10/10 1
45
48
2
31/31 1
29/29 1
-
60/60 1
-
-
-
-
60
60
3
-
-
18/27 1
-
-
-
-
-
18
29
4
-
-
89/91 1
-
-
-
-
-
91
94
5
-
-
647/965 6
-
10/107 2
31/39 1
11/16 1
17/20 1
720
1224
6
-
-
-
-
3/31 1
-
-
-
10
42
#R
31
30
791
61
14
76
38
27
Total
31
30
1138
61
143
86
46
30
4
2
0
−2
−4

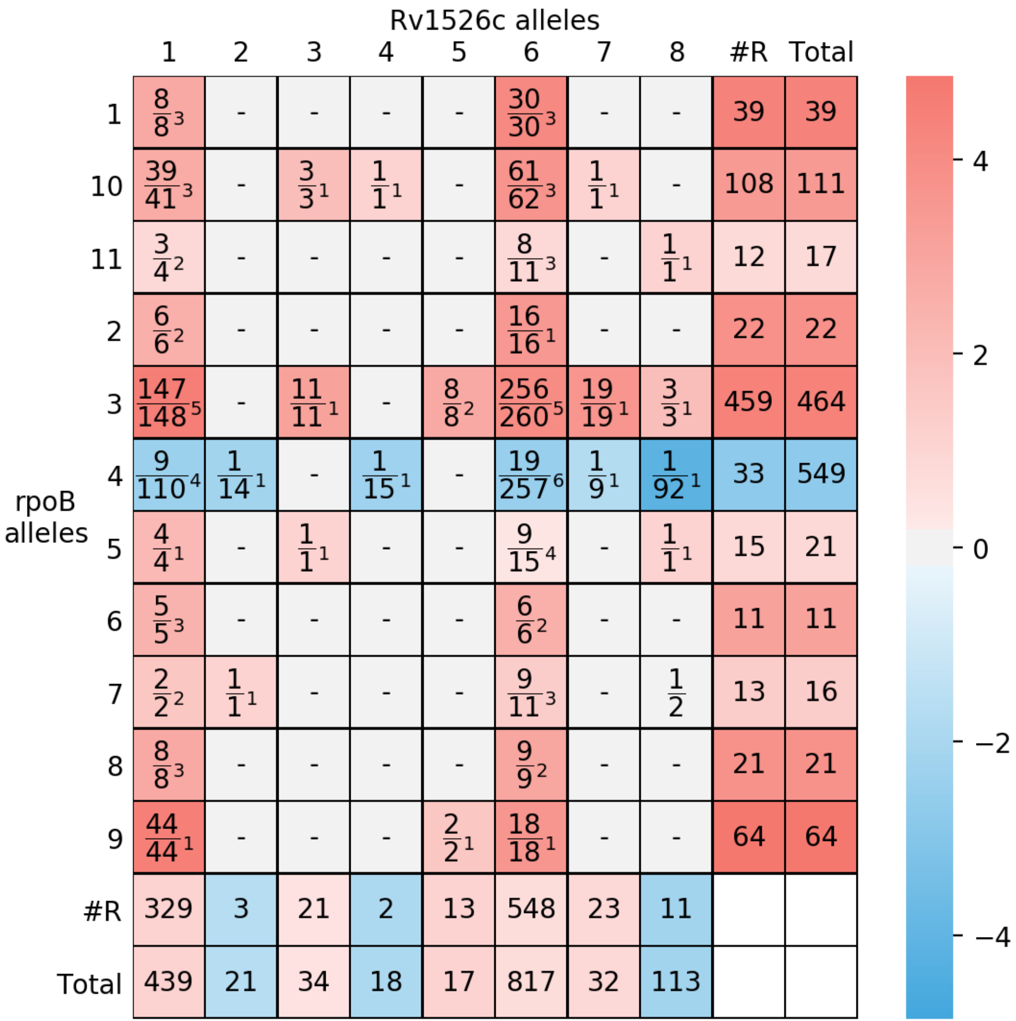
Rv1526c alleles
1 2 3 4 5 6 7 8 #R Total
rpoB alleles
1: 8/8 3, -, -, -, -, 30/30 3, -, -, 39, 39
10: 39/41 3, -, 3/3 1, 1/1 1, -, 61/62 3, 1/1 1, -, 108, 111
11: 3/4 2, -, -, -, -, 8/11 3, -, 1/1 1, 12, 17
2: 6/6 2, -, -, -, -, 16/16 1, -, -, 22, 22
3: 147/148 5, -, 11/11 1, -, 8/8 2, 256/260 5, 19/19 1, 3/3 1, 459, 464
4: 9/110 4, 1/14 1, -, 1/15 1, -, 19/257 6, 1/9 1, 1/92 1, 33, 549
5: 4/4 1, -, 1/1 1, -, -, 9/15 4, -, 1/1 1, 15, 21
6: 5/5 3, -, -, -, -, 6/6 2, -, -, 11, 11
7: 2/2 2, 1/1 1, -, -, -, 9/11 3, -, 1/2, 13, 16
8: 8/8 3, -, -, -, -, 9/9 2, -, -, 21, 21
9: 44/44 1, -, -, -, 2/2 1, 18/18 1, -, -, 64, 64
#R: 329, 3, 21, 2, 13, 548, 23, 11
Total: 439, 21, 34, 18, 17, 817, 32, 113
4
2
0
−2
−4

rpoB alleles \ rpoC alleles	1	2	3	4	5	6	7	8	9	10	11	12	#R	Total
1	-	-	$\frac{9}{9}^{1}$	-	-	$\frac{28}{28}^{3}$	-	-	-	-	-	-	39	39
10	-	-	$\frac{13}{14}^{1}$	-	-	$\frac{92}{94}^{3}$	-	-	-	-	$\frac{1}{1}^{1}$	$\frac{2}{2}^{1}$	108	111
11	-	-	$\frac{7}{8}^{2}$	-	-	$\frac{4}{7}^{3}$	-	-	-	-	$\frac{1}{1}^{1}$	-	12	17
2	-	-	$\frac{19}{19}^{1}$	-	$\frac{1}{1}^{1}$	-	-	-	-	-	-	-	22	22
3	$\frac{14}{14}^{3}$	$\frac{34}{34}^{3}$	$\frac{5}{5}^{1}$	$\frac{21}{21}^{1}$	$\frac{17}{17}^{1}$	$\frac{87}{91}^{4}$	$\frac{40}{40}^{1}$	$\frac{45}{45}^{1}$	$\frac{12}{12}^{3}$	$\frac{12}{12}^{1}$	-	$\frac{2}{2}^{1}$	459	464
4	-	-	$\frac{3}{53}^{2}$	-	$\frac{3}{32}^{2}$	$\frac{20}{307}^{6}$	-	-	-	-	$\frac{1}{92}^{1}$	$\frac{2}{33}^{1}$	33	549
5	-	-	-	-	-	$\frac{13}{16}^{2}$	-	-	-	-	$\frac{1}{1}^{1}$	-	15	21
6	-	-	$\frac{1}{1}^{1}$	-	-	$\frac{7}{7}^{2}$	-	-	-	-	-	-	11	11
7	-	-	-	-	-	$\frac{7}{8}^{3}$	-	-	-	-	-	$\frac{2}{3}^{1}$	13	16
8	-	-	$\frac{6}{6}^{1}$	-	$\frac{1}{1}^{1}$	$\frac{13}{13}^{2}$	-	-	-	-	-	-	21	21
9	-	-	-	-	-	$\frac{64}{64}^{1}$	-	-	-	-	-	-	64	64
#R	14	35	79	21	30	470	40	45	12	12	4	8		
Total	14	35	135	21	60	788	40	45	12	12	110	42		

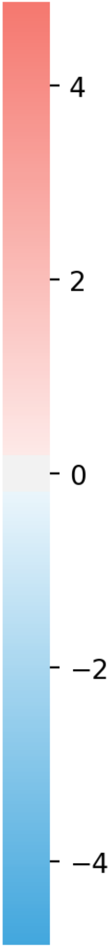

Supplement: Supplementary file 8 — Supplementary Data 5 [file 41467_2018_6634_MOESM8_ESM.zip › Supplementary Data 5/rifampicin_epistasis.pdf]
